# Entwicklung von Qualitätsindikatoren für die Versorgung von Patient:innen mit chronischer Nierenerkrankung: Ergebnisse einer strukturierten Konsensfindung mittels Delphi-Verfahrens

**DOI:** 10.1007/s00103-023-03700-9

**Published:** 2023-05-16

**Authors:** Elizabeth Sierocinski, Lina Dröge, Jean-François Chenot, Natalie Ebert, Elke Schäffner, Tim Bothe, Nina Mielke, Sylvia Stracke, Simone Kiel

**Affiliations:** 1 Institut für Community Medicine, Abteilung Allgemeinmedizin, Universitätsmedizin Greifswald, Greifswald, Mecklenburg-Vorpommern Deutschland; 2 Institut für Public Health, Charité – Universitätsmedizin Berlin, Berlin, Deutschland; 3 Klinik und Poliklinik für Innere Medizin A, Universitätsmedizin Greifswald, Greifswald, Mecklenburg-Vorpommern Deutschland

**Keywords:** Qualitätsüberprüfung, Eingeschränkte Nierenfunktion, Chronische Niereninsuffizienz, Expertenkonsens, Ambulante Versorgung, Quality assessment, Impaired renal function, Chronic renal failure, Expert consensus, Outpatient care

## Abstract

**Hintergrund:**

Die chronische Nierenkrankheit (CKD) ist eine häufige Erkrankung, insbesondere im höheren Alter. Um der Progression der Erkrankung und deren Komplikationen vorzubeugen, ist eine leitliniengerechte ambulante Versorgung von Patient:innen mit CKD anzustreben. Zur Messung und Bewertung der Versorgungsqualität können Qualitätsindikatoren (QI) genutzt werden. In Deutschland existieren bisher keine QI für CKD. Ziel der Arbeit war die Entwicklung von QI für die Qualitätsüberprüfung der ambulanten Versorgung von Patient:innen über 70 Jahren mit nichtdialysepflichtiger CKD.

**Material und Methoden:**

Auf Grundlage der nationalen S3-Leitlinie CKD und eines Reviews internationaler QI wurde eine Liste von QI erstellt. Die ausgewählten QI wurden in 2 Sets eingeteilt: basierend auf Routinedaten (z. B. Abrechnungsdaten der Krankenkassen) und auf Datenerhebung in der Praxis (Chart-Review). Expert:innen verschiedener Fachrichtungen sowie ein Patient:innenvertreter bewerteten diese in einem Delphi-Verfahren mit 2‑stufiger Onlinebefragung im Oktober 2021 und Januar 2022 und abschließender Konsensuskonferenz im März 2022. Zusätzlich wurden Ranglisten der wichtigsten QI von jedem Set erstellt.

**Ergebnisse:**

Ein Inzidenz- und ein Prävalenzindikator wurden a priori festgelegt und standen nicht zur Abstimmung. Weitere 21 QI standen zur Abstimmung durch die Expert:innen. Für jedes QI-Set wurden die 7 wichtigsten Indikatoren ausgewählt. Nur 1 QI wurde von dem Expert:innenpanel für den zusätzlichen Einsatz bei Erwachsenen unter 70 Jahren als nicht geeignet eingestuft.

**Diskussion:**

Die QI sollen es ermöglichen, die Qualität der ambulanten Versorgung von Patient:innen mit CKD zu untersuchen, mit dem Ziel, die leitlinienkonforme ambulante Versorgung zu optimieren.

**Zusatzmaterial online:**

Zusätzliche Informationen sind in der Online-Version dieses Artikels (10.1007/s00103-023-03700-9) enthalten.

## Abstract (en)

**Background:**

Chronic kidney disease (CKD) is a common condition, especially in the elderly. In order to prevent progression and complications of the disease, guideline-adherent outpatient care of patients with CKD should be prioritized. Quality indicators (QIs) can be used to measure and evaluate the quality of ambulatory care for patients with CKD. QIs specifically made for evaluating CKD care in Germany are not yet available. The goal of this work was to develop QIs for the quality assessment of outpatient care for patients over the age of 70 with CKD not requiring dialysis.

**Materials and methods:**

QIs were operationalized from the recommendations of the German national guideline for CKD and others were proposed based on a published review of international QIs. The resulting QIs were divided into sets based on routine data (e.g., health insurance billing data) and data collection in practices (chart review). A panel of experts from various disciplines as well as a patient representative evaluated the proposed QIs in a two-stage Delphi process via online survey in October 2021 and January 2022 and a final consensus conference in March 2022. In addition, ranking lists of the most important QIs from each set were created.

**Results:**

An incidence indicator and a prevalence indicator were established; these were not subject to vote. Further, 21 QIs were voted upon by the expert panel. The seven most important QIs in each set (billing data or chart review) were selected. Only one QI was rated by the expert panel as not suitable for additional use in adults under the age of 70 years.

**Discussion:**

The QIs will enable the evaluation of the quality of outpatient care for patients with CKD with the long-term aim of optimizing guideline-adherent outpatient care.

## Hintergrund

Handlungsempfehlungen für die ambulante Versorgung von nichtdialysepflichtigen Patient:innen mit chronischer Nierenerkrankung (CKD) liegen vor [1]. Um die Umsetzung dieser Empfehlungen zu evaluieren, werden Qualitätsindikatoren (QI) benötigt, die die Qualität der ambulanten Versorgung von Patient:innen mit CKD anhand von Routinedaten (Abrechnungsdaten z. B. von Krankenkassen) und/oder einem Chart-Review (Datenerhebung in der Arztpraxis z. B. durch Überprüfung der Krankenakten von Patient:innen) abbilden können. Im Gegensatz zu anderen häufigen Erkrankungen, wie arterieller Hypertonie und Diabetes mellitus, existieren in Deutschland bisher keine QI für CKD [2]. Dieser Artikel beschreibt die Entwicklung von QI für die ambulante Versorgung von CKD-Patient:innen in Deutschland mittels eines interdisziplinären, RAND-(Research-and-Development‑)modifizierten Delphi-Prozesses.

Aufgrund der Häufigkeit von CKD im höheren Alter (ca. 12 % der deutschen Bevölkerung im Alter von 70–79 Jahren [3]) und der medizinischen Komplexität (Multimorbidität) dieser Gruppe wurden ältere Menschen als besonders vulnerable Risikogruppe identifiziert. Die Entwicklung von QI stellt das erste Modul des vierteiligen Projekts *Leitliniengerechte Versorgung alter Patienten mit chronischer Nierenerkrankung* (Akronym: GUIDAGE-CKD) dar. Diese QI werden in den nächsten Phasen des Projekts validiert und in Routinedaten eingesetzt, um die ambulante Versorgung, insbesondere die hausärztliche Versorgung von CKD-Patient:innen, zu untersuchen. Ziel ist es, Hindernisse und Lösungsansätze bei der Leitlinienimplementierung als Grundlage für Interventionen zur Optimierung der ambulanten Versorgung von CKD-Patient:innen zu ermitteln.

## Methoden

### Entwicklung von Qualitätsindikatoren (QI)

Es wurden potenzielle QI durch Literaturrecherche und auf Grundlage von Leitlinien formuliert, angelehnt an Empfehlungen von Deckert et al. [4].

Aufgrund der Relevanz in der ambulanten Versorgung wurde vor allem die deutsche S3-Leitlinie „Versorgung von Patienten mit chronischer nicht-dialysepflichtiger Nierenerkrankung in der Hausarztpraxis“ [1] als Grundlage für die QI herangezogen. Bei dieser Leitlinie handelt es sich um eine Metaleitlinie mit Empfehlungen angelehnt an bestehende evidenzbasierte Leitlinien (z. B. Kidney Disease Improving Global Outcomes – KDIGO, National Institute for Health and Care Excellence – NICE; [5, 6]). Die insgesamt 50 Empfehlungen wurden auf ihre Operationalisierbarkeit geprüft [1]. Dabei spielte unter anderem die Messbarkeit mittels Routinedaten oder Chart-Review eine Rolle. Die Auswahl erfolgte nach dem SMART-Prinzip (spezifisch, messbar, akzeptabel, realistisch, terminiert; [7]). Ausgewählte Empfehlungen wurden in potenzielle QI umformuliert.

Danach wurde eine selektive Literaturrecherche nach bereits bestehenden QI in den medizinischen Datenbanken PubMed und Embase durchgeführt (Suchbegriffe: „chronic kidney disease“, „ckd“ und „quality indicators“). Die Publikationen von Organisationen im Bereich Qualitätssicherung (u. a. Institute for Clinical Evaluative Sciences – ICES, NICE Quality and Outcomes Framework – QOF) wurden auf empfohlene Indikatoren überprüft.

Basierend auf den ausgewählten QI wurde eine Liste potenzieller QI für die Zielgruppe von Patient:innen über 70 Jahren erstellt. Die entwickelten QI waren entweder positiv (höherer Erfüllungsgrad weist auf gute Versorgung hin) oder negativ formuliert (geringerer Erfüllungsgrad weist auf gute Versorgung hin).

Um die Messbarkeit zu ermöglichen, wurden für jeden Indikator Zähler und Nenner definiert. Zusätzlich wurde ein Informationstext zur Erläuterung möglicher Probleme bei der praktischen Anwendung der Indikatoren verfasst. Bereits von anderen internationalen Organisationen (ICES, National Quality Framework – NQF) vorgeschlagene QI wurden in dem Informationstext entsprechend beschrieben.

### Konsensverfahren

Es wurde ein Konsensverfahren, angelehnt an Deckert et al., mit 2‑stufiger Onlinebefragung und abschließender Konsensuskonferenz durchgeführt [4]. Hierfür wurde ein Panel bestehend aus einem Patient:innenvertreter sowie 5 Expert:innen mit jeweils einem/einer Vertreter:in aus folgenden Gebieten zusammengestellt: praktische Nephrologie, akademische Nephrologie, praktische Allgemeinmedizin (bzw. Deutsche Gesellschaft für Allgemeinmedizin und Familienmedizin –DEGAM), akademische Allgemeinmedizin (bzw. DEGAM) und Geriatrie. Ziel der interdisziplinären Zusammensetzung war es, eine repräsentative Evaluation der vorgeschlagenen QI durch die Einbeziehung der Expert:innen und Anwender:innen zu ermöglichen.

#### 2-stufige Onlinebewertung

Vorab wurden den Expert:innen ein Video zur Erklärung des Bewertungsinstruments und allgemeine Informationen zum Ablauf des Projekts zugesandt. Die Delphi-Befragungsrunden wurden mittels Online-Befragungstool SoSci Survey [8] durchgeführt (der Fragebogen ist im Onlinematerial 1 einsehbar). Der Befragungszeitraum betrug für jede Runde 4 Wochen; die erste Befragung fand Oktober 2021 und die zweite Januar 2022 statt. Falls notwendig, wurden die Expert:innen eine Woche vor Ablauf der Frist telefonisch oder per E‑Mail erinnert.

Die vorgeschlagenen QI wurden mit Hilfe der methodischen Gütekriterien angelehnt an das Instrument QUALIFY in den 3 Domänen Relevanz, Wissenschaftlichkeit und Praktikabilität [9] von den 6 Expert:innen auf einer Likert-Skala von 1 (stimme nicht zu) bis 9 (stimme voll zu) bewertet. Die Definitionen der 3 Kategorien sind im Onlinematerial 2 beschrieben. Eine Kommentarfunktion erlaubte die Begründung der Bewertung und/oder Empfehlungen für Änderungen. Ebenfalls bestand die Möglichkeit der Enthaltung.

Die Bewertungen wurden nach einem festgelegten Algorithmus, angelehnt an die RAND/UCLA–Methodologie zur Güteprüfung von QI, ausgewertet [10–12]. Hierbei wurde für alle QI-Bewertungen (auf einer Skala von 1–9) der jeweilige Median gebildet. QI-Bewertungen mit Medianen von 1 bis 3 wurden als irrelevant, nicht wissenschaftlich und/oder nicht praktikabel eingestuft. Mediane von 4 bis 6 wurden als unentschieden gewertet. Mediane zwischen 7–9 wurden als relevant, wissenschaftlich und/oder praktikabel definiert. Das Kriterium für Dissens war eine Streuung der Bewertungen, in der sich mehr als 2 Bewertungen in jedem Extrem (1–3 und 7–9) befanden [12]. Eine positive Übereinstimmung wurde als Median zwischen 7 und 9 ohne Dissens definiert; diese QI wurden als geeignet (relevant, wissenschaftlich, praktikabel) eingestuft. QI mit einem Median zwischen 1 und 3 ohne Dissens wurden als nicht geeignet (irrelevant, nicht wissenschaftlich, nicht praktikabel) definiert. QI mit Dissens oder mit einem Median von 4 bis 6 galten als unsicher mit Diskussionsbedarf in der Konsensuskonferenz [11].

Nach Abschluss der 1. Delphi-Runde wurden die Mediane mithilfe des Statistikprogramms SAS 9.4 ermittelt. Kommentare wurden durch das Autor:innenteam diskutiert und führten gegebenenfalls zu einer Modifizierung der QI. Anschließend erhielten die Expert:innen eine Rückspiegelung der Ergebnisse, in der sowohl die individuellen Antworten als auch die Gesamtauswertung, alle Kommentare und Modifizierungen der QI abgebildet wurden. Anhand dieser Auswertung sollten die Expert:innen ihre Bewertung anhand der Rückspiegelung aller Bewertungen in der darauffolgenden zweiten Delphi-Runde anpassen oder beibehalten.

#### Konsensuskonferenz

Die endgültige Abstimmung und Konsentierung der QI fand im März 2022 unter neutraler Moderation durch einen von der Arbeitsgemeinschaft der Wissenschaftlichen Medizinischen Fachgesellschaften e. V. (AWMF) zertifizierten Moderator in einem nominalen Gruppenprozess statt. Für jeden QI wurden ausschließlich die Ergebnisse der zweiten Delphi-Runde präsentiert. QI, für die es übereinstimmende Bewertungen gegeben hatte, wurden mit finaler Abstimmung bestätigt. QI mit Diskussionsbedarf wurden im nominalen Gruppenprozess debattiert. Als Konsenskriterium galt die Zustimmung von > 75 % innerhalb des Expert:innenpanels. Nur QI, die in der finalen Abstimmung als relevant, wissenschaftlich und praktikabel eingestuft wurden, wurden in die finalen Sets aufgenommen.

#### Priorisierung der QI

Anschließend erfolgte eine Priorisierung der relevantesten QI durch die Expert:innen. Hierbei wurden die beiden QI-Sets (QI jeweils basierend auf Routinedaten und Chart-Review) separat betrachtet. Mittels eines nominalen Gruppenprozesses wurden die jeweils 6 wichtigsten QI identifiziert. Ein zusätzlicher siebter QI wurde für jede Datenquelle für den Fall der Nichtverfügbarkeit eines der ersten 6 QI ausgewählt.

Um zukünftige Evaluationen der ambulanten Versorgung von CKD-Erkrankten außerhalb der Zielaltersgruppe zu ermöglichen, wurde die Einsetzbarkeit der QI bei Erwachsenen unter 70 Jahren ebenfalls abgestimmt.

## Ergebnisse

### Ergebnisse der Literaturrecherche

Die Literaturrecherche ergab eine vorläufige Liste von 23 QI mit Definitionen sowie Angaben zur Verfügbarkeit in Chart-Review und/oder Routinedaten (Tab. 1). Ausführliche Definitionen mit Zähler und Nenner, mögliche Probleme bei der Anwendung sowie Hintergrundinformationen sind in der Langfassung der QI verfügbar (Onlinematerial 3). Davon waren 15 sowohl aus Routinedaten als auch aus Chart-Review, 6 nur aus Chart-Review und 2 nur aus Routinedaten extrahierbar. 18 QI basierten auf Leitlinienempfehlungen, z. B. aus der Leitlinie „Versorgung von Patienten mit chronischer nicht-dialysepflichtiger Nierenerkrankung in der Hausarztpraxis“ [1], NICE [6] und KDIGO [5]. 3 QI wurden aus bereits bestehenden QI kanadischer [13–15], japanischer [16], belgischer [11] und US-amerikanischer Publikationen [17] adaptiert.

**Table 1:** 

Nr.	Indikator	Datenquelle	
		Routinedaten	Chart-Review
1	Prävalenzindikator	☒	☒
2	Inzidenzindikator	☒	☒
3	Erstdiagnose CKD mit 2 eGFR-Messungen im Abstand von 3 Monaten	☐	☒
4	Erstdiagnose CKD mit 2 eGFR-Messungen im Abstand von 3 Monaten	☒	☐
5	Patienten mit Diabetes mit mind. 1 × jährlich eGFR-Bestimmung	☒	☒
6	Patienten mit Erstdiagnose Bluthochdruck mit eGFR-Bestimmung	☒	☒
7	Patienten mit Erstdiagnose Bluthochdruck mit Urinuntersuchung auf Protein oder Albumin	☒	☒
8	Erstdiagnose CKD und Bestimmung der Albumin-Kreatinin-Ratio	☒	☒
9	Erstdiagnose CKD und Untersuchung auf Hämaturie mittels Streifentests	☒	☒
10	Erstdiagnose CKD und Kontrolle des Blutdrucks	☐	☒
11	Patienten mit CKD und unter 80 Jahren, deren Blutdruck auf ≤ 140 mm Hg eingestellt ist	☐	☒
12	Verordnung NSAR bei eGFR < 30 ml/min/1,73 m^2^ bei Patienten mit CKD	☒	☒
13	Monitoring eGFR anhand Monitoringintervalle bei Patienten mit CKD (siehe Abbildung in S3-Leitlinie^a^)	☐	☒
14	Kontrolle eGFR 1 × jährlich (Stadium 1–3) bzw. 2 × jährlich (Stadium 4) bei CKD-Patienten	☒	☐
15	Jährliche Blutdruckmessung bei CKD-Patienten	☐	☒
16	Jährliche Albumin-Kreatinin-Ratio-Messung bei Patienten mit CKD und nachgewiesener Proteinurie	☒	☒
17	Patienten mit CKD ohne bekannte Anämie und jährlicher (Stadium 3) bzw. halbjährlicher (Stadium 4) Hämoglobinwertmessung	☒	☒
18	Überweisung zum Nephrologen von Patienten mit eGFR < 30 ml/min/1,73 m^2^	☒	☒
19	Metformin-Verordnung bei Patienten mit Diabetes und CKD und einer eGFR < 30 ml/min/1,73 m^2^	☒	☒
20	CKD und Anämie mit Bestimmung von Ferritin und Serumtransferrinsättigung	☐	☒
21	Therapie mit ACE-Hemmern oder ARB bei Patienten mit CKD und Hypertonie	☒	☒
22	Influenzaimpfung bei CKD	☒	☒
23	Gleichzeitige Therapie mit ACE-Hemmern und ARB	☒	☒

Diese Tabelle stellt die Kurzversion der QI dar, für die Langversion siehe Onlinematerial 1

*ACE* Angiotensin-Converting-Enzym, *ARB* Angiotensin-Rezeptor-Blocker, *eGFR* Estimated Glomerular Filtration Rate, *NSAR* nichtsteroidale Antirheumatika

^a^ Siehe S3-Leitlinie CKD ([1, S. 87], Abb. 2, adaptiert nach [6])

### Inzidenz‑/Prävalenzindikatoren

Ein Inzidenzindikator und ein Prävalenzindikator wurden definiert. Der Inzidenzindikator wurde entwickelt, um Informationen über die Anzahl der neuen CKD-Diagnosen im Beobachtungszeitraum zu liefern, und wurde als Nenner für die QI eingesetzt, die sich auf neu diagnostizierte CKD-Patient:innen beziehen. Der Prävalenzindikator zeigt die Anzahl aller Patient:innen mit CKD und diente als Nenner für diejenigen QI, die sich auf alle CKD-Patient:innen beziehen.

### Delphi-Ergebnisse

Der erste Delphi-Fragebogen mit der Liste von 23 potenziellen QI wurde im Oktober 2021 an die Expert:innen versandt. Nach Auswertung der Ergebnisse wurde angelehnt an die Kommentare der Expert:innen die Formulierung der QI angepasst. Tab. 2 zeigt die Ergebnisse der Delphi-Befragungen mit den modifizierten QI. Tab. 3 zeigt die aus dem Konsens resultierenden Änderungen der QI 9, 11 und 22. Bei QI 12 wurde im Hintergrundtext ein Problem bei möglicher Anwendung in der Praxis ergänzt. Bei QI 1 wurde die Beschreibung des Zählers erweitert (Onlinematerial 3).

**Table 2:** 

QI	Delphi-Runde 1			Delphi-Runde 2			Konsensuskonferenz		
	R	W	P	R	W	P	Zustimmung	Ergebnis	Einsetzbarkeit bei Patienten unter 70 Jahren
1. Prävalenzindikator	–	–	–	–	–	–	100 % (6/6)	Geeignet	Ja
2. Inzidenzindikator	–	–	–	–	–	–	100 % (6/6)	Geeignet	Ja
3. Erstdiagnose CKD mit 2 eGFR-Messungen im Abstand von 3 Monaten (Chart-Review)	8,5	9	8,5	8,5	9	8	100 % (6/6)	Geeignet	Ja
4. Erstdiagnose CKD mit 2 eGFR-Messungen im Abstand von 3 Monaten (Routinedaten)	9	9	9	8,5	9	9	100 % (6/6)	Geeignet	Ja
5. Patienten mit Diabetes mit mind. 1 × jährlich eGFR-Bestimmung	9	9	9	9	9	9	100 % (6/6)	Geeignet	Ja
6. Patienten mit Erstdiagnose Bluthochdruck mit eGFR-Bestimmung	9	9	8,5	9	9	9	100 % (6/6)	Geeignet	Ja
7. Patienten mit Erstdiagnose Bluthochdruck mit Urinuntersuchung auf Protein oder Albumin	8,5	8	8,5	9	9	9	100 % (6/6)	Geeignet	Ja
8. Erstdiagnose CKD und Bestimmung der Albumin-Kreatinin-Ratio	9	9	8,5	9	9	9	100 % (5 Zustimmungen, 1 Enthaltung)	Geeignet	Ja
9. Erstdiagnose CKD und Untersuchung auf Hämaturie mittels Streifentests	8,5	9	8	9	8,5	9	100 % (6/6)	Geeignet	Ja
10. Erstdiagnose CKD und Kontrolle des Blutdrucks	9	9	8	9	9	8	100 % (6/6)	Geeignet	Ja
11. Patienten mit CKD und unter 80 Jahren, deren Blutdruck auf ≤140 mm Hg eingestellt ist	9	8	7	9	8	7,5	100 % (6/6)	Geeignet	Ja
12. Verordnung NSAR bei eGFR < 30 ml/min/1,73 m^2^ bei Patienten mit CKD	8,5	7,5	6,5	9	8	7	100 % (6/6)	Geeignet	Ja
13. Monitoring eGFR anhand Monitoringintervalle bei Patienten mit CKD (siehe Abbildung^a^)	9	8	8	9	8	8,5	100 % (6/6)	Geeignet	Ja
14. Kontrolle eGFR 1 × jährlich (Stadium 1–3) bzw. 2 × jährlich (Stadium 4) bei CKD-Patienten	8,5	7,5	8	9	9	8,5	100 % (6/6)	Geeignet	Ja
15. Jährliche Blutdruckmessung bei CKD-Patienten	9	9	9	9	9	8,5	100 % (6/6)	Geeignet	Ja
16. Jährliche Albumin-Kreatinin-Ratio-Messung bei Patienten mit CKD und nachgewiesener Proteinurie	7,5	7	6,5	8	8	6,5	100 % (5 Zustimmungen, 1 Enthaltung)	Geeignet	Ja
17. Patienten mit CKD ohne bekannte Anämie und jährlicher (Stadium 3) bzw. halbjährlicher (Stadium 4) Hämoglobinwertmessung	8	9	8	8,5	8,5	7,5	100 % (6/6)	Geeignet	Ja
18. Überweisung zum Nephrologen von Patienten mit eGFR < 30 ml/min/1,73 m^2^	9	8,5	6	9	8	7,5	80 % (4 Zustimmungen, 1 Enthaltung, 1 Ablehnung)	Geeignet	Nein
19. Metformin-Verordnung bei Patienten mit Diabetes und CKD und einer eGFR < 30 ml/min/1,73 m^2^	8,5	7,5	8,5	8,5	9	8,5	83,3 % (5 Zustimmungen, 1 Ablehnung)	Geeignet	Ja
20. CKD und Anämie mit Bestimmung von Ferritin und Serumtransferrinsättigung	8	7,5	7	8	8	8,5	100 % (5 Zustimmungen, 1 Enthaltung)	Geeignet	Ja
21. Therapie mit ACE-Hemmern oder ARB bei Patienten mit CKD und Hypertonie	9	9	9	8,5	9	9	100 % (6/6)	Geeignet	Ja
22. Influenzaimpfung bei CKD	7,5	7	7,5	8	8	8	100 % (6/6)	Geeignet	Ja
23. Gleichzeitige Therapie mit ACE-Hemmern und ARB	9	9	8,5	9	9	8,5	100 % (6/6)	Geeignet	Ja

*ACE* Angiotensin-Converting-Enzym, *ARB* Angiotensin-Rezeptor-Blocker, *CKD* chronische Nierenkrankheit, *eGFR* Estimated Glomerular Filtration Rate, *NSAR* nichtsteroidale Antirheumatika, *P* Praktikabilität, *R* Relevanz, *W* Wissenschaftlichkeit

^a^Siehe S3-Leitlinie CKD ([1, S. 87], Abb. 2, adaptiert nach [6])

**Table 3:** 

Modifizierungszeitpunkt	QI Nr.	Finale Formulierung des QI	Änderungen
1. Delphi-Runde	11	Anteil der Patienten ≥ 70 Jahre mit CKD (eGFR < 60 ml/min/1,73 m^2^) unter 80 Jahren, deren systolischer Blutdruck auf ≤ 140 mm Hg eingestellt ist	„< 140 mm Hg“ wurde in „≤ 140 mm Hg“ geändert
Konsensuskonferenz	9	Anteil der Patienten ≥ 70 Jahre mit Erstdiagnose CKD (Inzidenzindikator), bei denen eine Urinuntersuchung auf Hämaturie mittels Streifentest erfolgte	Der Hintergrund zur Durchführung wurde spezifiziert (Ergänzung: „auf Hämaturie“)
Konsensuskonferenz	11	Anteil der Patienten ≥ 70 Jahre mit CKD (eGFR < 60 ml/min/1,73 m^2^) unter 80 Jahren, deren systolischer Blutdruck auf ≤ 140 mm Hg eingestellt ist	„Aber“ wurde in der Formulierung gestrichen
Konsensuskonferenz	22	Anteil der Patienten ≥ 70 Jahre mit CKD, die in den letzten 12 Monaten eine Grippeschutzimpfung erhalten haben	„Falls nicht kontraindiziert“ wurde gestrichen. Es sollte generell keine Maßnahme durchgeführt werden, wenn sie für die Patienten kontraindiziert ist

*CKD* chronische Nierenkrankheit

Nach Auswertung der ersten Delphi-Befragungsrunde startete die zweite Delphi-Runde im Januar 2022, nach deren Abschluss keiner der Indikatoren umformuliert wurde. In Vorbereitung auf die Konsensuskonferenz wurden die QI in die Kategorien „geeignet“, „unsicher“ und „ungeeignet“ eingeteilt. Als geeignet galten 19 der QI. 2 Indikatoren wurden als unsicher kategorisiert: Bei QI 16 lag der Median in der Kategorie Praktikabilität unter 7, bei QI 18 ergab sich ein Dissens in der Kategorie Wissenschaftlichkeit: Die Schnittstellendefinition, ab welcher geschätzten glomerulären Filtrationsrate (Estimated Glomerular Filtration Rate – eGFR) eine Überweisung in die Nephrologie erfolgen sollte, ist möglicherweise je nach Patient:in und weiteren Faktoren (u. a. Lebenserwartung, Komorbiditäten, weitere Symptome, Progression der Erkrankung und Komplikationen) unterschiedlich. Kein QI wurde als ungeeignet eingestuft.

Die Antwortrate für beide Delphi-Runden betrug 100 %. Es wurde von den Expert:innen weder eine Empfehlung abgegeben, QI zu streichen, noch wurden neue QI vorgeschlagen.

### Konsensuskonferenz

Die finale Abstimmung der QI fand in einer Konsensuskonferenz im März 2022 statt. In der Diskussion wurden alle 23 QI abschließend als geeignet bewertet (Tab. 2). Insgesamt 18 der QI wurden dabei einstimmig als geeignet befunden. Bei 3 QI wurden Änderungen in der Formulierung vorgenommen (Tab. 3). Zum einen wurde beschlossen, dass bei QI 9 der Beweggrund zur Durchführung des Streifentests klarer ersichtlich sein sollte, da dieser auch auf andere Parameter testen kann (Glucose, Nitrit, Eiweiß usw.). Daher wurde der QI „Streifentest auf Hämaturie“ ergänzt. Zum anderen wurde diskutiert, dass keine der Maßnahmen und Therapien bei bestehender Kontraindikation durchgeführt werden sollte. Daher wurde die überflüssige Formulierung „falls nicht kontraindiziert“ bei QI 22 bezüglich der Durchführung der Grippeschutzimpfung gestrichen. Bei QI 18 wurde eine Umformulierung diskutiert in: „Anteil der Patient:innen ≥ 70 Jahre, *die nicht als palliativ eingestuft wurden,* mit CKD und erstmals festgestellter eGFR < 30 ml/min/1,73 m^2^, die an einen Nephrologen überwiesen wurden“. Die Mehrheit der Expert:innen stimmte aber für die Originalversion, sodass diese beibehalten wurde.

Die Einsetzbarkeit der QI für erwachsene Patient:innen unter 70 Jahren wurde vom Expert:innenpanel bei 22 von 23 QI bestätigt (Tab. 2). Tab. 4 zeigt die finale QI-Liste und die Einsetzbarkeit der QI bei Erwachsenen unter 70 Jahren. Tab. 5 zeigt die Ergebnisse der Priorisierung nach Datenquelle.

**Table 4:** 

Nr.	QI	Datenquelle (Rangordnung 1–7)		Zähler	Nenner	Einsetzbarkeit < 70 Jahre
1	Anteil der Patienten ≥ 70 Jahre, bei denen eine chronische nichtdialysepflichtige Nierenerkrankung diagnostiziert wurde (Prävalenzindikator)	RD	CR	Anzahl der ≥ 70-jährigen Patienten mit diagnostizierter bzw. kodierter und nichtdialysepflichtiger CKD	Alle Patienten ≥ 70 Jahre	Ja
2	Anteil der Patienten ≥ 70 Jahre, bei denen eine chronische nichtdialysepflichtige Nierenerkrankung neu diagnostiziert wurde (Inzidenzindikator)	RD	CR	Anzahl der Patienten ≥ 70 Jahre mit neu diagnostizierter bzw. kodierter CKD, bei denen in den letzten 12 Monaten (4 Abrechnungsquartale vor Indexquartal) *keine* CKD diagnostiziert/kodiert wurde	Alle Patienten ≥ 70 Jahre, bei denen in den letzten 12 Monaten (4 Abrechnungsquartale vor Indexquartal) *keine* CKD diagnostiziert/kodiert wurde	Ja
3	Anteil der Patienten ≥ 70 Jahre mit erstmals festgestellter eGFR < 60 ml/min/1,73 m^2^, bei denen für die Erstdiagnose eine zweimalige Messung der eGFR im Abstand von 3 Monaten durchgeführt wurde	–	CR **(5)**	Anzahl der Patienten ≥ 70 Jahre mit erstmals festgestellter eGFR < 60 ml/min/1,73 m^2^, bei denen im Diagnosequartal 2 eGFR-Bestimmungen durchgeführt wurden oder eine weitere Messung im Folgequartal	Anzahl der Patienten ≥ 70 Jahre mit erstmals festgestellter eGFR < 60 ml/min/1,73 m^2^	Ja
4	Anteil der Patienten ≥ 70 Jahre mit neu diagnostizierter CKD, bei denen für die Erstdiagnose eine zweimalige Messung der eGFR im Abstand von 3 Monaten durchgeführt wurde	RD	–	Anzahl der Patienten ≥ 70 Jahre mit Erstdiagnose CKD (Inzidenzindikator), bei denen im Diagnosequartal 2 eGFR-Bestimmungen durchgeführt wurden oder eine weitere Messung im Vorquartal	Anzahl der Patienten ≥ 70 Jahre mit Erstdiagnose CKD (Inzidenzindikator)	Ja
5	Anteil der Patienten ≥ 70 Jahre mit Diabetes mellitus, bei denen mindestens einmal jährlich die eGFR bestimmt wurde	RD	CR	Anzahl der Patienten ≥ 70 Jahre mit Diabetes mellitus (ICD E11, E10), bei denen mindestens einmal innerhalb eines Jahres eine eGFR-Bestimmung erfolgte	Anzahl der Patienten ≥ 70 Jahre mit Diabetes mellitus (ICD E11, E10)	Ja
6	Anteil der Patienten ≥ 70 Jahre mit Erstdiagnose Bluthochdruck und ohne bekannte CKD, die eine Bestimmung der eGFR erhalten haben	RD	CR	Anzahl der Patienten ≥ 70 Jahre mit Erstdiagnose Bluthochdruck (ICD I10–I15, kodiert in 2 unterschiedlichen Quartalen innerhalb von 1 Jahr und mind. 4 Quartale vor Indexquartal keine Verschlüsselung), die eine Serumkreatinin-Bestimmung mit eGFR erhalten haben	Anzahl der Patienten ≥ 70 Jahre mit Erstdiagnose Bluthochdruck (ICD I10–I15, kodiert in 2 unterschiedlichen Quartalen innerhalb von 1 Jahr und mind. 4 Quartale vor Indexquartal keine Verschlüsselung)	Ja
7	Anteil der Patienten ≥ 70 Jahre mit Erstdiagnose Bluthochdruck und ohne bekannte CKD, die eine Urinuntersuchung auf Protein oder Albumin erhalten haben	RD	CR	Anzahl der Patienten ≥ 70 Jahre mit Erstdiagnose Bluthochdruck (ICD I10–I15, kodiert in 2 unterschiedlichen Quartalen innerhalb von 1 Jahr und mind. 4 Quartale vor Indexquartal keine Verschlüsselung), die eine Urinuntersuchung auf Eiweiß oder Albumin erhalten haben	Anzahl der Patienten ≥ 70 Jahre mit Erstdiagnose Bluthochdruck (ICD I10–I15, kodiert in 2 unterschiedlichen Quartalen innerhalb von 1 Jahr und mind. 4 Quartale vor Indexquartal keine Verschlüsselung)	Ja
8	Anteil der Patienten ≥ 70 Jahre mit Erstdiagnose CKD (Inzidenzindikator), bei denen eine Bestimmung der Albumin-Kreatinin-Ratio im Urin erfolgte	RD **(2)**	CR **(3)**	Anzahl der Patienten ≥ 70 Jahre mit Erstdiagnose CKD (Inzidenzindikator) und mindestens einer Bestimmung der Albumin-Kreatinin-Ratio im Urin	Anzahl der Patienten ≥ 70 Jahre mit Erstdiagnose CKD (Inzidenzindikator)	Ja
9	Anteil der Patienten ≥ 70 Jahre mit Erstdiagnose CKD (Inzidenzindikator), bei denen eine Urinuntersuchung auf Hämaturie mittels Streifentests erfolgte	RD **(5)**	CR	Anzahl der Patienten ≥ 70 Jahre mit Erstdiagnose CKD (Inzidenzindikator), die eine Urinuntersuchung mittels Streifentests erhalten haben	Anzahl der Patienten ≥ 70 Jahre mit Erstdiagnose CKD (Inzidenzindikator)	Ja
10	Anteil der Patienten ≥ 70 Jahre mit Erstdiagnose CKD (GFR < 60 ml/min/1,73 m^2^), bei denen in den letzten 12 Monaten (4 Quartale vor Indexquartal) keine CKD diagnostiziert/kodiert wurde, bei denen eine Kontrolle des Blutdrucks erfolgte	–	CR	Anzahl der Patienten ≥ 70 Jahre mit Erstdiagnose CKD (eGFR < 60 ml/min/1,73 m^2^, Inzidenzindikator), bei denen eine Blutdruckmessung in der Praxis durchgeführt wurde	Anzahl der Patienten ≥ 70 Jahre mit Erstdiagnose CKD (eGFR < 60 ml/min/1,73 m^2^, Inzidenzindikator)	Ja
11	Anteil der Patienten ≥ 70 Jahre mit CKD (eGFR < 60 ml/min/1,73 m^2^) unter 80 Jahren, deren systolischer Blutdruck auf ≤ 140 mm Hg eingestellt ist	–	CR **(2)**	Anzahl der Patienten ≥ 70 Jahre und < 80 Jahre mit CKD und mit einem systolischen Blutdruck ≤ 140 mm Hg	Anzahl der Patienten ≥ 70 Jahre und < 80 Jahre mit CKD	Ja
12	Anteil der Patienten ≥ 70 Jahre mit CKD und eGFR < 30 ml/min/1,73 m^2^, bei denen NSAR verordnet wurden	RD** (1)**	CR** (4)**	Anzahl der Patienten ≥ 70 Jahre mit CKD und eGFR < 30 ml/min/1,73 m^2^, die in den letzten 4 Quartalen NSAR verordnet bekommen haben	Anzahl der Patienten ≥ 70 Jahre mit CKD und eGFR < 30 ml/min/1,73 m^2^ in den letzten 4 Quartalen	Ja
13	Anteil der Patienten ≥70 Jahre mit CKD, deren eGFR entsprechend der Monitoringintervalle (abhängig vom eGFR- und Albuminuriewert) kontrolliert wurde^a^	–	CR **(6)**	Anzahl der Patienten ≥ 70 Jahre mit CKD, deren eGFR-Wert entsprechend der Monitoringintervalle kontrolliert wurde	Anzahl der Patienten ≥ 70 Jahre mit CKD	Ja
14	Anteil der Patienten ≥ 70 Jahre mit CKD, deren eGFR bei CKD-Stadium 1–3 oder unspezifisch kodiert mind. 1 × im Jahr und bei CKD-Stadium 4 mind. 2 × im Jahr kontrolliert wurde (unabhängig vom Albuminuriewert, der in Routinedaten nicht zur Verfügung steht)	RD	–	Anzahl der Patienten ≥ 70 Jahre mit CKD, deren eGFR-Wert entsprechend ihres CKD-Stadiums kontrolliert wurde	Anzahl der Patienten ≥ 70 Jahre mit CKD	Ja
15	Anteil der Patienten ≥ 70 Jahre mit CKD, bei denen mindestens einmal jährlich der Blutdruck dokumentiert wurde	–	CR **(1)**	Anzahl der Patienten ≥ 70 Jahre mit CKD, deren Blutdruck einmal jährlich gemessen wurde	Anzahl der Patienten ≥ 70 Jahre mit CKD	Ja
16	Anteil der Patienten ≥ 70 Jahre mit CKD und nachgewiesener Proteinurie, deren Albumin-Kreatinin-Ratio mindestens einmal jährlich gemessen wurde	RD	CR	Anzahl der Patienten ≥ 70 Jahre mit CKD und nachgewiesener Proteinurie, bei denen mindestens einmal jährlich die Kreatinin-Albumin-Ratio gemessen wurde	Anzahl der Patienten ≥ 70 Jahre mit CKD und nachgewiesener Proteinurie	Ja
17	Anteil der Patienten ≥ 70 Jahre mit CKD und ohne bekannte Anämie, bei denen einmal jährlich (CKD-Stadium 3) oder halbjährlich (CKD-Stadium 4) der Hämoglobinwert bestimmt wurde	RD **(6)**	CR	Anzahl der Patienten ≥ 70 Jahre mit CKD-Stadium 3 oder 4, bei denen 1 × jährlich (eGFR < 60 ml/min/1,73 m^2^) bzw. 2 × jährlich (eGFR < 30 ml/min/1,73 m^2^) der Hämoglobinwert im Blut bestimmt wurde	Anzahl der Patienten ≥ 70 Jahre mit CKD-Stadium 3 oder 4	Ja
18	Anteil der Patienten ≥ 70 Jahre mit CKD und erstmals festgestellter eGFR < 30 ml/min/1,73 m^2^, die an einen Nephrologen überwiesen wurden	RD **(3)**	CR	Anzahl der Patienten ≥ 70 Jahre mit CKD und erstmals festgestellter eGFR < 30 ml/min/1,73 m^2^ (keine eGFR < 30 ml/min in den letzten 4 Quartalen), die an einen Nephrologen überwiesen wurden	Anzahl der Patienten ≥ 70 Jahre mit CKD und erstmals festgestellter eGFR < 30 ml/min/1,73 m^2^	Nein
19	Anteil der Patienten ≥ 70 Jahre mit CKD und eGFR < 30 ml/min/1,73 m^2^ und Diabetes mellitus Typ 2, bei denen Metformin verordnet wurde	RD	CR **(7)**	Anzahl der Patienten ≥ 70 Jahre mit CKD und eGFR < 30 ml/min/1,73 m^2^ und Diabetes mellitus Typ 2, die Metformin verordnet bekommen haben	Anzahl der Patienten ≥ 70 Jahre mit CKD und eGFR < 30 ml/min/1,73 m^2^ und Diabetes mellitus Typ 2	Ja
20	Anteil der Patienten ≥ 70 Jahre mit CKD und Anämie (Hb < 6,8 mmol/l /< 11 mg/dl), bei denen zur Überprüfung des Eisenstatus der Serumferritinwert mit Serumtransferrinsättigung bestimmt wurde	–	CR	Anzahl der Patienten ≥ 70 Jahre mit CKD und Anämie (Hb < 6,8 mmol/l/< 11 mg/dl), bei denen der Serumferritinwert und die Serumtransferrinsättigung bestimmt wurde	Anzahl der Patienten ≥ 70 Jahre mit CKD und Anämie (Hb < 6,8 mmol/l/< 11 mg/dl)	Ja
21	Anteil der Patienten ≥ 70 Jahre mit CKD und Hypertonie, die im Rahmen der Therapie ACE-Hemmer *oder* ARB erhalten	RD	CR	Anzahl der Patienten ≥ 70 Jahre mit CKD und Hypertonie (ICD I10–I15), die ACE-Hemmer oder ARB verordnet bekommen haben	Anzahl der Patienten ≥ 70 Jahre mit CKD und Hypertonie (ICD I10–I15)	Ja
22	Anteil der Patienten ≥ 70 Jahre mit CKD, die in den letzten 12 Monaten eine Grippeschutzimpfung erhalten haben	RD **(7)**	CR	Anzahl der Patienten ≥ 70 Jahre mit CKD und Grippeschutzimpfung in den letzten 12 Monaten	Anzahl der Patienten ≥ 70 Jahre mit CKD	Ja
23	Anteil der Patienten ≥ 70 Jahre mit CKD, die über mindestens 2 Quartale mehrfach (≥ 2) gleichzeitig ACE-Hemmer und ARB innerhalb von 3 Quartalen nach dem Quartal mit der ersten Doppelverordnung verordnet bekommen	RD **(4)**	CR	Anzahl der Patienten ≥ 70 Jahre mit CKD und gleichzeitiger Verschreibung von ACE-Hemmern und ARB in mind. 2 Quartalen innerhalb von 3 Quartalen nach der ersten Doppelverordnung	Anzahl der Patienten ≥ 70 mit CKD im gleichen Beobachtungszeitraum wie Zähler	Ja

*ACE* Angiotensin-Converting-Enzym, *ARB* Angiotensin-Rezeptor-Blocker, *CR* Chart-Review, *E10* ICD-Kodierung für Diabetes mellitus, Typ 1, *E11* ICD-Kodierung für Diabetes mellitus, Typ 2, *eGFR* Estimated Glomerular Filtration Rate, *Hb* Hämoglobin, *ICD* International Statistical Classification of Diseases and Related Health Problems, *NSAR* nichtsteroidale Antirheumatika, *RD* Routinedaten

^a^Siehe S3-Leitlinie CKD ([1, S. 87], Abb. 2, adaptiert nach [6])

**Table 5:** 

Routinedaten	QI	Chart-Review	QI
1	QI 12 NSAR-Verordnung	1	QI 15 Blutdruckmessung mindestens jährlich
2	QI 8 ACR-Bestimmung bei Erstdiagnose	2	QI 11 Blutdruckeinstellung
3	QI 18 Überweisung Nephrologie bei fortgeschrittener CKD	3	QI 8 ACR-Bestimmung bei Erstdiagnose
4	QI 23 Gleichzeitige ACE-Hemmer- und ARB-Verordnung	4	QI 12 NSAR-Verordnung
5	QI 9 Streifentest auf Hämaturie bei Erstdiagnose	5	QI 3 Zwei eGFR-Bestimmungen zur Diagnosestellung
6	QI 17 Leitliniengerechte Hämoglobinkontrollen	6	QI 13 Leitliniengerechtes eGFR-Monitoring
(7^a^)	QI 22 Grippeschutzimpfung	(7^a^)	QI 19 Metforminverordnung

*ACE* Angiotensin-Converting-Enzym, *ACR* Albumin-Creatinin Ratio, *ARB* Angiotensin-Rezeptor-Blocker, *eGFR* Estimated Glomerular Filtration Rate, *NSAR* nichtsteroidale Antirheumatika

^a^Nr. 7 in jeder Liste stellt den zusätzlichen Indikator dar. Dieser kann im Falle der Nichtverfügbarkeit eines der ersten 6 QI eingesetzt werden

## Diskussion

### Zusammenfassung

Mittels Literaturrecherche und Operationalisierung von Empfehlungen der deutschen DEGAM-Leitlinie für Patient:innen mit chronischer Nierenerkrankung wurde eine Liste von 23 potenziellen Indikatoren erstellt. 17 der QI lassen sich mit Routinedaten und 21 der QI mit Chart-Review messen. Diese wurden durch 5 Expert:innen und einen Patient:innenvertreter in 2 Delphi-Fragerunden und abschließender Konsensuskonferenz evaluiert. Alle 23 vorgeschlagenen QI wurden von den Expert:innen als geeignet bewertet und wurden somit in den endgültigen QI-Katalog aufgenommen.

### Expert:innenkonsens

Die Befragung der Expert:innen ergab eine sehr hohe Übereinstimmung. Veränderungen wurden lediglich bei QI 9 bezüglich des Urinstreifentests auf Hämaturie sowie QI 22 bezüglich der Grippeschutzimpfung durchgeführt. Für QI 18, der sich auf die Überweisung an Nephrolog:innen bei erstmals festgestellter eGFR < 30 ml/min/1,73 m^2^ bezieht, wurde konsentiert, dass dieser nur bei nichtpalliativen Patient:innen gelten sollte, was im Beibehalten des originalen QI resultierte. Der Grund dafür war, dass auch bei Patient:innen mit geringer Lebenserwartung eine nephrologische Anbindung nicht prinzipiell ausgeschlossen werden sollte.

### Priorisierung der QI

Die Priorisierung der QI basierte auf Expert:innenannahmen über die derzeitige Versorgungsqualität von Patient:innen mit CKD sowie die Abbildbarkeit der QI mittels entsprechender Datenquellen (Routinedaten oder Chart-Review). Gebiete mit nach Expert:innenmeinung bestehendem Verbesserungsbedarf wurden entsprechend als wichtig eingestuft.

Von den Expert:innen hervorgehoben wurden die Vermeidung von nephrotoxischen Medikamenten bzw. Therapiekombinationen (QI 12, QI 19, QI 23), die regelmäßige Kontrolle und leitliniengerechte Einstellung des Blutdrucks (QI 15, QI 11), die nephrologische Anbindung bei fortgeschrittener CKD (QI 18), laborchemische Kontrollen auf Proteinurie, Hämaturie, Anämie und progredienten eGFR-Abfall (QI 8, QI 9, QI 17, QI 13), die leitliniengerechte Diagnosestellung durch eine zweimalige eGFR-Messung (QI 19) sowie eine Grippeschutzimpfung (QI 22).

#### Routinedaten

Als wichtigster durch Routinedaten abbildbarer QI identifizierten die Expert:innen die Verschreibung nichtsteroidaler Antirheumatika (NSAR) bei CKD-Patient:innen (QI 12). Die Einnahme von NSAR ist häufig [18], auch bei Patient:innen mit fortgeschrittener CKD [19]. Generell sollten NSAR bei Patient:innen mit chronischer Nierenerkrankung aufgrund des Risikos einer Nierenschädigung vermieden werden [20–22].

Angiotensin-Converting-Enzym(ACE)-Hemmer und Angiotensin-Rezeptor-Blocker (ARB) gehören zu den Medikamenten der ersten Wahl für die Behandlung von Patient:innen mit nichtdialysepflichtiger CKD mit häufigen Komorbiditäten wie Hypertonie und Albuminurie oder Diabetes mellitus und Albuminurie [5, 6, 23]. Diese werden bei CKD-Patient:innen im hausärztlichen Setting häufig eingesetzt [24]. Die gleichzeitige Gabe von ACE-Hemmern und ARB kann jedoch zu vermehrten Nebenwirkungen (Hyperkaliämie, Hypotonie, Nierenschädigung) führen und zeigt keinen positiven Effekt auf die Mortalität (QI 23; [25, 26]). Zwar sinkt inzwischen die Häufigkeit solcher Doppelverordnungen in Hausarztpraxen, sie tritt aber weiterhin auf [27].

Die Messung des Albumin-Kreatinin-Quotienten (Albumin-Creatinin Ratio – ACR) zur Erfassung einer Proteinurie bei Erstdiagnose einer CKD (QI 8) und die Anwendung von Harnstreifentests zur Diagnostik einer Hämaturie (QI 9) wurden von den Expert:innen als noch nicht ausreichend eingeschätzt. Tatsächlich werden ACR-Messungen im ambulanten Bereich nur selten durchgeführt, dabei stellt eine Proteinurie einen der wichtigsten prognostischen Faktoren einer Niereninsuffizienz dar [1, 28]. Ein Screening auf Hämaturie kann unter anderem ein nephritisches Syndrom als Ursache einer Niereninsuffizienz identifizieren. Laborchemische Kontrollen auf Proteinurie, Hämaturie sowie eine CKD-bedingte Anämie werden in Leitlinien zur Behandlung von Patient:innen mit CKD empfohlen (QI 17; [1, 5, 6]).

Im Falle einer Grippeinfektion erhöht die CKD das Risiko eines schweren Krankheitsverlaufs. Aufgrund der durch die Grippeschutzimpfung verursachten Senkung der Hospitalisierungs- und Mortalitätsrate bei Patient:innen mit fortgeschrittener CKD [29] wird eine Grippeschutzimpfung für CKD-Patient:innen in der KDIGO-Leitlinie empfohlen (QI 22) [5].

Eine nephrologische Mitbetreuung bei CKD kann unter anderem der Ursachenfindung, Therapieeinleitung sowie Evaluation und Behandlung von Komplikationen dienen [1]. Von KDIGO wird diese bei eGFR < 30 ml/min (QI 18) und/oder Progression der CKD empfohlen; weitere nephrologische Überweisungsanlässe wie Albuminurie, persistierende nichturologische Hämaturie und refraktäre Hypertonie werden durch andere Fachgesellschaften vorgeschlagen [1]. Eine nephrologische Konsiliaruntersuchung von Patient:innen mit einer eGFR < 30 ml/min/1,73 m^2^ (QI 18) kann die Lebenserwartung verlängern, indem potenziell behandelbare Ursachen einer Nierenfunktionsstörung erfasst und damit ihre Progression verlangsamt wird. Darüber hinaus kann die Notwendigkeit weiterer Maßnahmen wie Dialyse oder Transplantation professionell eingeschätzt werden [30]. Bei älteren, multimorbiden Patient:innen mit geringerer Lebenserwartung spiegelt der Einsatz dieses Indikators die Zusammenarbeit mit Nephrolog:innen möglicherweise nicht treffend wider. Insbesondere bei Palliativpatient:innen hat die Überweisung bei einer eGFR < 30 ml/min/1,73 m^2^ eine geringere klinische Konsequenz; in solchen Fällen ist eine Entscheidung gegen eine nephrologische Mitbetreuung häufiger möglich. Bei Patient:innen mit höherer Lebenserwartung ist dieser QI jedoch relevant, um einer weiteren Abnahme der Nierenfunktion möglichst vorzubeugen [30].

#### Chart-Review

Zusätzlich zu den ebenfalls durch Routinedaten abbildbaren QI zur Gabe von NSAR (QI 12) und der Bestimmung der ACR (QI 8) stellen jährliche Blutdruckmessungen (QI 15) und die Einstellung des systolischen Blutdrucks auf ≤ 140 mm Hg (QI 11) die wichtigsten durch Chart-Review abbildbaren QI dar. Hypertonie kann sowohl Ursache als auch Risiko für die Progression der CKD sein [31, 32]. Eine Blutdruckreduktion senkt das Risiko für Mortalität und kardiovaskuläre Ereignisse, auch bei CKD-Patient:innen [33]. Die Bestimmung der eGFR wird als wichtige Grundlage in der ambulanten Versorgung gesehen. Es werden jedoch keine großen Qualitätsdefizite erwartet, unter anderem weil diese Messungen im Rahmen des Disease-Management-(DMP‑)Programms für Diabetes mellitus im ambulanten Bereich regelmäßig durchgeführt werden [34]. Bei gleichzeitig bestehendem Diabetes mellitus und CKD wird wegen der Gefahr einer metabolischen Azidose (Laktatazidose) bei einer eGFR von < 30 ml/min/1,73 m^2^ gegen den Einsatz von Metformin in dieser Patient:innengruppe geraten [35].

### Einsetzbarkeit in Patient:innen unter 70 Jahren

Die Behandlung älterer Patient:innen mit CKD unterscheidet sich von der jüngerer Patient:innen [36]. QI 18 wurde als ungeeignet in der Anwendung bei Patient:innen unter 70 Jahren bewertet, weil nach Expert:innenmeinung die Überweisung jüngerer Patient:innen früher als bei einer eGFR < 30 ml/min/1,73 m^2^ erfolgen sollte. QI 18 stellt somit einen minimalen Standard für das Vorgehen in älteren Patient:innen mit CKD dar. Bei Anwendung dieses QI bei jüngeren Patient:innen befürchteten die Expert:innen eine zu späte Überweisung und damit eine verschlechterte Versorgungsqualität.

### Erfüllungsgrad

Der Erfüllungsgrad der QI wurde in der Konferenz nicht abgestimmt. Eine komplette Erfüllung oder Nichterfüllung ist aus verschiedenen Gründen nicht zu erwarten. Es ist möglich, dass im Gesamtbild die Erfüllung eines QI nicht von Nutzen wäre, beispielsweise die neue nephrologische Anbindung von Palliativpatient:innen mit einer eGFR unter 30 ml/min/1,73 m^2^. Zum anderen können QI von der Adhärenz oder Wahl der Patient:in abhängen. Hausärzt:innen können Patient:innen über die Vorteile einer Grippeschutzimpfung beraten und eine Grippeschutzimpfung empfehlen (QI 22). Die Entscheidung, diese wahrzunehmen, liegt letztendlich aber bei den Patient:innen. Für solche QI kann ein Erfüllungsgrad von 100 % weder erwartet werden noch zielführend sein. Das Hauptziel des Einsatzes der QI sollte eher sein, Bereiche des Verbesserungsbedarfs in der hausärztlichen Versorgung zu identifizieren. Erfüllungsgrade können in Folgestudien entwickelt und validiert werden.

### Stärken und Limitationen

Resultat unserer Studie ist die Entwicklung der ersten deutschen QI für die chronische nichtdialysepflichtige Nierenerkrankung. Die Evaluation der Liste der potenziellen QI erfolgte durch 5 Expert:innen und einen Patient:innenvertreter. Ähnlich wie in der Arbeit von Van den Bulck et al. [11] wurden durch Vertreter:innen der Nephrologie, Geriatrie und Allgemeinmedizin verschiedene Fachbereiche in den Bewertungsprozess der QI miteinbezogen. Durch einen Patient:innenvertreter wurde zudem die Sichtweise von Patient:innen mit chronischer Nierenerkrankung auf die Indikatoren widergespiegelt.

Die Anwendung eines QI in Routinedaten hängt von der Kodierqualität ab. Problematisch bei der Anwendung eines QI in Routinedaten ist die oft nicht ausreichende Verschlüsselung. Das Einschließen der unspezifischen ICD-Codes N18.8‑9 (sonstige chronische Nierenkrankheit bzw. nicht näher bezeichnet) erschwert die Anwendung von Indikatoren, die auf bestimmte Stadien begrenzt sind. Dennoch wurde der Code N18.9 für die Definition prävalenter und inzidenter CKD berücksichtigt, da er oft verwendet wird und sein Ausschluss zu einer möglichen Unterschätzung führen würde. Bei Einschluss von Indikatoren, die auf bestimmte Stadien begrenzt sind, kann es so aber auch zur Überschätzung der Versorgungsqualität kommen. Da NSAR nicht immer rezeptpflichtig sind, wird die Erfassung der eigentlichen Einnahme von NSAR von Patient:innen mit CKD erschwert, da rezeptfreie NSAR in Routinedaten nicht erfasst werden können. Chart-Reviews bieten zwar eine genauere Datenquelle, allerdings ist die Erhebung von Praxisdaten sowohl zeitlich als auch personaltechnisch aufwendig und daher nicht immer praktikabel. Die Voraussetzung für die Messung anhand eines Chart-Reviews ist eine ausreichende Dokumentation (Laborwerte, Messungen) in der Praxis.

Die Operationalisierung von Leitlinienempfehlungen basierte nur auf der deutschen S3-Leitlinie für chronische Nierenerkrankung [1]. Unsere QI basieren auf einer Metaleitlinie, die andere (internationale) Leitlinienempfehlungen berücksichtigt. Daher wurde die Operationalisierung von Leitlinienempfehlungen weiterer Leitlinien, wie in anderen Projekten teilweise durchgeführt [11, 16, 17], durch unsere Methodik nicht berücksichtigt. Es wurde keine systematische Literatursuche durchgeführt. Somit kann nicht garantiert werden, dass alle veröffentlichten QI berücksichtigt wurden.

## Fazit

Mittels eines interdisziplinären Delphi-Verfahrens wurden 23 QI für die ambulante Versorgung von Patient:innen mit nichtdialysepflichtiger CKD entwickelt. Dieses Projekt war das erste Modul des Konsortialprojektes „Leitliniengerechte Versorgung alter Patienten mit chronischer Nierenerkrankung (GUIDAGE-CKD)“. Im nächsten Schritt werden die hier identifizierten QI in einer Kombination aus Routinedaten der gesetzlichen Krankenversicherung AOK Nordost und Primärdaten einer epidemiologischen Altersstudie evaluiert. Nach Evaluierung in der Praxis kann mithilfe dieser Indikatoren zukünftig die Versorgungsqualität von Patient:innen mit chronischer Nierenerkrankung im ambulanten Bereich abgebildet werden. Hierdurch können Bereiche mit Verbesserungspotenzial aufgedeckt und Verbesserungsmöglichkeiten in der ambulanten Versorgung von Patient:innen mit CKD entwickelt werden.

## Supplementary Information






## References

[1] Weckmann G, Chenot J‑F, Stracke S (2019) Versorgung von Patienten mit chronischer nicht-dialysepflichtiger Nierenerkrankung in der Hausarztpraxis: S3 Leitlinie. https://www.awmf.org/leitlinien/detail/ll/053-048.html. Zugegriffen: 18. März 2022

[2] Szecsenyi J, Broge B, Stock J (2022) QISA: Das Qualitätsindikatorensystem für die ambulante Versorgung. In: AOK-Bundesverband GbR, AQUA-Institut für angewandte Qualitätsförderung und Forschung im Gesundheitswesen GmbH (Hrsg) (https://www.aok-gesundheitspartner.de/bund/qisa/ueber_qisa/index.html. Zugegriffen: 18 Mar 2022)

[3] Girndt M, Trocchi P, Scheidt-Nave C, Markau S, Stang A (2016). The prevalence of renal failure. Results from the German health interview and examination survey for adults, 2008–2011 (DEGS1). Dtsch Arztebl Int.

[4] Deckert S, Arnold K, Becker M (2021). Methodischer Standard für die Entwicklung von Qualitätsindikatoren im Rahmen von S3-Leitlinien – Ergebnisse einer strukturierten Konsensfindung. Z Evid Fortbild Qual Gesundhwes.

[5] Kidney Disease Improving Global Outcomes (2013) KDIGO 2012 clinical practice guideline for the evaluation and management of chronic kidney disease. https://kdigo.org/guidelines/. Zugegriffen: 14. Okt. 2022

[6] National Institute for Health and Care Excellence (2014) Chronic kidney disease in adults: assessment and management: clinical guideline. https://www.nice.org.uk/guidance/cg182. Zugegriffen: 20. Juni 2021 32208570

[7] Doran GT (1981). There’s a S.M.A.R.T. way to write management’s goals and objectives. Manage Rev.

[8] SoSci Survey GmbH SoSci Survey – die Lösung für eine professionelle Onlinebefragung. https://www.soscisurvey.de/. Zugegriffen: 27. Aug. 2022

[9] Reiter A, Fischer B, Kötting J, Geraedts M, Jäckel WH, Döbler K (2007). QUALIFY: Ein Instrument zur Bewertung von Qualitätsindikatoren. Z Arztl Fortbild Qualitatssich.

[10] Fitch K, Bernstein SJ, Aguilar MD (2001). The RAND/UCLA appropriateness method user’s manual.

[11] van den Bulck SA, Vankrunkelsven P, Goderis G (2020). Developing quality indicators for chronic kidney disease in primary care, extractable from the electronic medical record. A rand-modified Delphi method. BMC Nephrol.

[12] Kassenärztliche Bundesvereinigung (2010) Modifizierte RAND/UCLA-Methode: Anwendung im KBV-Projekt AQUIK: Ambulante Qualitätsindikatoren und Kennzahlen. https://www.kbv.de/media/sp/100521_Methodenpapier_AQUIK.pdf. Zugegriffen: 20. Juli 2022

[13] Tu K, Bevan L, Hunter K, Rogers J, Young J, Nesrallah G (2017). Quality indicators for the detection and management of chronic kidney disease in primary care in Canada derived from a modified Delphi panel approach. CMAJ Open.

[14] Bello AK, Ronksley PE, Tangri N (2019). Quality of chronic kidney disease management in Canadian primary care. JAMA Netw Open.

[15] Nash DM, Brimble S, Markle-Reid M (2017). Quality of care for patients with chronic kidney disease in the primary care setting: a retrospective cohort study from ontario, Canada. Can J Kidney Health Dis.

[16] Fukuma S, Shimizu S, Niihata K (2016). Development of quality indicators for care of chronic kidney disease in the primary care setting using electronic health data: a RAND-modified Delphi method. Clin Exp Nephrol.

[17] Litvin CB, Ornstein SM (2014). Quality indicators for primary care: an example for chronic kidney disease. J Ambul Care Manage.

[18] Sarganas G, Buttery AK, Zhuang W (2015). Prevalence, trends, patterns and associations of analgesic use in Germany. BMC Pharmacol Toxicol.

[19] Davison SN, Rathwell S, George C, Hussain ST, Grundy K, Dennett L (2020). Analgesic use in patients with advanced chronic kidney disease: a systematic review and meta-analysis. Can J Kidney Health Dis.

[20] Ungprasert P, Cheungpasitporn W, Crowson CS, Matteson EL (2015). Individual non-steroidal anti-inflammatory drugs and risk of acute kidney injury: A systematic review and meta-analysis of observational studies. Eur J Intern Med.

[21] Zhang X, Donnan PT, Bell S, Guthrie B (2017). Non-steroidal anti-inflammatory drug induced acute kidney injury in the community dwelling general population and people with chronic kidney disease: systematic review and meta-analysis. BMC Nephrol.

[22] Kuo H-W, Tsai S-S, Tiao M-M, Liu Y-C, Lee I-M, Yang C-Y (2010). Analgesic use and the risk for progression of chronic kidney disease. Pharmacoepidemiol Drug Saf.

[23] Bundesärztekammer (BÄK), Kassenärztliche Bundesvereinigung (KBV), Arbeitsgemeinschaft der Wissenschaftlichen Medizinischen Fachgesellschaften (AWMF) (2015) Nationale VersorgungsLeitlinie: Nierenerkrankungen bei Diabetes im Erwachsenenalter – Langfassung, 1. Aufl. Version 6. https://www.leitlinien.de/. Zugegriffen: 13. Okt. 2022

[24] Voigt P, Kairys P, Voigt A, Frese T (2021). Nicht dialysepflichtige, chronische Niereninsuffizienz in der Hausarztpraxis – eine Fragebogenstudie unter Hausärzten. Dtsch Med Wochenschr.

[25] Makani H, Bangalore S, Desouza KA, Shah A, Messerli FH (2013). Efficacy and safety of dual blockade of the renin-angiotensin system: meta-analysis of randomised trials. BMJ.

[26] Yusuf S, Teo KK, Pogue J, The ONTARGET Investigators (2008). Telmisartan, Ramipril, or both in patients at high risk for vascular events. N Engl J Med.

[27] Angelow A, Ploner T, Grimmsmann T, Walker J, Chenot J-F (2020). Dual renin-angiotensin-aldosterone blockade: Implementation of published research and Dear Doctor letters in ambulatory care: A retrospective observational study using prescription data. Pharmacoepidemiol Drug Saf.

[28] Kiel S, Weckmann G, Chenot J-F, Stracke S, Spallek J, Angelow A (2022). Referral criteria for chronic kidney disease: implications for disease management and healthcare expenditure-analysis of a population-based sample. BMC Nephrol.

[29] Gilbertson DT, Unruh M, McBean AM, Kausz AT, Snyder JJ, Collins AJ (2003). Influenza vaccine delivery and effectiveness in end-stage renal disease. Kidney Int.

[30] Liu P, Quinn RR, Karim ME (2019). Nephrology consultation and mortality in people with stage 4 chronic kidney disease: a population-based study. CMAJ.

[31] Palmer BF (2008). Hypertension management in patients with chronic kidney disease. Curr Hypertens Rep.

[32] Reynolds K, Gu D, Muntner P (2007). A population-based, prospective study of blood pressure and risk for end-stage renal disease in China. J Am Soc Nephrol.

[33] Ettehad D, Emdin CA, Kiran A (2016). Blood pressure lowering for prevention of cardiovascular disease and death: a systematic review and meta-analysis. Lancet.

[34] Kassenärztliche Bundesvereinigung (KBV) (2020) Disease-Management-Programm Diabetes Mellitus Typ 2 – Qualitätszielerreichung 2020: Quelle: Indikationsspezifische Berichte für die Gemeinsamen Einrichtungen bzw. Qualitätsberichte aus 16 Kassenärztlichen Vereinigungen. https://www.kbv.de/media/sp/DMP_Diabetes2_Ergebnisse_QS.pdf. Zugegriffen: 13. Okt. 2022

[35] Lazarus B, Wu A, Shin J-I (2018). Association of Metformin use with risk of lactic acidosis across the range of kidney function: a community-based cohort study. JAMA Intern Med.

[36] Farrington K, Covic A, Nistor I (2017). Clinical Practice Guideline on management of older patients with chronic kidney disease stage 3b or higher (eGFR 45 mL/min/1.73 m2): a summary document from the European Renal Best Practice Group. Nephrol Dial Transplant.

