# Supplementary material for: Development of quality indicators for the care of patients with chronic kidney disease: Results of a structured consensus process using the Delphi technique
Source: Bundesgesundheitsblatt Gesundheitsforschung Gesundheitsschutz. 2023 May 16;66(6):677–88. [Article in German] doi: 10.1007/s00103-023-03700-9 (PMC10261173; doi:10.1007/s00103-023-03700-9)
Supplement: Supplementary file 1 [file 103_2023_3700_MOESM1_ESM.pdf]

Zusätzliches Onlinematerial

Onlinematerial 1. Sosci-Survey Befragung 2. Delphi-Runde, Quelle: [1]

Universitätsmedizin GREIFSWALD

Qualitätsindikatoren für die Versorgung von Erwachsenen mit nicht-dialysepflichtiger chronischer Nierenerkrankung

Bitte geben Sie hier Ihren Namen an.

Entwicklung von Qualitätsindikatoren zur leitliniengerechten Versorgung von Patienten ab 70 Jahre mit chronischer nicht-dialysepflichtiger Nierenerkrankung – Delphi Runde 2

Vorwort

Die vorgeschlagenen Qualitätsindikatoren sind aus Empfehlungen der S3-Leitlinie "Versorgung von Patienten mit chronischer nicht-dialysepflichtiger Nierenerkrankung in der Hausarztpraxis" abgeleitet worden, sowie von bereits vorgeschlagenen Qualitätsindikatoren basierend auf internationalen Leitlinien. Diese Qualitätsindikatoren sind für die ambulante Versorgung von Erwachsenen mit eingeschränkter, nicht-dialysepflichtiger chronischer Nierenerkrankung in Hausarztpraxen entwickelt worden.

Nicht Gegenstand dieser Qualitätsindikatoren ist die Versorgung von:

- Kindern bis zum 18. Lebensjahr
- Patienten mit akuter Nierenschädigung (AKI - Acute Kidney Injury)
- Frauen während der Schwangerschaft, einschließlich Diabetes in der Schwangerschaft
- Patienten die bereits dialysepflichtig sind (ICD-Code: N18.5 - dialysepflichtige chronische Nierenerkrankung)
- Patienten nach Nierentransplantation
- stationäre Patienten
- Patienten mit einer eGFR ≥ 60 ml/min und einer isolierten Hämaturie
- Patienten mit einer eGFR ≥ 60 ml/min und einer isolierten Proteinurie/Albuminurie
- Patienten mit genetischen Erkrankungen oder Syndromen, wie z.B. Alport-Syndrom, Morbus Fabry, genetisch bedingten Zystennieren

Nachfolgend wird nur noch von chronischer Nierenerkrankung gesprochen, wobei die dialysepflichtigen chronischen Nierenerkrankungen nicht inbegriffen sind.

Eine neu diagnostizierte chronische Nierenerkrankung (CKD, Erstdiagnose) wird definiert als:

$$Inzidenz = \frac{Anzahl\ \geq 70\ jährige\ Patienten\ mit\ CKD, ohne\ CKD\ in\ den\ letzten\ 4\ Quartalen\ vor\ Indexquartal}{Alle\ Patienten\ \geq 70\ Jahre\ ohne\ CKD\ in\ den\ letzten\ 4\ Quartalen\ vor\ Indexquartal}$$

Das Indexquartal bezeichnet das Quartal, in dem die diagnostizierte/verschlüsselte CKD zum ersten Mal auftritt.

Eine bestehende (prävalente) CKD wird definiert als:

$$Prävalenz = \frac{Anzahl\ \geq 70\ jährige\ Patienten\ mit\ diagnostizierter\ oder\ kodierter\ nicht\ dialysepflichtiger\ CKD}{Alle\ Patienten\ \geq 70\ Jahre}$$

Die Datenbasis für die Messung sind entweder Routinedaten (Abrechnungsdaten) oder /und das Chart Review (Medical Record Review oder auch Audit - Überprüfung der Krankenakte von Patienten). Soweit es inhaltlich möglich war, wurden Routinedaten bevorzugt ausgewählt. Sind sowohl Routinedaten als auch Chart Review ausgewählt, soll die Praktikabilität anhand von Routinedaten bewertet werden.

Der Betrachtungszeitraum bezieht sich im Regelfall auf 4 Quartale (12 Monate), es sei denn, es ist anders spezifiziert. Für Dauerdiagnosen muss die Verfügbarkeit von älteren Diagnosen (über die 4 Quartale hinaus) sichergestellt und berücksichtigt werden, da die Prävalenz sonst unterschätzt wird. Dauerdiagnosen werden vom Hausarzt nicht immer in jedem Quartal fortlaufend verschlüsselt.

Zu berücksichtigen ist, dass bei Nutzung von Routinedaten nur Patienten eingeschlossen werden können, die im Betrachtungszeitraum (i.d.R. 4 Quartale) durchgängig versichert waren bzw. durchgängig bei der gleichen Krankenkasse versichert waren und nicht verstorben sind. Bei der Durchführung eines Chart Reviews ist zu berücksichtigen, dass die Patienten im Betrachtungszeitraum bei demselben Arzt in Behandlung waren (z. B. Ausschluss von Patienten, die gerade neu zu dem Arzt gewechselt sind) und ebenfalls nicht verstorben sind. Dies ist jedoch nur eingeschränkt möglich (Ärztehopping). Bei einem Chart Review wird lediglich eine Stichprobe der betreffenden Patienten (am besten eine Zufallsstichprobe) in den Hausarztpraxen einbezogen.

Der ICD-10 Code N18.9 - chronische Nierenkrankheit, nicht näher bezeichnet - wird grundsätzlich in die Berechnungen mit aufgenommen, da er oft für die Verschlüsselung von CKD verwendet wird und bei Ausschluss zur Unterschätzung führen würde. Patienten, die dialysepflichtig sind, sind mit sehr großer Wahrscheinlichkeit korrekt (durch den Nephrologen/die Nephrologin) verschlüsselt. Daher gehen wir davon aus, dass unter N18.9 keine dialysepflichtigen Patienten kodiert sind. Als Sensitivitätskontrolle können zusätzlich die ICD-10 Codes für Dialysebehandlung (Z49; Z99.2) und die OPS-Codes für Hämofiltration (8-853), Hämodialyse (8-854), Hämodiafiltration (8-855), Peritonealdialyse (8-857) und Nierentransplantation (5-555) berücksichtigt werden.

Der Prävalenz- sowie der Inzidenzindikator stehen nicht zur Abstimmung, da sie oft Teil anderer Qualitätsindikatoren sind. Es sind lediglich Verbesserungsvorschläge zur Messung von Inzidenz und Prävalenz vorgesehen.

Die Beschreibung der einzelnen Indikatoren orientiert sich an der folgenden Struktur:

Allgemeiner Aufbau:

Nr.	vorgeschlagener Qualitätsindikator
Fokus: Screening, Diagnose, Medikamentöse Therapie, Monitoring	
Grundlage: Leitlinienempfehlung, bereits vorgeschlagene QI's, abgeleitet aus Literatur	
☐ Routinedaten ☐ Chart Review	
Datenquelle:<br>· Primärdaten (Chart Review)<br>· Sekundärdaten (Routinedaten)	
Zähler:	
Nenner:	
Hintergrund:	
Problem:	
Dieser QI wird auch vorgeschlagen von:	

1. Prävalenzindikator (etablierte CKD):

Anteil der Patienten ≥ 70 Jahren, bei denen eine chronische nicht-dialysepflichtige Nierenerkrankung diagnostiziert wurde.

Fokus: Screening

☒ Routinedaten ☒ Chartreview

Routinedaten:

- ICD-10-Code: N18
- Stadium 1 - eGFR ≥ 90 ml/min/1,73 m^2 – N18.1
- Stadium 2 - eGFR ≥ 60 - < 90 ml/min/ 1,73 m^2 – N18.2
- Stadium 3 - eGFR ≥ 30 - < 60 ml/min/ 1,73 m^2 – N18.3
- Stadium 4 - eGFR ≥ 15 - < 30 ml/min/1,73 m^2 – N18.4
- Chronische Nierenkrankheit, nicht näher bezeichnet - N18.9

Zähler: Anzahl der ≥70-jährigen Patienten mit diagnostizierter bzw. kodierter **und** nicht dialysepflichtiger CKD

Nenner: Alle Patienten ≥70

Hintergrund: Die Chronische Nierenerkrankung (CKD) ist definiert über die Nierenfunktion (mit oder ohne Nierenschaden). Die Einteilung in Schweregrade erfolgt über die eGFR (Nierenfunktion) und Albuminurie (Nierenschaden) (KDIGO). Der Qualitätsindikator liefert einen Überblick über den Anteil der mit CKD diagnostizierten Patienten in der Praxis und eine Grundlage zur Prävalenzschätzung der Erkrankung.

Problem: Insbesondere die frühen Stadien (eGFR > 60 ml/min/1,73m^2) werden oft nicht ausreichend kodiert. Diese Stadien bleiben häufig auch asymptomatisch und werden deshalb in der Praxis nicht erkannt. Zudem ist eine Fehlkodierung möglich oder die Kodierung N18.9 (CKD – nicht näher bezeichnet), welche eine Einordnung in die Stadien nicht erlaubt. Durch die strikten Grenzwerte der unterschiedlichen Stadien kann außerdem durch verschiedene Messungen eine unterschiedliche Einordnung in die Stadien erfolgen. In der Praxis ist die Überprüfung der eGFR als Grundlage für die Diagnose CKD nicht immer gegeben. In der Praxis wird häufig keine Albumin-Kreatinin-Ratio (ACR) bestimmt. Bestimmte Behandlungsempfehlungen sind an ein CKD Stadium gebunden. In diesen Fällen wird der entsprechende ICD Code verwendet. Bei Verschlüsselung ICD N18.5 (eGFR <15 ml/min) wird angenommen, dass diese Patienten dialysepflichtig sind, auch wenn dies nicht immer der Fall ist. Diese werden daher beim Prävalenzindikator nicht berücksichtigt. Alternativ kann die N18.5 bei Patienten ohne folgende ICD-Codes Dialysebehandlung (Z49; Z99.2) und die OPS-Codes für Hämofiltration (8-853), Hämodialyse (8-854), Hämodiafiltration (8-855), Peritonealdialyse (8-857) und Nierentransplantation (5-555) mitberücksichtigt werden.

Dieser QI wird auch vorgeschlagen von:

- NICE QOF: The contractor establishes and maintains a register of patients aged 18 years or over with CKD with classification of categories G3a to G5 (previously stage 3 to 5) (NICE ID code: NM83)
- ICES: The primary care providers can identify patients in their practice aged 18 years and older with chronic kidney disease.

Dieser Qualitätsindikator steht nicht zur Abstimmung. Bitte geben Sie nur Verbesserungsvorschläge für die Definition ab.

Universitätsmedizin GREIFSWALD

2. Inzidenzindikator (erstmals diagnostizierte CKD):

Anteil der Patienten ≥70 Jahre, bei denen eine chronische, nicht-dialysepflichtige Nierenerkrankung neu diagnostiziert wurde.

Fokus: Screening

☒ Routinedaten ☒ Chartreview

Routinedaten:

ICD-10-Code:

- eGFR ≥ 90 ml/min – Stadium 1 – N18.1
- eGRF ≥ 60 - < 90 ml/min – Stadium 2 – N18.2
- eGFR ≥ 30 - < 60 ml/min – Stadium 3 – N18.3
- eGFR ≥ 15 - < 30 ml/min – Stadium 4 – N18.4
- chronische Nierenkrankheit, nicht näher bezeichnet – N18.9

Zähler: Anzahl der Patienten ≥70 Jahre mit neu diagnostizierter bzw. kodierter CKD, bei denen in den letzten 12 Monaten (4 Abrechnungsquartale vor Indexquartal) keine CKD diagnostiziert / kodiert wurde.

Nenner: Alle Patienten ≥70 Jahre, bei denen in den letzten 12 Monaten (4 Abrechnungsquartale vor Indexquartal) keine CKD diagnostiziert / kodiert wurde.

Hintergrund: Die Erstdiagnose wird definiert als erstmalige Abrechnung CKD und mindestens 4 Quartale vorher keine Verschlüsselung der CKD.

Problem: Eine sichere Abgrenzung zu AKI (Acute Kidney Injury) ist nicht immer möglich.

Dieser Qualitätsindikator steht nicht zur Abstimmung. Bitte geben Sie nur Verbesserungsvorschläge für die Definition ab.

Universitätsmedizin GREIFSWALD

3. Anteil der Patienten ≥70 Jahre mit erstmals festgestellter eGFR <60 ml/min, bei denen für die Erstdiagnose eine zweimalige Messung der eGFR im Abstand von 3 Monaten durchgeführt wurde.

Fokus: Diagnose

Grundlage: „Bei Erstdiagnose einer GFR < 60 ml/min, soll eine weitere Bestimmung der geschätzten glomerulären Filtrationsrate (eGFR) nach 3 Monaten durchgeführt werden, um die Diagnose einer CKD zu stellen" (DEGAM-Empfehlung, 6.1, basierend auf KDIGO)

☐ Routinedaten ☒ Chart Review

Chart Review

Patienten mit eGFR <60 ml/min und zweimaliger Messung der eGFR im Abstand von 3 Monaten

Zähler: Anzahl der Patienten ≥70 Jahre mit erstmals festgestellter eGFR <60 ml/min, bei denen im Diagnosequartal zwei eGFR-Bestimmungen durchgeführt wurden oder eine weitere Messung im Folgequartal.

Nenner: Anzahl der Patienten ≥70 Jahre mit erstmals festgestellter eGFR <60 ml/min.

Hintergrund: Die Chronische Nierenerkrankung ist definiert als Abnormalität der Nierenfunktion oder Nierenstruktur über 3 Monate (KDIGO). Für die Diagnose sollte eine zweite eGFR-Messung 3 Monate nach der erstmals festgestellten erniedrigten eGFR erfolgen.

Problem: In der Praxis wird aus praktischen Gründen häufig schon bei erstmals erniedrigter eGFR die CKD-Diagnose gestellt, da eine AKI (Acute Kidney Injurie) selten ist und die Einschätzung anhand der Komorbiditäten es oft erlaubt, sich ausreichend sicher festzulegen.

Dieser QI wird auch vorgeschlagen von:

- ICES: Percentage of patients with an initial estimated glomerular filtration rate (eGFR) < 60 mL/min per 1.73 m^2 that is followed by a repeat test within 6 months.

Anteil der Patienten ≥70 Jahre mit erstmals festgestellter eGFR <60 ml/min, bei denen für die Erstdiagnose eine zweimalige Messung der eGFR im Abstand von 3 Monaten durchgeführt wurde.

	stimme nicht zu 1	2	3	4	stimme teilweise zu 5	6	7	8	stimme voll zu 9	Enthaltung
Relevanz	○	○	○	○	○	○	○	○	○	○
Wissenschaftlichkeit	○	○	○	○	○	○	○	○	○	○
Praktikabilität	○	○	○	○	○	○	○	○	○	○

Bitte begründen Sie Ihre Entscheidung und geben Sie Änderungsvorschläge, wenn Sie sich für Enthaltung oder einen Skalenwert von 1-3 entschieden haben.

4. Anteil der Patienten ≥70 Jahre mit neu diagnostizierter CKD, bei denen für die Erstdiagnose eine zweimalige Messung der eGFR im Abstand von 3 Monaten durchgeführt wurde.

Fokus: Diagnose

Grundlage: „Bei Erstdiagnose einer GFR < 60 ml/min, soll eine weitere Bestimmung der geschätzten glomerulären Filtrationsrate (eGFR) nach 3 Monaten durchgeführt werden, um die Diagnose einer CKD zu stellen" (DEGAM-Empfehlung, 6.1, basierend auf KDIGO)

☒ Routinedaten ☐ Chart Review

Routinedaten

- ICD-Nummern CKD: N18.1, N18.2, N18.3, N18.4, N18.9
- Abrechnung eGFR und Serumkreatininbestimmung: GOP 32066, 32067

Zähler: Anzahl der Patienten ≥70 Jahre mit Erstdiagnose CKD (Inzidenzindikator), bei denen im Diagnosequartal zwei eGFR-Bestimmungen durchgeführt wurden oder eine weitere Messung im Vorquartal.

Nenner: Anzahl der Patienten ≥70 Jahre mit Erstdiagnose CKD (Inzidenzindikator).

Hintergrund: Die Chronische Nierenerkrankung ist definiert als Abnormalität der Nierenfunktion oder Nierenstruktur über 3 Monate (KDIGO). Für die Diagnose sollte eine zweite eGFR-Messung 3 Monate nach der erstmals festgestellten erniedrigten eGFR erfolgen.Die Abrechnungsziffer für die Kreatinin-Messung gilt als Bestimmung der eGFR, die von Laboren meist automatisch mit angegeben wird.

Problem:In Routinedaten ist die Erstdiagnose eGFR <60 ml/min nicht abbildbar, sondern nur die CKD verschlüsselt. Daher wird hier im Vorquartal nach der zweiten eGFR Bestimmung geschaut. In der Praxis wird aus praktischen Gründen häufig schon bei erstmals erniedrigter eGFR die CKD-Diagnose gestellt, da eine AKI selten ist und die Einschätzung anhand der Komorbiditäten es oft erlaubt, sich ausreichend sicher festzulegen. Aus den Routinedaten ist zudem lediglich die Abrechnung der eGFR-Messung (GOP) ersichtlich, die Überprüfung der gemessenen Laborwerte zu den beiden verschiedenen Zeitpunkten ist jedoch mit Routinedaten nicht möglich.

Dieser QI wird auch vorgeschlagen von:

- ICES: Percentage of patients with an initial estimated glomerular filtration rate (eGFR) < 60 mL/min per 1.73 m^2 that is followed by a repeat test within 6 months.

Anteil der Patienten ≥70 Jahre mit neu diagnostizierter CKD, bei denen für die Erstdiagnose eine zweimalige Messung der eGFR im Abstand von 3 Monaten durchgeführt wurde.

	1 (stimme nicht zu)	2	3	4	5 (stimme teilweise zu)	6	7	8	9 (stimme voll zu)	Enthaltung
Relevanz	○	○	○	○	○	○	○	○	○	○
Wissenschaftlichkeit	○	○	○	○	○	○	○	○	○	○
Praktikabilität	○	○	○	○	○	○	○	○	○	○

Bitte begründen Sie Ihre Entscheidung und geben Sie Änderungsvorschläge, wenn Sie sich für Enthaltung oder einen Skalenwert von 1-3 entschieden haben.

5. Anteil der Patienten ≥70 Jahre mit Diabetes mellitus, bei denen mindestens einmal jährlich die eGFR bestimmt wurde.

Fokus: Screening

Grundlage: „Zur Erfassung der Nierenfunktion bei Patienten ≥70 mit Diabetes mellitus sollte einmal jährlich die eGFR bestimmt werden" (DEGAM-Empfehlung, 5.2)

☒ Routinedaten ☒ Chartreview

Routinedaten:

- Diabetes: ICD: E11.- (DM Typ 2), E10.- (DM Typ 1)
- Kreatininbestimmung im Serum: GOP 32066, 32067
- Laborausnahmeziffer (manifester Diabetes mellitus): 32022 (Glucose, Kreatinin, HbA1C, Mikroalbumin im Urin)

Zähler: Anzahl der Patienten ≥70 Jahre mit Diabetes mellitus (ICD E11, E10), bei denen mindestens einmal innerhalb eines Jahres eine eGFR-Bestimmung erfolgte.

Nenner: Anzahl der Patienten ≥70 Jahre mit Diabetes mellitus (ICD E11, E10)

Hintergrund: Einer der wichtigsten Ursachen der CKD ist Diabetes mellitus. Deshalb sollte mindestens einmal jährlich die eGFR kontrolliert werden, um das mögliche Vorliegen einer CKD zu überprüfen. (NVL Diabetes, DMP Diabetes, DEGAM-Leitlinie)

Dieser QI wird auch vorgeschlagen von:

- ICES: Percentage of patients with risk factors for chronic kidney disease (diabetes, hypertension) with an eGFR in the past 18 months.

Anteil der Patienten ≥70 Jahre mit Diabetes mellitus, bei denen mindestens einmal jährlich die eGFR bestimmt wurde.

	1 stimme nicht zu	2	3	4	5 stimme teilweise zu	6	7	8	9 stimme voll zu	Enthaltung
Relevanz	○	○	○	○	○	○	○	○	○	○
Wissenschaftlichkeit	○	○	○	○	○	○	○	○	○	○
Praktikabilität (bei Verwendung von Routinedaten)	○	○	○	○	○	○	○	○	○	○

Bitte begründen Sie Ihre Entscheidung und geben Sie Änderungsvorschläge, wenn Sie sich für Enthaltung oder einen Skalenwert von 1-3 entschieden haben.

6. Anteil der Patienten ≥70 Jahre mit Erstdiagnose Bluthochdruck und ohne bekannte CKD, die eine Bestimmung der eGFR erhalten haben.

Fokus: Screening

Grundlage: Patienten ≥70 ohne bekannte CKD, soll bei Erstdiagnose eines Bluthochdrucks eine Serumkreatinin-Bestimmung mit eGFR und eine Urinuntersuchung auf Eiweiß oder Albumin angeboten werden. (DEGAM-Leitlinie, 5.3)

☒ Routinedaten ☒ Chartreview

Routinedaten:

- Erstmals kodierter Bluthochdruck (ICD I10-I15) definiert als jeweils eine Kodierung in 2 unterschiedlichen Quartalen innerhalb von 1 Jahr (4 Quartalen) und mindestens 4 Quartale vor Indexquartal keine Verschlüsselung
- Kreatinin mittels Jaffe: 32066, enzymatisch: 32067

Zähler: Anzahl der Patienten ≥70 Jahre mit Erstdiagnose Bluthochdruck (ICD I10-I15, kodiert in 2 unterschiedlichen Quartalen innerhalb von 1 Jahr und mind. 4 Quartale vor Indexquartal keine Verschlüsselung), die eine Serumkreatinin-Bestimmung mit eGFR erhalten haben.

Nenner: Anzahl der Patienten ≥70 Jahre mit Erstdiagnose Bluthochdruck (ICD I10-I15, kodiert in 2 unterschiedlichen Quartalen innerhalb von 1 Jahr und mind. 4 Quartale vor Indexquartal keine Verschlüsselung)

Hintergrund: Der Bluthochdruck ist einer der wichtigsten Ursachen für die Entstehung der CKD. Patienten mit Hypertonie haben ein erhöhtes Risiko, an einer CKD zu erkranken. Das absolute Risiko, an einer terminalen Nierenerkrankung zu erkranken, ist jedoch gering (DEGAM-Leitlinie). Die DEGAM-Leitlinie empfiehlt bei Erstdiagnose eine Überprüfung der Nierenfunktion, angesichts der Häufigkeit der Hypertonie in der Bevölkerung aber keine routinemäßige Untersuchung, wenn eine Nierenerkrankung initial ausgeschlossen wurde.

Dieser QI wird auch vorgeschlagen von:

- ICES: Percentage of patients with risk factors for chronic kidney disease (diabetes, hypertension) with an eGFR in the past 18 months.

Anteil der Patienten ≥70 Jahre mit Erstdiagnose Bluthochdruck und ohne bekannte CKD, die eine Bestimmung der eGFR erhalten haben.

	1 (stimme nicht zu)	2	3	4	5 (stimme teilweise zu)	6	7	8	9 (stimme voll zu)	Enthaltung
Relevanz	○	○	○	○	○	○	○	○	○	○
Wissenschaftlichkeit	○	○	○	○	○	○	○	○	○	○
Praktikabilität (bei Verwendung von Routinedaten)	○	○	○	○	○	○	○	○	○	○

Bitte begründen Sie Ihre Entscheidung und geben Sie Änderungsvorschläge, wenn Sie sich für Enthaltung oder einen Skalenwert von 1-3 entschieden haben.

Universitätsmedizin GREIFSWALD

7. Anteil der Patienten ≥70 Jahre mit Erstdiagnose Bluthochdruck und ohne bekannte CKD, die eine Urinuntersuchung auf Protein oder Albumin erhalten haben.

Fokus: Screening

Grundlage: Patienten ohne bekannte CKD, soll bei Erstdiagnose eines Bluthochdrucks eine Serumkreatinin-Bestimmung mit eGFR und eine Urinuntersuchung auf Eiweiß oder Albumin angeboten werden. (DEGAM-Leitlinie, 5.3)

☒ Routinedaten ☒ Chartreview

Routinedaten:

- Erstmals kodierter Bluthochdruck (ICD I10-I15) definiert als jeweils eine Kodierung in 2 unterschiedlichen Quartalen innerhalb von 1 Jahr (4 Quartalen) und mindestens 4 Quartale vor Indexquartal keine Verschlüsselung
- GOP 32435 Albuminbestimmung, Kreatinin mittels Jaffe: 32066 oder enzymatisch: 32067

Zähler: Anzahl der Patienten ≥70 Jahre mit Erstdiagnose Bluthochdruck (ICD I10-I15, kodiert in 2 unterschiedlichen Quartalen innerhalb von 1 Jahr und mind. 4 Quartale vor Indexquartal keine Verschlüsselung) die eine Urinuntersuchung auf Eiweiß oder Albumin erhalten haben.

Nenner: Anzahl der Patienten ≥70 Jahre mit Erstdiagnose Bluthochdruck (ICD I10-I15, kodiert in 2 unterschiedlichen Quartalen innerhalb von 1 Jahr und mind. 4 Quartale vor Indexquartal keine Verschlüsselung).

Hintergrund: Der Bluthochdruck ist einer der wichtigsten Ursachen für die Entstehung der CKD. Patienten mit Hypertonie haben ein erhöhtes Risiko, an einer CKD zu erkranken. Das absolute Risiko, an einer terminalen Nierenerkrankung zu erkranken, ist jedoch gering. (DEGAM-Leitlinie) Die DEGAM-Leitlinie empfiehlt bei Erstdiagnose eine Überprüfung der Nierenfunktion, angesichts der Häufigkeit der Hypertonie in der Bevölkerung aber keine routinemäßige Untersuchung, wenn eine Nierenerkrankung initial ausgeschlossen wurde.

Anteil der Patienten ≥70 Jahre mit Erstdiagnose Bluthochdruck und ohne bekannte CKD, die eine Urinuntersuchung auf Protein oder Albumin erhalten haben.

	stimme nicht zu				stimme teilweise zu				stimme voll zu	Enthaltung
	1	2	3	4	5	6	7	8	9	
Relevanz	○	○	○	○	○	○	○	○	○	○
Wissenschaftlichkeit	○	○	○	○	○	○	○	○	○	○
Praktikabilität (bei Verwendung von Routinedaten)	○	○	○	○	○	○	○	○	○	○

Bitte begründen Sie Ihre Entscheidung und geben Sie Änderungsvorschläge, wenn Sie sich für Enthaltung oder einen Skalenwert von 1-3 entschieden haben.

Universitätsmedizin GREIFSWALD

8. Anteil der Patienten ≥70 mit Erstdiagnose CKD (Inzidenzindikator), bei denen eine Bestimmung der Albumin-Kreatinin-Ratio im Urin erfolgte.

Fokus: Diagnose

Grundlage: „Bei Erstdiagnose der CKD (GFR < 60 ml/min) soll eine Bestimmung der Albumin-Kreatinin-Ratio im Urin erfolgen" (DEGAM-Empfehlung, 6.3)

☒ Routinedaten ☒ Chartreview

Routinedaten:
Chronische Nierenerkrankung

- ICD: N18.1, N18.2, N18.3, N18.4, N18.9 (nicht näher klassifiziert)

Bestimmung der ACR mittels Spontanurinprobe (spot urine) oder 24-h-Sammelurin

- Kreatinin GOP 32066, 32067 und Albumin GOP 32435 (beides muss gleichzeitig abgerechnet worden sein.)

Zähler: Anzahl der Patienten ≥70 Jahre mit Erstdiagnose CKD (Inzidenzindikator) und mindestens einer Bestimmung der Albumin-Kreatinin-Ratio im Urin

Nenner: Anzahl der Patienten ≥70 Jahre mit Erstdiagnose CKD (Inzidenzindikator)

Hintergrund: Die Proteinurie ist ein weiterer wichtiger prognostischer Marker für die chronische Nierenerkrankung. Die Albumin-Kreatinin-Ratio (ACR) wird mit aus dem Urin gemessenen Kreatinin- und Albuminwerten bestimmt. Mittels ACR kann die CKD neben den Stadien in ein Stadium A1 (< 30 mg/g), A2 (30-300 mg/g) und A3 (>300 mg/g) eingeteilt werden. Zur Bestimmung der Werte wird vorwiegend Spontanurin benutzt, welcher fehleranfällig sein kann. (DEGAM-Leitlinie). Abweichend von der DEGAM Empfehlung wird hier auch CKD Stadium 1 und 2 mitberücksichtigt. Auch bei der Diagnose einer CKD bei eGFR >60 ml/min ist eine Messung der Proteinurie zur Beurteilung der Nierenfunktion notwendig.

Problem: Kreatinin und Albumin müssen zur gleichen Zeit abgerechnet worden sein. Das Albumin-Kreatinin-Ratio wird durch das Labor nur dann berechnet, wenn beide Parameter abgerechnet worden sind.

Dieser QI wird auch vorgeschlagen von:

- ICES: Percentage of patients with an initial eGFR < 60 mL/min/1.73 m^2 with an albumin–creatinine ratio (ACR) test conducted within 6 months.

Anteil der Patienten ≥70 Jahre mit Erstdiagnose CKD (Inzidenzindikator), bei denen eine Bestimmung der Albumin-Kreatinin-Ratio im Urin erfolgte.

	1 (stimme nicht zu)	2	3	4	5 (stimme teilweise zu)	6	7	8	9 (stimme voll zu)	Enthaltung
Relevanz	○	○	○	○	○	○	○	○	○	○
Wissenschaftlichkeit	○	○	○	○	○	○	○	○	○	○
Praktikabilität (bei Verwendung von Routinedaten)	○	○	○	○	○	○	○	○	○	○

Bitte begründen Sie Ihre Entscheidung und geben Sie Änderungsvorschläge, wenn Sie sich für Enthaltung oder einen Skalenwert von 1-3 entschieden haben.

Universitätsmedizin GREIFSWALD

9. Anteil der Patienten ≥70 Jahre mit Erstdiagnose CKD (Inzidenzindikator), bei denen eine Urinuntersuchung mittels Streifentest erfolgte.

Fokus: Diagnose

Grundlage: „Bei Erstdiagnose der CKD (GFR < 60 ml/min) soll eine Bestimmung der Hämaturie mit einem Streifentest im Urin erfolgen." (DEGAM-Empfehlung, 6.4)

☒ Routinedaten ☒ Chartreview

Routinedaten:

- Chronische Nierenerkrankung: ICD N18.1, N18.2, N18.3, N18.4, (eGFR < 60 ml/min/1,73 m^2), N18.9 (nicht näher bezeichnet)
- Abrechnung Harnstreifentest: GOP 32033

Zähler: Anzahl der Patienten ≥70 Jahre mit Erstdiagnose CKD (Inzidenzindikator), die eine Urinuntersuchung mittels Streifentest erhalten haben.

Nenner: Anzahl der Patienten ≥70 Jahre mit Erstdiagnose CKD (Inzidenzindikator).

Hintergrund: Mittels Streifentest soll auf eine nicht-sichtbare Hämaturie untersucht werden, welche beispielsweise ein Hinweis auf eine Glomerulonephritis sein kann (DEGAM-Leitlinie).

Anteil der Patienten ≥70 Jahre mit Erstdiagnose CKD (Inzidenzindikator), bei denen eine Urinuntersuchung mittels Streifentest erfolgte.

	stimme nicht zu				stimme teilweise zu				stimme voll zu	Enthaltung
	1	2	3	4	5	6	7	8	9	
Relevanz	○	○	○	○	○	○	○	○	○	○
Wissenschaftlichkeit	○	○	○	○	○	○	○	○	○	○
Praktikabilität (bei Verwendung von Routinedaten)	○	○	○	○	○	○	○	○	○	○

Bitte begründen Sie Ihre Entscheidung und geben Sie Änderungsvorschläge, wenn Sie sich für Enthaltung oder einen Skalenwert von 1-3 entschieden haben.

10. Anteil der Patienten ≥70 Jahre mit Erstdiagnose CKD (GFR < 60 ml/min/1,73 m^2), bei denen in den letzten 12 Monaten (4 Quartale vor Indexquartal) keine CKD diagnostiziert/kodiert wurde, bei denen eine Kontrolle des Blutdrucks erfolgte.

Fokus: Diagnose

Grundlage: „Bei Erstdiagnose einer CKD (GFR < 60 ml/min) soll der Blutdruck kontrolliert werden." (DEGAM-Empfehlung, 6.6)

☐ Routinedaten ☒ Chartreview

Chartreview

Zähler: Anzahl der Patienten ≥70 Jahre mit Erstdiagnose CKD (eGFR < 60 ml/min/1,73 m^2, Inzidenzindikator), bei denen eine Blutdruckmessung in der Praxis durchgeführt wurde.

Nenner: Anzahl der Patienten ≥70 Jahre mit Erstdiagnose CKD (eGFR < 60 ml/min/1,73 m^2, Inzidenzindikator).

Hintergrund: Da ein Bluthochdruck sowohl Ursache als auch Folge einer chronischen Nierenerkrankung sein kann, wird die Messung des Blutdrucks bei Erstdiagnose einer CKD empfohlen (DEGAM-Leitlinie, KDIGO).

Problem: Da die Blutdruckmessung Teil der Ordinationspauschale ist und ihre Durchführung daher nicht aus Routinedaten erfasst werden kann, ist ein Chart Review zur Überprüfung des Qualitätsindikators notwendig. Voraussetzung dafür ist eine Dokumentation des eGFR-Werts und der erfolgten Blutdruckmessung in der Praxis.

Anteil der Patienten ≥70 Jahre mit Erstdiagnose CKD (GFR < 60 ml/min/1,73 m^2), bei denen in den letzten 12 Monaten (4 Quartale vor Indexquartal) keine CKD diagnostiziert/kodierter wurde, bei denen eine Kontrolle des Blutdrucks erfolgte.

	1 stimme nicht zu	2	3	4	5 stimme teilweise zu	6	7	8	9 stimme voll zu	Enthaltung
Relevanz	○	○	○	○	○	○	○	○	○	○
Wissenschaftlichkeit	○	○	○	○	○	○	○	○	○	○
Praktikabilität	○	○	○	○	○	○	○	○	○	○

Bitte begründen Sie Ihre Entscheidung und geben Sie Änderungsvorschläge, wenn Sie sich für Enthaltung oder einen Skalenwert von 1-3 entschieden haben.

Universitätsmedizin GREIFSWALD

11. Anteil der Patienten ≥70 Jahre mit CKD (<60 ml/min/1,73 m²) aber unter 80 Jahre, deren systolischer Blutdruck auf ≤140 mmHg eingestellt ist.

Fokus: Medikamentöse Therapie

Grundlage: „Allen Patienten mit CKD (GFR < 60 ml/min) und Blutdruck > 140/90 mmHg sollten Maßnahmen zur Blutdrucksenkung angeboten werden. Der Zielwert sollte individuell festgelegt werden." (DEGAM-Empfehlung, 7.8)

☐ Routinedaten ☒ Chartreview

Chartreview

- Daten über eGFR-Wert
- Daten über Blutdruck (Angaben über systolischen und diastolischen Blutdruck)

Zähler: Anzahl der Patienten ≥70 Jahre und <80 Jahre mit CKD und mit einem systolischen Blutdruck ≤ 140 mmHg

Nenner: Anzahl der Patienten ≥70 Jahre und <80 Jahre mit CKD

Hintergrund: Hypertonie gilt sowohl als Ursache als auch als Folge einer Chronischen Nierenerkrankung, zudem ist es einer der häufigsten Gründe für eine Dialysepflicht im Verlauf der Erkrankung (DEGAM CKD). Als Bluthochdruck wird das Vorliegen eines systolischen Werts ≥ 140 mmHg und/oder eines diastolischen Werts ≥ 90 mmHg (ESC/ESH 2018) definiert. Abhängig von weiteren Co-Erkrankungen und Risikofaktoren (Diabetes mellitus, Albuminurie) gibt es verschiedene Zielbereiche für die Patienten. Allgemein sollte ein Blutdruck unterhalb von 140/90 mmHg erreicht werden. Es gibt verschiedene blutdrucksenkende Therapien (Lebensstilumstellung, medikamentöse Einstellung) (DEGAM-Leitlinie „Risikoberatung zur kardiovaskulären Prävention"). Die Leitlinienempfehlung (s.o.) wurde für den Qualitätsindikator umformuliert, da nicht jedem Patienten mit einem Blutdruck > 140/90 mmHg medikamentöse Maßnahmen empfohlen werden. Beispielsweise kann bei entsprechendem Alter und kardiovaskulärem Risiko eine Lebensstilumstellung versucht werden, welche als solche nicht als Leistung abgerechnet werden kann. Sowohl der systolische als auch der diastolische Wert sind Prognosefaktoren für kardiovaskuläre Events. Der systolische Wert scheint im höheren Alter eine höhere Bedeutung als Risikofaktor zu haben (ESC/ESH 2018) . Die Leitlinie „KDIGO Clinical practice guideline on the Management of blood pressure management" (2021) legt in ihren Empfehlungen nur einen Zielbereich für den systolischen Wert (<120 mmHg) fest. Dies wird damit begründet, dass aus erniedrigten systolischen Werten häufig auch erniedrigte diastolische Werte resultieren und dass es wenige RCTs (Randomisierte kontrollierte Studien) gibt, die diastolische Werte in Relation mit klinischen Outcomes gesetzt haben, besonders im Zusammenhang mit CKD. Obwohl viele andere Leitlinien sowohl für den systolischen als auch für den diastolischen Wert einen Zielbereich definieren, ist es in der Praxis praktikabler, sich nur am systolischen Wert zu orientieren.

Die Leitlinie NICE und einige Literaturquellen (1-3, siehe unten) empfehlen ein Blutdruckziel von 130/80 mmHg (SPB von 120-129 mmHg) für Patienten mit CKD und Diabetes oder CKD und Proteinurie/Albuminurie (ACR > 300 mg/g). Bei älteren Patienten spielen Komorbiditäten und Arzneimittelinteraktionen mit Antihypertensiva eine wichtige Rolle. Bei manchen Patienten liegt der kleinstmöglich erreichbare Blutdruckwert höher als empfohlen. Aber auch in dieser Patientengruppe werden möglichst niedrige Blutdruckzielwerte empfohlen, da das kardiovaskuläre Risiko gesenkt werden kann (ESC / ESH 2018). Die Datenlage zur Blutdrucktherapie älterer Patienten ist jedoch unzureichend, sodass über die Therapie individuell entschieden werden sollte (DEGAM, „Risikoberatung zur kardiovaskulären Prävention"). Die amerikanischen Leitlinien (ACC/AHA) empfehlen auch bei älteren Pat. <120/80 mmHg. Bei den Europäischen Leitlinien (ESH/ESC) sind die Zielwerte bei älteren Pat. höher (<140/90 mmHg) angegeben. Die Begrenzung auf Patienten unter 80 Jahren erfolgt, weil bei Menschen mit begrenzter Lebenserwartung (ab 80 Jahren) eine individualisierte Therapieentscheidung getroffen werden sollte.

Problem: Es gibt kein festgelegtes Befunddatenfeld für Blutdruck in den in Deutschland zugelassenen Praxisverwaltungssystemen. Deshalb ist ein automatisiertes Auslesen der Blutdruckwerte für eine Analyse der Praxisdaten nicht möglich.

Dieser QI wird auch vorgeschlagen von:

- NICE (QOF): The percentage of patients on the CKD register in whom the last blood pressure reading (measured in the preceding 12 months) is 140/90 mmHg or less (NICE id code NM117)
- ICES: Percentage of patients with chronic kidney disease with a most recent blood pressure < 140/90 mm Hg, or with chronic kidney disease and diabetes with a most recent blood pressure < 130/80 mm Hg.

Literaturquellen:

1. Litvin et al. Quality indicators for primary care: an example for chronic kidney disease. J Ambul Care Manage. 2014; 37:171-8
2. van den Bulck et al. Developing quality indicators for Chronic Kidney Disease in primary care, extractable from the Electronic Medical Record. A Rand-modified Delphi method. BMC Nephrol. 2020; 21:161
3. Bello et al. Quality of Chronic Kidney Disease Management in Canadian Primary Care. JAMA Netw Open 2019

Anteil der Patienten ≥70 Jahre mit CKD (<60 ml/min/1,73 m²) aber unter 80 Jahre, deren systolischer Blutdruck auf <140 mmHg eingestellt ist.

	1 stimme nicht zu	2	3	4	5 stimme teilweise zu	6	7	8	9 stimme voll zu	Enthaltung
Relevanz	○	○	○	○	○	○	○	○	○	○
Wissenschaftlichkeit	○	○	○	○	○	○	○	○	○	○
Praktikabilität	○	○	○	○	○	○	○	○	○	○

Bitte begründen Sie Ihre Entscheidung und geben Sie Änderungsvorschläge, wenn Sie sich für Enthaltung oder einen Skalenwert von 1-3 entschieden haben.

Universitätsmedizin GREIFSWALD

12. Anteil der Patienten ≥70 Jahre mit CKD und eGFR < 30 ml/min/1,73 m², bei denen NSAR verordnet wurden.

Fokus: Medikamentöse Therapie

Grundlage: „Nicht-Steroide Antirheumatika (NSAR) sollten bei Patienten mit einer CKD bis zu einer GFR ≥ 30 ml/min möglichst vermieden werden oder nur so kurz und niedrig dosiert wie möglich eingesetzt werden. Ab einer GFR < 30 sind NSAR kontraindiziert." (DEGAM-Empfehlung, 7.17)

☒ Routinedaten ☒ Chartreview

Routinedaten:

- CKD-Stadium 4 (eGFR < 30ml/min/1,73 m^2): ICD-10 N18.4
- N18.9 (nicht näher klassifiziert)
- Keine der folgenden ATC-Codes NSAR: M01A (ASS als Thrombozytenhemmer → B01AC06, Analgetikum extra → N02BA01)

Chart Review:

- Angabe des eGFR Werts
- Angabe von Verordnung von ASS, Diclofenac, Ibuprofen, Coxibe

Zähler: Anzahl der Patienten ≥70 Jahre mit CKD und eGFR < 30 ml/min/1,73 m^2, die in den letzten 4 Quartalen NSAR verordnet bekommen haben.

Nenner: Anzahl der Patienten ≥70 Jahre mit CKD und eGFR < 30 ml/min/1,73 m^2 in den letzten 4 Quartalen.

Hintergrund: NSAR sollen bei Patienten mit Chronischer Nierenerkrankung vermieden werden. Sie können unter anderem eine akute Nierenschädigung auslösen. Ab einer eGFR < 30 ml/min/1,73 m^2 sind sie laut Fachinformation kontraindiziert (DEGAM-Leitlinie). Eine Alternative des Qualitätsindikators könnte „Anteil der Patienten mit CKD, bei denen NSAR verordnet wurden" sein, da auch in den früheren Stadien die NSAR-Verordnung vermieden werden soll.

Problem: Für die Anwendung des Qualitätsindikators mithilfe von Routinedaten ist die genaue Verschlüsselung des CKD-Stadiums (ICD N18.4, eGFR < 30 ml/min/1,73 m^2) notwendig. Ein Teil der Patienten wird mit ICD N18.9 verschlüsselt, was zu einer Überschätzung der Versorgungsqualität führen kann. Es handelt sich hier lediglich um die verordneten NSAR. Frei verkäufliche NSAR werden mit Routinedaten nicht erfasst und können daher nicht berücksichtigt werden. **N18.9 überschätzt möglicherweise Patienten mit Kontraindikation. Es lässt sich nicht ausschließen, ob es sich dabei um partizipative Entscheidung/Patientenwunsch nach Aufklärung handelt.**

Der Indikator sollte optimalerweise bei 0% liegen.

Dieser QI wird auch vorgeschlagen von:

- ICES: Percentage of patients with stage 3–5 chronic kidney disease and a prescription for a nonsteroidal anti-inflammatory drug for more than 2 weeks..

Anteil der Patienten ≥70 Jahre mit CKD und eGFR < 30 ml/min/1,73 m², bei denen NSAR verordnet wurden.

	1 (stimme nicht zu)	2	3	4	5 (stimme teilweise zu)	6	7	8	9 (stimme voll zu)	Enthaltung
Relevanz	○	○	○	○	○	○	○	○	○	○
Wissenschaftlichkeit	○	○	○	○	○	○	○	○	○	○
Praktikabilität (bei Verwendung von Routinedaten)	○	○	○	○	○	○	○	○	○	○

Bitte begründen Sie Ihre Entscheidung und geben Sie Änderungsvorschläge, wenn Sie sich für Enthaltung oder einen Skalenwert von 1-3 entschieden haben.

13. Anteil der Patienten ≥70 Jahre mit CKD, deren eGFR entsprechend der Monitoringintervalle (abhängig vom eGFR- und Albuminuriewert) kontrolliert wurde. (siehe Abbildung)

Fokus: Monitoring

Grundlage: „Patienten mit CKD mit und ohne Diabetes, sollten in regelmäßigen Abständen entsprechend CKD-Stadien (s. Abb. 2, s.u.) eine Bestimmung der eGFR aus Serumkreatinin angeboten bekommen.“ (DEGAM-Empfehlung, 8.2)

☐ Routinedaten ☒ **Chartreview**

Chartreview

Zähler: Anzahl der Patienten ≥70 Jahre mit CKD, deren eGFR-Wert entsprechend der Monitoringintervalle kontrolliert wurde

Nenner: Anzahl der Patienten ≥70 Jahre mit CKD

Hintergrund: Die eGFR bei Patienten mit CKD sollte regelmäßig kontrolliert werden. In Abhängigkeit vom Stadium der CKD (abhängig von eGFR und Albuminurie) sowie Komorbiditäten, Allgemeinzustand und Wunsch des Patienten sollten die Intervalle mit dem Patienten gemeinsam abgestimmt werden. (DEGAM-Leitlinie, NICE)

Problem: Der ACR-Wert ist in Routinedaten nicht verfügbar. Dies ist jedoch wichtig für die vorgeschlagene Einordnung in die Monitoringintervalle. Daher wird hier die Umsetzung mittels Chart Review festgelegt.

Dieser QI wird auch vorgeschlagen von:

- ICES: Percentage of patients with chronic kidney disease with an eGFR in the past 18 months.

Empfehlungen für die Monitoringfrequenz (Anzahl Kontrolluntersuchungen pro Jahr) anhand GFR- und Albuminuriestadien

GFR Stadien (ml/min/1,73m²) Beschreibung und Intervall			Stadien der persistierenden Albuminurie Beschreibung und Intervall: A1 normal bis leichtgradig eingeschränkt <30 mg/g <3mg/mmol	A2 mäßiggradig eingeschränkt 30-300 mg/g 3-30 mg/mmol	A3 hochgradig eingeschränkt >300 mg/g <30mg/mmol
G1	normal oder hoch	≥90	≤1 (wenn CKD vorliegt)	1	≥1
G2	leichtgradig eingeschränkt*	60-89	≤1 (wenn CKD vorliegt)	1	≥1
G3a	leicht- bis mäßiggradig eingeschränkt	45-59	1	1	2
G3b	mäßig- bis hochgradig eingeschränkt	30-44	≤2	2	≥2
G4	hochgradig eingeschränkt	15-29	2	2	3
G5	terminale Niereninsuffizienz	<15	4	≥4	≥4

zunehmendes Risiko (nach unten)

*Im Vergleich zum Jungerwachsenen

zunehmendes Risiko (nach rechts)

Anteil der Patienten ≥70 Jahre mit CKD, deren eGFR entsprechend der Monitoringintervalle (abhängig vom eGFR- und Albuminuriewert) kontrolliert wurde. (siehe Abbildung)

	1 (stimme nicht zu)	2	3	4	5 (stimme teilweise zu)	6	7	8	9 (stimme voll zu)	Enthaltung
Relevanz	○	○	○	○	○	○	○	○	○	○
Wissenschaftlichkeit	○	○	○	○	○	○	○	○	○	○
Praktikabilität	○	○	○	○	○	○	○	○	○	○

Bitte begründen Sie Ihre Entscheidung und geben Sie Änderungsvorschläge, wenn Sie sich für Enthaltung oder einen Skalenwert von 1-3 entschieden haben.

14. Anteil der Patienten ≥70 Jahre mit CKD, deren eGFR-Wert bei CKD Stadium 1-3 oder unspezifisch kodiert mind. 1x im Jahr und CKD Stadium 4 mind. 2x im Jahr kontrolliert wurde. (Unabhängig vom Albuminuriewert, der in Routinedaten nicht zur Verfügung steht)

Fokus: Monitoring

Grundlage: „Patienten mit CKD mit und ohne Diabetes, sollten in regelmäßigen Abständen entsprechend CKD-Stadien (s. Abb. 2, s.u.) eine Bestimmung der eGFR aus Serumkreatinin angeboten bekommen." (DEGAM-Empfehlung, 8.2)

☒ **Routinedaten** ☐ Chartreview

Routinedaten:

ICD-10 Code:

- CKD Stadien: N18.1, N18.2, N18.3, N18.9: eGFR Kontrolle mind. 1x/Jahr
- CKD Stadium: N18.4: eGFR Kontrolle mind. 2x/Jahr

Abrechnung eGFR und Serumkreatininbestimmung: GOP 32066, 32067

Zähler: Anzahl der Patienten ≥70 Jahre mit CKD, deren eGFR-Wert entsprechend ihres CKD-Stadiums kontrolliert wurde.

Nenner: Anzahl der Patienten ≥70 Jahre mit CKD

Hintergrund: Die eGFR bei Patienten mit CKD sollte regelmäßig kontrolliert werden. Die Kontrollintervalle sollen abhängig vom CKD Stadium, aber unabhängig vom Albuminuriewert erfolgen. Dies ist eine Vereinfachung des vorherigen Qualitätsindikators, da der Albuminuriewert in Routinedaten nicht verfügbar ist. So kann dieser Qualitätsindikator mittels Routinedaten umgesetzt werden.

Problem: Es handelt sich um eine pragmatische Vergröberung.

Dieser QI wird auch vorgeschlagen von:

- ICES: Percentage of patients with chronic kidney disease with an eGFR in the past 18 months.

Anteil der Patienten ≥70 Jahre mit CKD, deren eGFR bei CKD Stadium 1-3 oder unspezifisch kodiert mind. 1x im Jahr und CKD Stadium 4 mind. 2x im Jahr kontrolliert wurde. (Unabhängig vom Albuminuriewert)

	1 (stimme nicht zu)	2	3	4	5 (stimme teilweise zu)	6	7	8	9 (stimme voll zu)	Enthaltung
Relevanz	○	○	○	○	○	○	○	○	○	○
Wissenschaftlichkeit	○	○	○	○	○	○	○	○	○	○
Praktikabilität	○	○	○	○	○	○	○	○	○	○

Bitte begründen Sie Ihre Entscheidung und geben Sie Änderungsvorschläge, wenn Sie sich für Enthaltung oder einen Skalenwert von 1-3 entschieden haben.

15. Anteil der Patienten ≥70 Jahre mit CKD, bei denen mindestens einmal jährlich der Blutdruck dokumentiert wurde.

Fokus: Monitoring

Grundlage: „Patienten mit CKD sollte in regelmäßigen Abständen mindestens entsprechend der individuell vereinbarten Monitoringintervalle eine Messung des Blutdrucks angeboten werden.“ (DEGAM-Empfehlung, 8.3)

☐ Routinedaten ☒ Chartreview

Chartreview

- Patienten mit CKD
- Blutdruckmessung in der Praxisverwaltungssoftware oder Papierakte

Zähler: Anzahl der Patienten ≥70 Jahre mit CKD, deren Blutdruckwert einmal jährlich gemessen wurde.

Nenner: Anzahl der Patienten ≥70 Jahre mit CKD

Hintergrund: Bluthochdruck kann sowohl Ursache als auch Folge der Chronischen Nierenerkrankung sein und die Progression der Erkrankung beeinflussen. (DEGAM-Leitlinie)

Problem: Da die Blutdruckmessung Teil der Ordinationspauschale ist, kann ihre Durchführung nicht aus Routinedaten erfasst werden. Für den Qualitätsindikator eignet sich das Chart Review. Voraussetzung dafür ist die Dokumentation der erfolgten Blutdruckmessung in der Praxis. In dem Vorschlag wird nur die Messung, aber nicht die Höhe des Blutdrucks berücksichtigt.

Dieser QI wird auch vorgeschlagen von:

- ICES: Percentage of patients with chronic kidney disease with a blood pressure recorded in the past 18 months.
- ICES: Percentage of patients with diabetes and albuminuria (moderately or severely increased ACR ≥ 3 mg/mmol) with a blood pressure recorded in the past 9 months.

Anteil der Patienten ≥70 Jahre mit CKD, bei denen mindestens einmal jährlich der Blutdruck dokumentiert wurde.

	stimme nicht zu				stimme teilweise zu				stimme voll zu	Enthaltung
	1	2	3	4	5	6	7	8	9	
Relevanz	○	○	○	○	○	○	○	○	○	○
Wissenschaftlichkeit	○	○	○	○	○	○	○	○	○	○
Praktikabilität	○	○	○	○	○	○	○	○	○	○

Bitte begründen Sie Ihre Entscheidung und geben Sie Änderungsvorschläge, wenn Sie sich für Enthaltung oder einen Skalenwert von 1-3 entschieden haben.

Universitätsmedizin GREIFSWALD

16. Anteil der Patienten ≥70 Jahre mit CKD und nachgewiesener Proteinurie, deren Albumin-Kreatinin-Ratio mindestens einmal jährlich gemessen wurde.

Fokus: Monitoring

Grundlage: „Patienten mit CKD und einer nachgewiesenen Proteinurie sollte in regelmäßigen Abständen mindestens entsprechend der individuell vereinbarten Monitoringintervalle (siehe Abbildung 2) eine Bestimmung der Albumin-Kreatinin-Ratio angeboten werden." (DEGAM, 8.5)

☒ Routinedaten ☒ Chartreview

Routinedaten:

- Chronische Nierenerkrankung ICD: N18.1, N18.2, N18.3, 18.4, N18.9
- Proteinurie ICD: N06
- Bestimmung der ACR mittels Spontanurinprobe (spot urine) oder 24-h-Sammelurin: Kreatinin GOP 32066, 32067; Albumin GOP 32435 (beides muss zur gleichen Zeit abgerechnet worden sein, das es keine GOP für ACR gibt.)

Zähler: Anzahl der Patienten ≥70 Jahre mit CKD und nachgewiesener Proteinurie, bei denen mindestens einmal jährlich die Kreatinin-/Albumin-Ratio gemessen wurde

Nenner: Anzahl der Patienten ≥70 Jahre mit CKD und nachgewiesener Proteinurie

Hintergrund: Eine Proteinurie kann ein Hinweis auf eine Nephropathie und ein Marker für ein erhöhtes kardiovaskuläres Risiko sein. (DEGAM-Leitlinie). Die NICE-Leitlinie empfiehlt die Bestimmung der ACR zusammen mit der eGFR. Die KDIGO empfiehlt die Messung mindestens einmal jährlich. Der Nutzen einer regelmäßigen Kontrolle der Proteinurie bei initial ausgeschlossener Proteinurie ist nicht belegt. (DEGAM-Leitlinie)

Problem: Es existiert keine eindeutige GOP, mit der die ACR abgerechnet werden kann. Das Labor ermittelt ACR nur, wenn Albumin und Kreatinin abgerechnet worden sind. Die Proteinurie wird sehr wahrscheinlich schlecht verschlüsselt.

Dieser QI wird auch vorgeschlagen von:

- NICE QOF: The percentage of patients on the CKD register whose notes have a record of a urine albumin:creatinine ratio (or protein:creatinine ratio) test in the preceding 12 months (NICE id code NM109)
- ICES: Percentage of patients with risk factors for chronic kidney disease (diabetes, hypertension) with an ACR in the past 18 months.
- ICES: Percentage of patients with chronic kidney disease with an ACR in the past 18 months.

Anteil der Patienten ≥70 Jahre mit CKD und nachgewiesener Proteinurie, deren Albumin-Kreatinin-Ratio mindestens einmal jährlich gemessen wurde.

	1 (stimme nicht zu)	2	3	4	5 (stimme teilweise zu)	6	7	8	9 (stimme voll zu)	Enthaltung
Relevanz	○	○	○	○	○	○	○	○	○	○
Wissenschaftlichkeit	○	○	○	○	○	○	○	○	○	○
Praktikabilität (bei Verwendung von Routinedaten)	○	○	○	○	○	○	○	○	○	○

Bitte begründen Sie Ihre Entscheidung und geben Sie Änderungsvorschläge, wenn Sie sich für Enthaltung oder einen Skalenwert von 1-3 entschieden haben.

17. Anteil der Patienten ≥70 Jahre mit CKD und ohne bekannte Anämie, bei denen einmal jährlich (Stadium 3) oder halbjährlich (Stadium 4) der Hämoglobinwert bestimmt wurde.

Fokus: Monitoring

Grundlage: „Der Serumhämoglobinwert (Hb) sollte bei Patienten ohne bekannte Anämie mit CKD-Stadium 3b einmal jährlich und CKD-Stadium 4-5 halbjährlich bestimmt werden, um eine behandlungsbedürftige Anämie auszuschließen." (DEGAM-Empfehlung, 8.7)

☒ Routinedaten ☒ Chartreview

Routinedaten:

- ICD N18.3 (eGFR < 60 ml/min/1,73 m^2), N18.4 (eGFR < 30 ml/min/1,73 m^2)
- GOP 32120 (mechanisiertes Blutbild), 32038 (Hämoglobin einzeln)

Zähler: Anzahl der Patienten ≥70 Jahre mit CKD Stadium 3 oder 4, bei denen 1x jährlich (eGFR < 60 ml/min/1,73 m^2) bzw. 2x jährlich (eGFR < 30 ml/min/1,73 m^2) der Hämoglobinwert im Blut bestimmt wurde.

Nenner: Anzahl der Patienten ≥70 Jahre mit CKD Stadium 3 oder 4

Hintergrund: Die Anämie ist eine Komplikation der CKD, vor allem in den höheren Stadien. Wichtige Ursachen sind der Mangel an Erythropoetin sowie eine Störung des Eisenhaushalts. Es wird eine jährliche Kontrolle des Hb empfohlen, aufgrund des hohen Anteils von Patienten ohne Anämie im Stadium 3a aber erst ab Stadium 3b (eGFR: 44 -30 ml/min/1,73 m^2), und halbjährlich bei einer eGFR < 30 ml/min/1,73 m^2. (DEGAM-Leitlinie). Da sich das Stadium 3b nicht in den Routinedaten ableiten lässt, wurde der Indikator auf Stadium 3 vergröbert.

Problem: Da sich das Stadium 3b nicht in ICD-Codes ableiten lässt (ICD N18.3: eGFR 59-30 ml/min/1,73 m^2), kann die Verwertung von Routinedaten ungenau sein. Eine genaue Überprüfung ab Stadium 3b lässt sich nur mittels Chart Review durchführen.

Anteil der Patienten ≥70 Jahre mit CKD und ohne bekannte Anämie, bei denen einmal jährlich (Stadium 3) oder halbjährlich (Stadium 4) der Hämoglobinwert bestimmt wurde.

	1 (stimme nicht zu)	2	3	4	5 (stimme teilweise zu)	6	7	8	9 (stimme voll zu)	Enthaltung
Relevanz	○	○	○	○	○	○	○	○	○	○
Wissenschaftlichkeit	○	○	○	○	○	○	○	○	○	○
Praktikabilität (bei Verwendung von Routinedaten)	○	○	○	○	○	○	○	○	○	○

Bitte begründen Sie Ihre Entscheidung und geben Sie Änderungsvorschläge, wenn Sie sich für Enthaltung oder einen Skalenwert von 1-3 entschieden haben.

18. Anteil der Patienten ≥70 Jahre mit CKD und erstmals festgestellter eGFR <30 ml/min/1,73m², die an einen Nephrologen überwiesen wurden.

Fokus: Diagnose

Grundlage: „Bei jeder erstmals festgestellten CKD mit eGFR < 30 ml/min sollte eine Überweisung in die Nephrologie zu einer Konsiliaruntersuchung angeboten werden." (DEGAM, 6.9)

☒ Routinedaten ☒ Chartreview

Routinedaten:

- ICD-Code N18.4, bei denen N18.4 in den letzten 12 Monaten (4 Quartale) nicht verschlüsselt wurde
- Überweisung an einen Nephrologen (→ GOP 13592 Grundpauschale ab dem 60. Lebensjahr)

Chart Review:

- Angabe eGFR
- Angabe Überweisung an einen Nephrologen

Zähler: Anzahl der Patienten ≥70 Jahre mit CKD und erstmals festgestellter eGFR < 30 ml/min/1,73 m^2 (keine eGFR < 30 ml/min in den letzten 4 Quartalen), die an einen Nephrologen überwiesen wurden.

Nenner: Anzahl der Patienten ≥70 Jahre mit CKD und erstmals festgestellter eGFR < 30 ml/min/1,73 m^2

Hintergrund: Die Überweisung zum Nephrologen von Patienten mit einer eGFR < 30 ml/min dient dem Erkennen einer potentiell behandelbaren und reversiblen Ursache der Nierenfunktionsstörung sowie der Abschätzung der Notwendigkeit weiterer Maßnahmen (Dialyse, Transplantation). Bei der Indikationsstellung sollen Komorbiditäten, Lebenserwartung und individuelle Gesundheitsziele der Patienten berücksichtigt werden (DEGAM-Leitlinie). Während bei jüngeren Patienten mit eingeschränkter Nierenfunktion die Indikation zur Überweisung an die Nephrologie großzügiger gestellt werden sollte, sollte bei der Indikation zur Überweisung älterer Patienten deren Komorbiditäten und individuelle Gesundheitsziele bzw. Lebenserwartung berücksichtigt werden. Somit wird für den Indikator nicht erwartet, dass jeder Patient mit einer eGFR unter 30 ml/min überwiesen wird, sondern berücksichtigt, dass es ältere multimorbide Patienten gibt, für die eine Überweisung keinen wesentlichen Vorteil bringt (DEGAM). Andere Leitlinien (KDIGO, NICE) und Literaturquellen (siehe unten) geben weitere Empfehlungen zur Überweisungsindikation an, wie beispielsweise bei einer Albuminurie (ACR ≥ 300 mg/g).

Problem: In Routinedaten ist die Überweisung zum Nephrologen nur dann ersichtlich, wenn der Patient sich dort tatsächlich vorgestellt hat. Eine erfolgte Überweisung vom Hausarzt, ohne dass der Patient zum Nephrologen gegangen ist, ist nicht überprüfbar. Das unter- oder überschätzt die Leitlinienadhärenz.

Dieser QI wird auch vorgeschlagen von:

- ICES [7]: Percentage of patients age < 80 years with a referral to a nephrologist for eGFR < 30 mL/min per 1.73 m^2.

Literatur:

Litvin CB et al. Quality indicators for primary care: an example for chronic kidney disease. J Ambul Care Manage. 2014;37:171-8.

van den Bulck et al. Developing quality indicators for chronic kidney disease in primary care, extractable from the electronic medical record. A rand-modified Delphi method. BMC Nephrol. 2020;21:161

Anteil der Patienten ≥70 Jahre mit CKD und erstmals festgestellter eGFR <30 ml/min/1,73 m², die an einen Nephrologen überwiesen wurden.

	1 (stimme nicht zu)	2	3	4	5 (stimme teilweise zu)	6	7	8	9 (stimme voll zu)	Enthaltung
Relevanz	○	○	○	○	○	○	○	○	○	○
Wissenschaftlichkeit	○	○	○	○	○	○	○	○	○	○
Praktikabilität (bei Verwendung von Routinedaten)	○	○	○	○	○	○	○	○	○	○

Bitte begründen Sie Ihre Entscheidung und geben Sie Änderungsvorschläge, wenn Sie sich für Enthaltung oder einen Skalenwert von 1-3 entschieden haben.

Universitätsmedizin GREIFSWALD

19. Anteil der Patienten ≥70 Jahre mit CKD und eGFR < 30 ml/min/1,73 m² und Diabetes Typ 2, bei denen Metformin verordnet wurde.

Fokus: Medikamentöse Therapie

Grundlage: „Metformin sollte bei CKD und Diabetes Typ 2 bis zu einer eGFR ≥ 30 ml/min bevorzugt angeboten werden, wenn der HbA1C nicht im individuellen Zielbereich liegt." (DEGAM, 7.9)

☒ Routinedaten ☒ Chartreview

Routinedaten:

- Diabetes ICD-Code: ICD: E11 (DM Typ 2)
- CKD ICD-Codes: ICD: N18.4 (eGFR ≤ 30 ml/min), N18.9
- Metformin-ATC-Codes (keine der folgenden Codes): Monotherapie: A10BA02; Kombinationstherapie: A10BD02, A10BD03, A10BD05, A10BD07, A10BD08, A10BD10, A10BD11, A10BD13, A10BD14, A10BD15, A10BD16, A10BD17, A10BD18, A10BD31

Chart Review:

- Angaben über den eGFR-Wert
- Angabe über Diabetes-Erkrankung
- Angabe blutzuckersenkende Medikamente sowie Einnahme von Metformin

Zähler: Anzahl der Patienten ≥70 Jahre mit CKD und eGFR < 30 ml/min/1,73 m^2 und Diabetes mellitus Typ 2, die Metformin verordnet bekommen haben.

Nenner: Anzahl der Patienten ≥70 Jahre mit CKD und eGFR < 30 ml/min/1,73 m^2 und Diabetes mellitus Typ 2

Hintergrund: Metformin wird in der medikamentösen Therapie des Diabetes mellitus 2 eingesetzt. Die KDIGO empfiehlt Metformin bei Patienten mit CKD und einer eGFR < 30 ml/min/1,73 m^2 aufgrund der Arzneimittelsicherheit nicht einzusetzen. Auch die DEGAM empfiehlt den Einsatz von Metformin nur bei einer eGFR > 30 ml/min/1,73 m^2. Bei einer eingeschränkten Nierenfunktion und Diabetes ist unter Metformintherapie die Gefahr einer metabolischen Azidose (Lactatazidose) erhöht und deshalb Metformin kontraindiziert. (DEGAM-Leitlinie) Die Leitlinienempfehlung wurde umformuliert, da nicht alle Patienten mit Diabetes eine medikamentöse Therapie erhalten, sondern auch durch eine Lebensstilumstellung der HbA1C-Wert adäquat eingestellt werden kann. In diesem Fall würde Metformin nicht verordnet werden.

Der Indikator sollte optimalerweise bei 0% liegen.

Problem: Für die Anwendung des Qualitätsindikators mithilfe von Routinedaten ist die genaue Verschlüsselung des CKD-Stadiums (ICD N18.4, eGFR < 30 ml/min/1,73 m^2, CKD-Stadium 4) notwendig. Ein Teil der Patienten wird mit ICD N 18.9 verschlüsselt, was zu einer Überschätzung der Versorgungsqualität führen kann.

Anteil der Patienten ≥70 Jahre mit CKD und eGFR < 30 ml/min/1,73 m² und Diabetes Typ 2, bei denen Metformin verordnet wurde.

	1 stimme nicht zu	2	3	4	5 stimme teilweise zu	6	7	8	9 stimme voll zu	Enthaltung
Relevanz	○	○	○	○	○	○	○	○	○	○
Wissenschaftlichkeit	○	○	○	○	○	○	○	○	○	○
Praktikabilität (bei Verwendung von Routinedaten)	○	○	○	○	○	○	○	○	○	○

Bitte begründen Sie Ihre Entscheidung und geben Sie Änderungsvorschläge, wenn Sie sich für Enthaltung oder einen Skalenwert von 1-3 entschieden haben.

Universitätsmedizin GREIFSWALD

20. Anteil der Patienten ≥70 Jahre mit CKD und Anämie (Hb < 6,8 mmol/l / <11 mg/dl), bei denen zur Überprüfung des Eisenstatus der Serumferritinwert mit Serumtransferrinsättigung bestimmt wurde.

Fokus: Monitoring

Grundlage: „Bei Patienten mit CKD und einem Hämoglobin < 11 mg/dl (< 6,8 mmol/l) sollten zur Überprüfung des Eisenstatus der Serumferritinwert in Kombination mit der Serumtransferrinsättigung (TSAT) erhoben werden." (DEGAM-Empfehlung, 8.8)

☐ Routinedaten ☒ Chartreview

Zähler: Anzahl der Patienten ≥70 Jahre mit CKD und Anämie (Hb < 6,8 mmol/l / < 11 mg/dl), bei denen der Serumferritinwert und die Serumtransferrinsättigung bestimmt wurde.

Nenner: Anzahl der Patienten ≥70 Jahre mit CKD und Anämie (Hb < 6,8 mmol/l / < 11 mg/dl)

Hintergrund: Zur Überprüfung des Eisenhaushaltes ist es sinnvoll, bei erniedrigtem Hb den Serumferritinwert und die Serumtransferrinsättigung zu bestimmen. Bei einer Eisenverwertungsstörung ist der Ferritinwert normal bis erhöht und die Serumtransferrinsättigung erniedrigt. (DEGAM-Leitlinie)

Problem: Der Hämoglobinmesswert ist nicht in Routinedaten verfügbar, daher wird hier ausschließlich auf die Nutzung des Chat Reviews verwiesen.

Anteil der Patienten ≥70 Jahre mit CKD und Anämie (Hb < 6,8 mmol/l / <11 mg/dl), bei denen zur Überprüfung des Eisenstatus der Serumferritinwert mit Serumtransferrinsättigung bestimmt wurde.

	1 stimme nicht zu	2	3	4	5 stimme teilweise zu	6	7	8	9 stimme voll zu	Enthaltung
Relevanz	○	○	○	○	○	○	○	○	○	○
Wissenschaftlichkeit	○	○	○	○	○	○	○	○	○	○
Praktikabilität	○	○	○	○	○	○	○	○	○	○

Bitte begründen Sie Ihre Entscheidung und geben Sie Änderungsvorschläge, wenn Sie sich für Enthaltung oder einen Skalenwert von 1-3 entschieden haben.

21. Anteil der Patienten ≥70 Jahre mit CKD und Hypertonie, die im Rahmen der Therapie ACE-Hemmer oder ARB erhalten.

Fokus: Medikamentöse Therapie

Grundlage:

- "Patients on RAS inhibitors / CKD patients with hypertension meeting criteria A and (B or C) as follows: A, patients labeled as having hypertension in administrative claims data; B, CKD patients with diabetes mellitus (ICD10); C; CKD patients without diabetes mellitus (ICD10) but with proteinuria" [Fukuma et al. Development of quality indicators for care of chronic kidney disease in the primary care setting using electronic health data: a RAND-modified Delphi method. Clin Exp Nephrol. 2017,21:247-56]
- „Prescription of ACE inhibitor or angiotensin receptor blocker recorded in past year for patients with CKD and hypertension with proteinuria“ [Litvin CB et al. Quality incicators for primary care: an example for chronic kidney disease. J Ambul Care Manage.2014;37:171-8]
- „We recommend that an ARB or ACE-I be used in both diabetic and non-diabetic adults with CKD and urine albumin excretion > 300 mg/24 hours (or equivalent).“ [KDIGO]
- „Offer a low-cost renin–angiotensin system antagonist to people with CKD and: diabetes and an ACR of 3 mg/mmol or more (ACR category A2 or A3); hypertension and an ACR of 30 mg/mmol or more (ACR category A3), an ACR of 70 mg/mmol or more (irrespective of hypertension or cardiovascular disease)“ [NICE]

☒ Routinedaten ☒ Chartreview

Routinedaten:

- ICD Hypertonie: I10-I15
- ICD CKD: N18.1, N.18.2, N18.3, N18.4, N18.9
- ATC-Codes ACE: C09A, C09B, (und in Kombination)
- ATC Angiotensin-II-Rezeptor-Blocker (ARB): C09C, C09 D (in Kombination)

Zähler: Anzahl der Patienten ≥70 Jahre mit CKD und Hypertonie (ICD I10-I15), die ACE-Hemmer oder ARB verordnet bekommen haben.

Nenner: Anzahl der Patienten ≥70 Jahre mit CKD und Hypertonie (ICD I10-I15)

Hintergrund: Erwartete Therapievorteile von ACE-Hemmern und ARB sind die Reduktion der kardiovaskulären Mortalität sowie die Reduktion der Proteinurie und Progression von CKD (NICE). Es gibt Leitlinienempfehlungen (KDIGO, NICE), die die Anwendung von ACE-Hemmern oder ARB auch bei Patienten mit CKD und Diabetes und Albuminurie (30-300 mg/24h) oder CKD und Albuminurie über 300 mg/24 h vorschlagen.

Problem: Für die Anwendung des Qualitätsindikators mithilfe von Routinedaten ist die genaue Verschlüsselung der Proteinurie notwendig, die oft nicht erfolgt. Daher kann dieser Aspekt nicht berücksichtigt werden.

Dieser QI wird auch vorgeschlagen von:

- NICE (QOF): The percentage of patients on the CKD register who have hypertension and proteinuria and who are currently being treated with renin-angiotensin system antagonists (NICE id code: NM84)
- ICES: Percentage of patients with diabetes and albuminuria (moderately or severely increased ACR ≥ 3 mg/mmol) who were prescribed an angiotensin-converting enzyme (ACE) inhibitor or angiotensin II receptor blocker (ARB) unless a contraindication or adverse effect are recorded.

Anteil der Patienten ≥70 Jahre mit CKD und Hypertonie, die im Rahmen der Therapie ACE-Hemmer oder ARB erhalten.

	stimme nicht zu				stimme teilweise zu				stimme voll zu	Enthaltung
	1	2	3	4	5	6	7	8	9	
Relevanz	○	○	○	○	○	○	○	○	○	○
Wissenschaftlichkeit	○	○	○	○	○	○	○	○	○	○
Praktikabilität (bei Verwendung von Routinedaten)	○	○	○	○	○	○	○	○	○	○

Bitte begründen Sie Ihre Entscheidung und geben Sie Änderungsvorschläge, wenn Sie sich für Enthaltung oder einen Skalenwert von 1-3 entschieden haben.

22. Anteil der Patienten ≥70 Jahre mit CKD, die in den letzten 12 Monaten eine Grippeschutzimpfung erhalten haben, falls nicht kontraindiziert.

Fokus: Monitoring

Grundlage: "Percentage of patients with chronic kidney disease who received an influenza vaccine in the past year unless contraindicated." (ICES)

☒ Routinedaten ☒ Chartreview

Routinedaten:

- Grippeschutzimpfung/Influenza Impfung: GOP 89111 (Standardimpfung, Personen ab 60 Jahre), GOP 89112 (Influenza Indikationsimpfung)
- Prävalenzindikator CKD

Zähler: Anzahl der Patienten ≥70 Jahre mit CKD und Influenza-Impfung in den letzten 12 Monaten.

Nenner: Anzahl der Patienten ≥70 Jahre mit CKD

Hintergrund: Die STIKO empfiehlt die Influenzaimpfung für Personen mit erhöhter Gefährdung infolge eines Grundleidens, dazu zählen auch Nierenkrankheiten. Personen mit Grundkrankheiten haben ein erhöhtes Risiko, schwere oder tödliche Krankheitsverläufe einer Influenzainfektion zu entwickeln.

Problem: Die Entscheidung zur Grippeschutzimpfung liegt beim Patienten. Der Arzt/die Ärztin kann lediglich aufklären. Kontraindikationen sind schwer operationalisierbar.

Anteil der Patienten ≥70 Jahre mit CKD, die in den letzten 12 Monaten eine Grippeschutzimpfung erhalten haben, falls nicht kontraindiziert.

	1 stimme nicht zu	2	3	4	5 stimme teilweise zu	6	7	8	9 stimme voll zu	Enthaltung
Relevanz	○	○	○	○	○	○	○	○	○	○
Wissenschaftlichkeit	○	○	○	○	○	○	○	○	○	○
Praktikabilität (bei Verwendung von Routinedaten)	○	○	○	○	○	○	○	○	○	○

Bitte begründen Sie Ihre Entscheidung und geben Sie Änderungsvorschläge, wenn Sie sich für Enthaltung oder einen Skalenwert von 1-3 entschieden haben.

Universitätsmedizin GREIFSWALD

23. Anteil der Patienten ≥70 Jahre mit CKD, die über mindestens 2 Quartale mehrfach (≥2) gleichzeitig ACE-Hemmer und ARB (Angiotensin-II-Rezeptorblocker) innerhalb von 3 Quartalen nach dem Quartal mit der ersten Doppelverordnung verordnet bekommen.

Fokus: Medikamentöse Therapie

Grundlage: "Percentage of patients with chronic kidney disease simultaneously receiving both an ACE inhibitor and an ARB." (ICES)

☒ Routinedaten ☒ Chartreview

Routinedaten:

- Prävalenzindikator CKD
- ACE-Hemmer ATC Code: C09A, C09B, C09BB, C09BX
- ARB ATC Code: C09C, C09D

Zähler: Anzahl der Patienten ≥70 Jahre mit CKD und gleichzeitiger Verschreibung von ACE-Hemmern und ARB in mind. 2 Quartalen innerhalb von 4 Quartalen nach der ersten Doppelverordnung

Nenner: Anzahl der Patienten ≥70 Jahre mit CKD im gleichen Beobachtungszeitraum wie Zähler

Hintergrund: Die Kombination beider Medikamente führt zu vermehrten Nebenwirkungen und wahrscheinlich zu einer erhöhten Mortalität.

Problem: Bei Unverträglichkeit eines der Medikamente (ACE-Hemmer oder ARB) kann der Arzt/die Ärztin auf die jeweilige andere Medikamentengruppe umstellen. Das würde bedeuten, dass die Verschreibung von beiden Medikamenten in einem Quartal erfolgen kann. Um sicher zu gehen, dass es sich nicht um eine Medikamentenumstellung handelt, werden hier mind. 2 Quartale berücksichtigt, in der Annahme, dass erst eine weitere zeiliche überlappende Verordnung eine gleichzeitige Einnahme von ACE-Hemmern und ARB auseichend belegt.

Anteil der Patienten ≥70 Jahre mit CKD, die über mindestens 2 Quartale mehrfach (≥2) gleichzeitig ACE-Hemmer und ARB (Angiotensin-II-Rezeptorblocker) innerhalb von 3 Quartalen nach dem Quartal mit der ersten Doppelverordnung verordnet bekommen.

	1 (stimme nicht zu)	2	3	4	5 (stimme teilweise zu)	6	7	8	9 (stimme voll zu)	Enthaltung
Relevanz	○	○	○	○	○	○	○	○	○	○
Wissenschaftlichkeit	○	○	○	○	○	○	○	○	○	○
Praktikabilität (bei Verwendung von Routinedaten)	○	○	○	○	○	○	○	○	○	○

Bitte begründen Sie Ihre Entscheidung und geben Sie Änderungsvorschläge, wenn Sie sich für Enthaltung oder einen Skalenwert von 1-3 entschieden haben.

1 Literatur

1. SoSci Survey GmbH SoSci Survey - die Lösung für eine professionelle Onlinebefragung. https://www.soscisurvey.de/. Zugegriffen: 27 Aug 2022
2. Reiter A, Fischer B, Kötting J, Geraedts M, Jäckel WH, Döbler K (2007) QUALIFY: Ein Instrument zur Bewertung von Qualitätsindikatoren (QUALIFY--a tool for assessing quality indicators). Z Arztl Fortbild Qualitatssich 101(10):683–688. https://doi.org/10.1016/j.zgesun.2007.11.003
3. Weckmann G, Chenot J-F, Stracke S (2019) Versorgung von Patienten mit chronischer nicht-dialysepflichtiger Nierenerkrankung in der Hausarztpraxis: S3 Leitlinie. https://www.awmf.org/leitlinien/detail/ll/053-048.html. Zugegriffen: 18 Mar 2022
4. NICE National Institute for Health and Care Excellence (2014) Chronic kidney disease in adults: assessment and management: Clinical guideline. https://www.nice.org.uk/guidance/cg182. Zugegriffen: 20 Jun 2021
5. Kidney Disease Improving Global Outcomes (2013) KDIGO 2012 Clinical Practice Guideline for the Evaluation and Management of Chronic Kidney Disease. https://kdigo.org/guidelines/. Zugegriffen: 14 Oct 2022
6. NICE National Institute for Health and Care Excellence NICE Quality and Outcomes Framework indicator. https://www.nice.org.uk/standards-and-indicators/qofindicators. Zugegriffen: 06.2021
7. Tu K, Bevan L, Hunter K, Rogers J, Young J, Nesrallah G (2017) Quality indicators for the detection and management of chronic kidney disease in primary care in Canada derived from a modified Delphi panel approach. CMAJ Open 5(1):E74-E81. https://doi.org/10.9778/cmajo.20160113
8. Bundesärztekammer (BÄK), Kassenärztliche Bundesvereinigung (KBV), Arbeitsgemeinschaft der Wissenschaftlichen Medizinischen Fachgesellschaften AWMF (2010, zuletzt verändert: 2015) Nationale VersorgungsLeitlinie: Nierenerkrankungen bei Diabetes im Erwachsenenalter - Langfassung, 1. Auflage. Version 6. https://www.leitlinien.de/. Zugegriffen: 13 Oct 2022
9. Litvin CB, Ornstein SM (2014) Quality Indicators for Primary Care: An Example for Chronic Kidney Disease. J Ambul Care Manage 37(2):171–178. https://doi.org/10.1097/JAC.0000000000000015
10. Williams B, Mancia G, Spiering W et al. (2018) 2018 ESC/ESH Guidelines for the management of arterial hypertension. Eur Heart J 39(33):3021–3104. https://doi.org/10.1093/eurheartj/ehy339
11. Ludt S, Angelow A, Baum E et al. (2017) Hausärztliche Risikoberatung zur kardiovaskulären Prävention: S3-Leitlinie. https://www.awmf.org/leitlinien/detail/ll/053-024.html. Zugegriffen: 18 Mar 2022
12. Kidney Disease Improving Global Outcomes (KDIGO) Blood Pressure Work Group (2021) KDIGO 2021 Clinical Practice Guideline for the Management of Blood Pressure in Chronic Kidney Disease. Kidney Int 99:S1-S87. https://doi.org/10.1016/j.kint.2020.11.003
13. Whelton PK, Carey RM, Aronow WS et al. (2018) 2017 ACC/AHA/AAPA/ABC/ACPM/AGS/APhA/ASH/ASPC/NMA/PCNA Guideline for the Prevention, Detection, Evaluation, and Management of High Blood Pressure in Adults: A Report of the American College of Cardiology/American Heart Association Task Force on Clinical Practice Guidelines. Hypertension 71(6):e13-e115. https://doi.org/10.1161/HYP.0000000000000065

14. van den Bulck SA, Vankrunkelsven P, Goderis G et al. (2020) Developing quality indicators for Chronic Kidney Disease in primary care, extractable from the Electronic Medical Record. A Rand-modified Delphi method. BMC Nephrol 21:161. https://doi.org/10.1186/s12882-020-01788-8
15. Bello AK, Ronksley PE, Tangri N et al. (2019) Quality of Chronic Kidney Disease Management in Canadian Primary Care. JAMA Netw Open 2(9):e1910704. https://doi.org/10.1001/jamanetworkopen.2019.10704
16. Lazarus B, Wu A, Shin J-I et al. (2018) Association of Metformin Use With Risk of Lactic Acidosis Across the Range of Kidney Function: A Community-Based Cohort Study. JAMA Intern Med 178(7):903–910. https://doi.org/10.1001/jamainternmed.2018.0292
17. Fukuma S, Shimizu S, Niihata K et al. (2016) Development of quality indicators for care of chronic kidney disease in the primary care setting using electronic health data: a RAND-modified Delphi method. Clin Exp Nephrol 21:247–256. https://doi.org/10.1007/s10157-016-1274-8
18. Remschmidt C, Harder T, Wichmann O et al. (2016) Hintergrundpapier der STIKO: Evaluation der bestehenden Influenzaimpfempfehlung für Indikationsgruppen und für Senioren (Standardimpfung ab 60 Jahren). Bundesgesundheitsblatt Gesundheitsforschung Gesundheitsschutz 59:1606–1622. https://doi.org/10.1007/s00103-016-2467-8
19. Gilbertson DT, Unruh M, McBean AM, Kausz AT, Snyder JJ, Collins AJ (2003) Influenza vaccine delivery and effectiveness in end-stage renal disease. Kidney Int 63(2):738–743. https://doi.org/10.1046/j.1523-1755.2003.00787.x
20. Makani H, Bangalore S, Desouza KA, Shah A, Messerli FH (2013) Efficacy and safety of dual blockade of the renin-angiotensin system: meta-analysis of randomised trials. BMJ 346:f360. https://doi.org/10.1136/bmj.f360
21. The ONTARGET Investigators, Yusuf, S, Teo KK, Pogue J et al. (2008) Telmisartan, Ramipril, or Both in Patients at High Risk for Vascular Events. N Engl J Med 358(15):1547–1559. https://doi.org/10.1056/NEJMoa0801317
