# Supplementary material for: Development of quality indicators for the care of patients with chronic kidney disease: Results of a structured consensus process using the Delphi technique
Source: Bundesgesundheitsblatt Gesundheitsforschung Gesundheitsschutz. 2023 May 16;66(6):677–88. [Article in German] doi: 10.1007/s00103-023-03700-9 (PMC10261173; doi:10.1007/s00103-023-03700-9)
Supplement: Supplementary file 2 [file 103_2023_3700_MOESM2_ESM.pdf]

Onlinematerial 2. Relevanz, Wissenschaftlichkeit, Praktikabilität nach QUALIFY

Relevanz	Wissenschaftlichkeit	Praktikabilität
Bedeutung des mit dem QI erfassten Qualitätsmerkmal für das Versorgungssystem	Klarheit der Definitionen<br>Validität, Spezifität, Sensitivität, Reliabilität	Verständlichkeit, Interpretierbarkeit
		Beeinflussbarkeit der Indikatorausprägung
Nutzen	Indikatorevidenz	
Berücksichtigung potentieller Risiken/ Nebenwirkungen	Risikoadjustierung	Datenverfügbarkeit, Erhebungsaufwand
	Statistische Unterscheidungsfähigkeit	Implementationsbarrieren berücksichtigen
		Richtigkeit, Vollzähligkeit und Vollständigkeit der Daten kann überprüft werden

Nach Reiter et al. 2008 [2] modifiziert.

Onlinematerial 3.

Entwicklung von Qualitätsindikatoren zur leitliniengerechten Versorgung von Patienten ab 70 Jahre mit chronischer nicht-dialysepflichtiger Nierenerkrankung

Vorwort

Die vorgeschlagenen Qualitätsindikatoren sind aus Empfehlungen der S3-Leitlinie *Versorgung von Patienten mit chronischer nicht-dialysepflichtiger Nierenerkrankung in der Hausarztpraxis* [3] abgeleitet worden, sowie von bereits vorgeschlagenen Qualitätsindikatoren basierend auf internationalen Leitlinien. [4, 5]

Die Qualitätsindikatoren sind für die ambulante Versorgung von Erwachsenen mit eingeschränkter, nicht-dialysepflichtiger chronischer Nierenerkrankung in Hausarztpraxen entwickelt worden.

Nicht Gegenstand dieser Qualitätsindikatoren ist die Versorgung von (angelehnt an [3]):

- Kindern bis zum 18. Lebensjahr
- Patienten mit akuter Nierenschädigung (AKI - Acute Kidney Injury)
- Frauen während der Schwangerschaft, einschließlich Diabetes in der Schwangerschaft
- Patienten die bereits dialysepflichtig sind (ICD-Code: N18.5 - dialysepflichtige chronische Nierenerkrankung)
- Patienten nach Nierentransplantation
- stationäre Patienten
- Patienten mit einer eGFR ≥ 60 ml/min und einer isolierten Hämaturie
- Patienten mit einer eGFR ≥ 60 ml/min und einer isolierten Proteinurie/Albuminurie
- Patienten mit genetischen Erkrankungen oder Syndromen, wie z.B. Alport-Syndrom, Morbus Fabry, genetisch bedingten Zystennieren

Nachfolgend wird nur noch von chronischer Nierenerkrankung gesprochen, wobei die dialysepflichtigen chronischen Nierenerkrankungen nicht inbegriffen sind.

Die Inzidenz, als neu diagnostizierte chronische Nierenerkrankung (CKD, Erstdiagnose), wird definiert als:

$$Inzidenz = \frac{Anzahl\ \geq 70\ jährige\ Patienten\ mit\ CKD,\ ohne\ CKD\ in\ den\ letzten\ 4\ Quartalen\ vor\ Indexquartal}{Alle\ Patienten \geq 70\ Jahre\ ohne\ CKD\ in\ den\ letzten\ 4\ Quartalen\ vor\ Indexquartal}$$

Das Indexquartal bezeichnet das Quartal, in dem die diagnostizierte/verschlüsselte CKD zum ersten Mal auftritt.

Die Prävalenz, als bestehende (prävalente) CKD, wird definiert als:

$$Prävalenz\ = \frac{Anzahl\ \geq 70\ jährige\ Patienten\ mit\ diagnostizierter\ oder\ kodierter\ nicht\ dialysepflichtiger\ CKD}{Alle\ Patienten\ \geq 70\ Jahre}$$

Die Datenbasis für die Messung sind entweder Routinedaten (Abrechnungsdaten) oder/und das Chart Review (Medical Record Review oder auch Audit- Überprüfung der Krankenakte von Patienten). Soweit es inhaltlich möglich war, wurden Routinedaten bevorzugt ausgewählt. Sind sowohl Routinedaten als auch Chart Review ausgewählt, soll die Praktikabilität anhand von Routinedaten bewertet werden.

Der Betrachtungszeitraum bezieht sich im Regelfall auf 4 Quartale/12 Monate, es sei denn, es ist anders spezifiziert. Für Dauerdiagnosen muss die Verfügbarkeit von älteren Diagnosen (über die 4 Quartale hinaus) sichergestellt und berücksichtigt werden, da die Prävalenz sonst unterschätzt wird. Dauerdiagnosen werden vom Hausarzt nicht immer in jedem Quartal fortlaufend verschlüsselt.

Zu berücksichtigen ist, dass bei Nutzung von Routinedaten nur Patienten eingeschlossen werden können, die im Betrachtungszeitraum (i.d.R. 4 Quartale) durchgängig versichert waren bzw. durchgängig bei der gleichen Krankenkasse versichert waren und nicht verstorben sind. Bei der Durchführung eines Chart Reviews ist zu berücksichtigen, dass die Patienten im Betrachtungszeitraum bei demselben Arzt in Behandlung waren (z. B. Ausschluss von Patienten die gerade neu zu dem Arzt gewechselt sind) und ebenfalls nicht verstorben sind. Dies ist jedoch nur eingeschränkt möglich (Ärztehopping). Bei einem Chart Review wird lediglich eine Stichprobe (am besten eine Zufallsstichprobe) der betreffenden Patienten in den Hausarztpraxen einbezogen.

Der ICD-10 Code N18.9 - chronische Nierenkrankheit, nicht näher bezeichnet, wird grundsätzlich in die Berechnungen mit aufgenommen, da er oft für die Verschlüsselung von CKD verwendet wird und bei Ausschluss zur Unterschätzung führen würde. Patienten, die dialysepflichtig sind, sind mit sehr großer Wahrscheinlichkeit korrekt (durch den Nephrologen/die Nephrologin) verschlüsselt. Daher gehen wir davon aus, dass unter N18.9 keine dialysepflichtigen Patienten kodiert sind. Als Sensitivitätskontrolle können zusätzlich die ICD-10 Codes für Dialysebehandlung (Z49; Z99.2) und die OPS-Codes für Hämofiltration (8-853), Hämodialyse (8-854), Hämodiafiltration (8-855), Peritonealdialyse (8-857) und Nierentransplantation (5-555) berücksichtigt werden.

Der Prävalenz- sowie der Inzidenzindikator, stehen nicht zur Abstimmung, da sie oft Teil anderer Qualitätsindikatoren sind. Es sind lediglich Verbesserungsvorschläge zur Messung von Inzidenz und Prävalenz vorgesehen.

Die Beschreibung der einzelnen Indikatoren orientiert sich an der folgenden Struktur:

Allgemeiner Aufbau:

<table>
<tr><td>Nr.</td><td colspan="2">vorgeschlagener Qualitätsindikator</td></tr>
<tr><td colspan="3">Fokus: Screening, Diagnose, Medikamentöse Therapie, Monitoring</td></tr>
<tr><td colspan="3">Grundlage: Leitlinienempfehlung, bereits vorgeschlagene QI's, abgeleitet aus Literatur</td></tr>
<tr><td colspan="3">☐ Routinedaten
☐ Chart Review</td></tr>
<tr><td colspan="3">Datenquelle:
· Primärdaten (Chart Review)
· Sekundärdaten (Routinedaten)</td></tr>
<tr><td colspan="2">Zähler:</td><td></td></tr>
<tr><td colspan="2">Nenner:</td><td></td></tr>
<tr><td colspan="3">Hintergrund:</td></tr>
<tr><td colspan="3">Problem:</td></tr>
<tr><td colspan="3">Dieser QI wird auch vorgeschlagen von:</td></tr>
</table>

Diese Indikatoren (Prävalenz und Inzidenz) stehen nicht zur Abstimmung.

<table>
<tr><td>1</td><td colspan="2">Anteil der Patienten ≥70 Jahre, bei denen eine chronische nicht-dialysepflichtige Nierenerkrankung diagnostiziert wurde.</td></tr>
<tr><td colspan="3">Screening</td></tr>
<tr><td colspan="3">Prävalenzindikator (etablierte CKD)</td></tr>
<tr><td colspan="3">☒Routinedaten

☒ Chart Review</td></tr>
<tr><td colspan="3">Routinedaten

ICD-10-Code: N18

● eGFR ≥ 90 ml/min/1,73m^2 – Stadium 1 – N18.1
● eGRF ≥ 60 - < 90 ml/min/1,73m^2 – Stadium 2 – N18.2
● eGFR ≥ 30 - < 60 ml/min/1,73m^2 – Stadium 3 – N18.3
● eGFR ≥ 15 - < 30 ml/min/1,73m^2 – Stadium 4 – N18.4
● chronische Nierenkrankheit, nicht näher bezeichnet – N18.9</td></tr>
<tr><td colspan="2">Zähler:</td><td>Anzahl der ≥70-jährigen Patienten mit diagnostizierter bzw. kodierter und nicht dialysepflichtiger CKD</td></tr>
<tr><td colspan="2">Nenner:</td><td>Alle Patienten ≥70 Jahre</td></tr>
<tr><td colspan="3">Hintergrund:

DEGAM: „Chronische Nierenerkrankung ist definiert über die Nierenfunktion" [3]. Die Einteilung in Schweregrade erfolgt über die eGFR (Nierenfunktion) und Albuminurie (Nierenschaden) (KDIGO [5]). Der Qualitätsindikator liefert einen Überblick über den Anteil der mit CKD diagnostizierten Patienten in der Praxis und eine Grundlage zur Prävalenzschätzung der Erkrankung.</td></tr>
<tr><td colspan="3">Problem:

Insbesondere die frühen Stadien (eGFR > 60 ml/min/1,73m^2,) werden oft nicht ausreichend kodiert. Diese Stadien bleiben häufig auch asymptomatisch und werden deshalb in der Praxis nicht erkannt. Zudem ist eine Fehlkodierung möglich oder die Kodierung N18.9 (CKD – nicht näher bezeichnet), welche eine Einordnung in die Stadien nicht erlaubt. Durch die strikten Grenzwerte der unterschiedlichen Stadien kann außerdem durch verschiedene Messungen eine unterschiedliche Einordnung in die Stadien erfolgen.

In der Praxis ist die Überprüfung der eGFR als Grundlage für die Diagnose CKD nicht immer gegeben. In der Praxis wird häufig kein Albumin-Kreatinin-Ratio (ACR) bestimmt. Bestimmte Behandlungsempfehlungen sind an ein CKD-Stadium gebunden. In diesen Fällen wird der entsprechende ICD-Code verwendet. Bei Verschlüsselung ICD N18.5 (eGFR <15 ml/min) wird</td></tr>
</table>

angenommen, dass diese Patienten dialysepflichtig sind, auch wenn dies nicht immer der Fall ist. Diese werden daher beim Prävalenzindikator nicht berücksichtigt.<br><br>Alternativ kann die N18.5 bei Patienten ohne folgenden ICD-Codes Dialysebehandlung (Z49; Z99.2) und die OPS-Codes für Hämofiltration (8-853), Hämodialyse (8-854), Hämodiafiltration (8-855), Peritonealdialyse (8-857) und Nierentransplantation (5-555) mitberücksichtigt werden.
Dieser QI wird auch vorgeschlagen von:<br><br>● NICE QOF: „The contractor establishes and maintains a register of patients aged 18 years or over with CKD with classification of categories G3a to G5 (previously stage 3 to 5)" *(NICE id code: NM83) [6]*<br>● ICES: „The primary care providers can identify patients in their practice aged 18 years and older with chronic kidney disease." [7]

2	Anteil der Patienten ≥70 Jahre, bei denen eine chronische, nicht-dialysepflichtige Nierenerkrankung neu diagnostiziert wurde.
Screening	
Inzidenzindikator (erstmals diagnostizierte CKD)	
☒ Routinedaten<br><br>☒ Chart Review	
Routinedaten<br><br>ICD-10-Code: N18<br><br>● eGFR ≥ 90 ml/min – Stadium 1 – N18.1<br>● eGRF ≥ 60 - < 90 ml/min – Stadium 2 – N18.2<br>● eGFR ≥ 30 - < 60 ml/min – Stadium 3 – N18.3<br>● eGFR ≥ 15 - < 30 ml/min – Stadium 4 – N18.4<br>● chronische Nierenkrankheit, nicht näher bezeichnet – N18.9	
Zähler:	Anzahl der Patienten ≥70 Jahre mit neu diagnostizierter bzw. kodierter CKD, bei denen in den letzten 12 Monaten (4 Abrechnungsquartale vor Indexquartal) keine CKD diagnostiziert / kodiert wurde.
Nenner:	Alle Patienten ≥70 Jahre, bei denen in den letzten 12 Monaten (4 Abrechnungsquartale vor Indexquartal) keine CKD diagnostiziert / kodiert wurde.
Hintergrund:<br><br>Die Erstdiagnose wird definiert als erstmalige Abrechnung CKD und mindestens 4 Quartale vorher keine Verschlüsselung der CKD.	
Problem:<br><br>Eine sichere Abgrenzung zu AKI (Acute Kidney Injury) ist nicht immer möglich.	

QI aus Empfehlungen der DEGAM-Leitlinie („Versorgung von Patienten mit chronischer nicht-dialysepflichtiger Nierenerkrankung in der Hausarztpraxis“ [3]**)**

3	Anteil der Patienten ≥70 Jahre mit erstmals festgestellter eGFR <60 ml/min, bei denen für die Erstdiagnose eine zweimalige Messung der eGFR im Abstand von 3 Monaten durchgeführt wurde.
Diagnose	
„Bei Erstdiagnose einer GFR < 60 ml/min/1,73m^2, soll eine weitere Bestimmung der geschätzten glomerulären Filtrations-rate (eGFR) nach 3 Monaten durchgeführt werden um die Diagnose einer CKD zu stellen.“ (DEGAM-Empfehlung 6.1 [3], basierend auf KDIGO [5])	
☐ Routinedaten<br>☒ Chart Review	
**Chart Review**<br>Patienten mit eGFR < 60 ml/min und zweimaliger Messung der eGFR im Abstand von 3 Monaten	
Zähler:	Anzahl der Patienten ≥70 Jahre mit erstmals festgestellter eGFR <60 ml/min, bei denen im Diagnosequartal zwei eGFR-Bestimmungen durchgeführt wurden oder eine weitere Messung im Folgequartal.
Nenner:	Anzahl der Patienten ≥70 Jahre mit erstmals festgestellter eGFR <60 ml/min.
Hintergrund:<br>KDIGO: „CKD is defined as abnormalities of kidney structure or function, present for > 3 months, with implications for health." [5]. Für die Diagnose sollte eine zweite eGFR-Messung 3 Monate nach der erstmals festgestellten erniedrigten eGFR erfolgen. (KDIGO [5])	
Problem:<br>In der Praxis wird aus praktischen Gründen häufig schon bei erstmals erniedrigter eGFR die CKD-Diagnose gestellt, da eine AKI selten ist und die Einschätzung anhand der Komorbiditäten es oft erlaubt, sich ausreichend sicher festzulegen. [3]	
Dieser QI wird auch vorgeschlagen von:<br>● ICES: „Percentage of patients with an initial estimated glomerular filtration rate (eGFR) < 60 mL/min per 1.73 m^2 that is followed by a repeat test within 6 months." [7]	

<table>
<tr><td>4</td><td colspan="2">Anteil der Patienten ≥70 Jahre mit neu diagnostizierter CKD, bei denen für die Erstdiagnose eine zweimalige Messung der eGFR im Abstand von 3 Monaten durchgeführt wurde.</td></tr>
<tr><td colspan="3">Diagnose</td></tr>
<tr><td colspan="3">„Bei Erstdiagnose einer GFR < 60 ml/min/1,73m², soll eine weitere Bestimmung der geschätzten glomerulären Filtrations-rate (eGFR) nach 3 Monaten durchgeführt werden um die Diagnose einer CKD zu stellen.“ (DEGAM-Empfehlung 6.1 [3], basierend auf KDIGO [5])</td></tr>
<tr><td colspan="3">☒ Routinedaten

☐ Chart Review</td></tr>
<tr><td colspan="3">Routinedaten

● ICD-Nummern CKD: N18.1, N18.2, N18.3, N18.4, N18.9
● Abrechnung eGFR und Serumkreatininbestimmung: GOP 32066, 32067</td></tr>
<tr><td colspan="2">Zähler:</td><td>Anzahl der Patienten ≥70 Jahre mit Erstdiagnose CKD (Inzidenzindikator), bei denen im Diagnosequartal zwei eGFR-Bestimmungen durchgeführt wurden oder eine weitere Messung im Vorquartal.</td></tr>
<tr><td colspan="2">Nenner:</td><td>Anzahl der Patienten ≥70 Jahre mit Erstdiagnose CKD (Inzidenzindikator).</td></tr>
<tr><td colspan="3">Hintergrund:

KDIGO: „CKD is defined as abnormalities of kidney structure or function, present for > 3 months, with implications for health."[5]. Für die Diagnose sollte eine zweite eGFR-Messung 3 Monate nach der erstmals festgestellten erniedrigten eGFR erfolgen. (KDIGO [5]) Die Abrechnungsziffer für die Kreatinin-Messung gilt als Bestimmung der eGFR, die von Laboren meist automatisch mit angegeben wird.</td></tr>
<tr><td colspan="3">Problem:

In Routinedaten ist die Erstdiagnose eGFR < 60 ml/min nicht abbildbar, sondern nur die CKD verschlüsselt. Daher wird hier im Vorquartal nach der zweiten eGFR Bestimmung geschaut.

In der Praxis wird aus praktischen Gründen häufig schon bei erstmals erniedrigter eGFR die CKD-Diagnose gestellt, da eine AKI selten ist und die Einschätzung anhand der Komorbiditäten es oft erlaubt, sich ausreichend sicher festzulegen. [3] Aus den Routinedaten ist zudem lediglich die Abrechnung der eGFR-Messung (GOP) ersichtlich, die Überprüfung der gemessenen Laborwerte zu den beiden verschiedenen Zeitpunkten ist jedoch mit Routinedaten nicht möglich.</td></tr>
<tr><td colspan="3">Dieser QI wird auch vorgeschlagen von:</td></tr>
</table>

● ICES: "Percentage of patients with an initial estimated glomerular filtration rate (eGFR) < 60 mL/min per 1.73 m^2 that is followed by a repeat test within 6 months." [7]

5	Anteil der Patienten ≥70 Jahre mit Diabetes mellitus, bei denen mindestens einmal jährlich die eGFR bestimmt wurde.	
Screening		
„Zur Erfassung der Nierenfunktion bei Patienten mit Diabetes mellitus sollte einmal jährlich die eGFR bestimmt werden.“ (DEGAM-Empfehlung 5.2 [3])		
☒ Routinedaten<br><br>☒ Chart Review		
Routinedaten:<br><br>● Diabetes: ICD: E11.- (DM Typ 2), E10.- (DM Typ 1)<br>● Kreatininbestimmung im Serum: GOP 32066, 32067<br>● Laborausnahmeziffer (manifester Diabetes mellitus): 32022 (Glucose, Kreatinin, HbA1C, Mikroalbumin im Urin)		
Zähler:	Anzahl der Patienten ≥70 Jahre mit Diabetes mellitus (ICD E11, E10), bei denen mindestens einmal innerhalb eines Jahres eine eGFR-Bestimmung erfolgte.	
Nenner:	Anzahl der Patienten ≥70 Jahre mit Diabetes mellitus (ICD E11, E10)	
Hintergrund:<br><br>Einer der wichtigsten Ursachen der CKD ist Diabetes mellitus. [3] Deshalb sollte mindestens einmal jährlich die eGFR kontrolliert werden, um das mögliche Vorliegen einer CKD zu überprüfen. (DEGAM-Leitlinie [3], DMP Diabetes, NVL Diabetes [8])		
Dieser QI wird auch vorgeschlagen von:<br><br>● ICES: „Percentage of patients with risk factors for chronic kidney disease (diabetes, hypertension) with an eGFR in the past 18 months." [7]		

<table>
<tr><td>6</td><td colspan="2">Anteil der Patienten ≥70 Jahre mit Erstdiagnose Bluthochdruck und ohne bekannte CKD, die eine Bestimmung der eGFR erhalten haben.</td></tr>
<tr><td colspan="3">Screening</td></tr>
<tr><td colspan="3">„Patienten ohne bekannte CKD, soll bei Erstdiagnose eines Bluthochdrucks eine Serumkreatinin-Bestimmung mit eGFR und eine Urinuntersuchung auf Eiweiß oder Albumin angeboten werden." (DEGAM-Empfehlung 5.3 [3])</td></tr>
<tr><td colspan="3">☒ Routinedaten

☒ Chart Review</td></tr>
<tr><td colspan="3">Routinedaten

● Erstmals kodierter Bluthochdruck (ICD I10-I15) definiert als jeweils eine Kodierung in 2 unterschiedlichen Quartalen innerhalb von 1 Jahr (4 Quartalen) und mindestens 4 Quartale vor Indexquartal keine Verschlüsselung
● Kreatinin mittels Jaffe: 32066, enzymatisch: 32067</td></tr>
<tr><td colspan="2">Zähler:</td><td>Anzahl der Patienten ≥70 Jahre mit Erstdiagnose Bluthochdruck (ICD I10-I15, kodiert in 2 unterschiedlichen Quartalen innerhalb von 1 Jahr und mind. 4 Quartale vor Indexquartal keine Verschlüsselung), die eine Serumkreatinin-Bestimmung mit eGFR erhalten haben</td></tr>
<tr><td colspan="2">Nenner:</td><td>Anzahl der Patienten ≥70 Jahre mit Erstdiagnose Bluthochdruck (ICD I10-I15, kodiert in 2 unterschiedlichen Quartalen innerhalb von 1 Jahr und mind. 4 Quartale vor Indexquartal keine Verschlüsselung)</td></tr>
<tr><td colspan="3">Hintergrund:

Der Bluthochdruck ist einer der wichtigsten Ursachen für die Entstehung der CKD. (DEGAM-Leitlinie [3]) Patienten mit Hypertonie haben ein erhöhtes Risiko, an einer CKD zu erkranken. [3] Das absolute Risiko, an einer terminalen Nierenerkrankung zu erkranken, ist jedoch gering ([3]).

Die DEGAM-Leitlinie empfiehlt bei Erstdiagnose eine Überprüfung der Nierenfunktion, aber keine „[...] routinemäßige Untersuchung der Nierenfunktion und Proteinurie, wenn eine Nierenerkrankung initial ausgeschlossen wurde [...]" [3]</td></tr>
<tr><td colspan="3">Dieser QI wird auch empfohlen von:

● ICES: „Percentage of patients with risk factors for chronic kidney disease (diabetes, hypertension) with an eGFR in the past 18 months." [7]</td></tr>
</table>

7	Anteil der Patienten ≥70 Jahre mit Erstdiagnose Bluthochdruck und ohne bekannte CKD, die eine Urinuntersuchung auf Protein oder Albumin erhalten haben.
Screening	
„Patienten ohne bekannte CKD, soll bei Erstdiagnose eines Bluthochdrucks eine Serumkreatinin-Bestimmung mit eGFR und eine Urinuntersuchung auf Eiweiß oder Albumin angeboten werden." (DEGAM-Empfehlung 5.3 [3])	
☒ Routinedaten<br>☒ Chart Review	
Routinedaten<br>● Erstmals kodierter Bluthochdruck (ICD I10-I15) definiert als jeweils eine Kodierung in 2 unterschiedlichen Quartalen innerhalb von 1 Jahr (4 Quartalen) und mindestens 4 Quartale vor Indexquartal keine Verschlüsselung<br>● GOP 32435 Albuminbestimmung, Kreatinin mittels Jaffe: 32066 oder enzymatisch: 32067	
Zähler:	Anzahl der Patienten ≥70 Jahre mit Erstdiagnose Bluthochdruck (ICD I10-I15, kodiert in 2 unterschiedlichen Quartalen innerhalb von 1 Jahr und mind. 4 Quartale vor Indexquartal keine Verschlüsselung) die eine Urinuntersuchung auf Eiweiß oder Albumin erhalten haben.
Nenner:	Anzahl der Patienten ≥70 Jahre mit Erstdiagnose Bluthochdruck (ICD I10-I15, kodiert in 2 unterschiedlichen Quartalen innerhalb von 1 Jahr und mind. 4 Quartale vor Indexquartal keine Verschlüsselung).
Hintergrund:<br>Der Bluthochdruck ist einer der wichtigsten Ursachen für die Entstehung der CKD. Patienten mit Hypertonie haben ein erhöhtes Risiko, an einer CKD zu erkranken. [3] Das absolute Risiko, an einer terminalen Nierenerkrankung zu erkranken, ist jedoch gering. (DEGAM-Leitlinie [3])<br>Die DEGAM-Leitlinie empfiehlt bei Erstdiagnose eine Überprüfung der Nierenfunktion, aber keine „[...] routinemäßige Untersuchung der Nierenfunktion und Proteinurie, wenn eine Nierenerkrankung initial ausgeschlossen wurde [...]" [3]	
Literatur:<br>„Screening for proteinuria annually in patients with diabetes and/or hypertension“ [9]	

<table>
<tr><td>8</td><td>Anteil der Patienten ≥70 Jahre mit Erstdiagnose CKD (Inzidenzindikator), bei denen eine Bestimmung der Albumin-Kreatinin-Ratio im Urin erfolgte.</td></tr>
<tr><td colspan="2">Diagnose</td></tr>
<tr><td colspan="2">„Bei Erstdiagnose der CKD (GFR < 60 ml/min) soll eine Bestimmung der Albumin-Kreatinin Ratio im Urin erfolgen.“ (DEGAM-Empfehlung 6.3 [3])</td></tr>
<tr><td colspan="2">☒ Routinedaten

☒ Chart Review</td></tr>
<tr><td colspan="2">Routinedaten:

Chronische Nierenerkrankung

● ICD: N18.1, N18.2, N18.3, N18.4, N18.9 (nicht näher klassifiziert)

Bestimmung der ACR mittels Spontanurinprobe (spot urine) oder 24-h-Sammelurin

● Kreatinin GOP 32066, 32067
● Albumin GOP 32435

(beides muss gleichzeitig abgerechnet worden sein.)</td></tr>
<tr><td>Zähler:</td><td>Anzahl der Patienten ≥70 Jahre mit Erstdiagnose CKD (Inzidenzindikator) und mindestens einer Bestimmung der Albumin-Kreatinin-Ratio im Urin</td></tr>
<tr><td>Nenner:</td><td>Anzahl der Patienten ≥70 Jahre mit Erstdiagnose CKD (Inzidenzindikator)</td></tr>
<tr><td colspan="2">Hintergrund:

Die Proteinurie ist ein weiterer "wichtiger prognostischer Marker" (DEGAM-Leitlinie [3]) für die chronische Nierenerkrankung. Die Albumin-Kreatinin-Ratio (ACR) wird mit aus dem Urin gemessenen Kreatinin- und Albuminwerten bestimmt. [3] Mittels ACR kann die CKD in ein Stadium A1 (<30 mg/g), A2 (30-300 mg/g) und A3 (>300 mg/g) eingeteilt werden. [3, 5] Zur Bestimmung der Werte wird vorwiegend Spontanurin benutzt, welcher fehleranfällig sein kann. ([3])

Abweichend von der DEGAM Empfehlung wird hier auch CKD-Stadium 1 und 2 mitberücksichtigt. Auch bei der Diagnose einer CKD bei eGFR >60 ml/min ist eine Messung der Proteinurie zur Beurteilung der Nierenfunktion notwendig.</td></tr>
<tr><td colspan="2">Problem:

Kreatinin und Albumin müssen zur gleichen Zeit abgerechnet worden sein. Das Albumin-Kreatinin-Ratio wird durch das Labor nur dann berechnet, wenn beide Parameter abgerechnet worden sind.</td></tr>
<tr><td colspan="2">Dieser QI wird auch vorgeschlagen von:</td></tr>
</table>

- ICES: „Percentage of patients with an initial eGFR < 60 mL/min/1.73 m^2 with an albumin–creatinine ratio (ACR) test conducted within 6 months." [7]

9	Anteil der Patienten ≥70 Jahre mit Erstdiagnose CKD (Inzidenzindikator), bei denen eine Urinuntersuchung mittels Streifentest erfolgte.
Diagnose	
„Bei Erstdiagnose der CKD (GFR < 60 ml/min/1,73m²) soll eine Bestimmung der Hämaturie mit einem Streifentest im Urin erfolgen.“ (DEGAM-Empfehlung 6.4 [3])	
☒ Routinedaten<br><br>☒ Chart Review	
Routinedaten:<br><br>● Chronische Nierenerkrankung: ICD N18.1, N18.2, N18.3, N18.4, N18.9 (nicht näher bezeichnet)<br>● Abrechnung Harnstreifentest: GOP 32033	
Zähler:	Anzahl der Patienten ≥70 Jahre mit Erstdiagnose CKD (Inzidenzindikator), die eine Urinuntersuchung mittels Streifentest erhalten haben
Nenner:	Anzahl der Patienten ≥70 Jahre mit Erstdiagnose CKD (Inzidenzindikator)
Hintergrund:<br><br>Mittels Streifentest soll auf eine nicht-sichtbare Hämaturie untersucht werden, welche beispielsweise auf eine Glomerulonephritis hinweisen kann (DEGAM-Leitlinie [3]).	

10	Anteil der Patienten ≥70 Jahre mit Erstdiagnose CKD (GFR < 60 ml/min/1,73 m^2), bei denen in den letzten 12 Monaten (4 Quartale vor Indexquartal) keine CKD diagnostiziert/kodiert wurde, bei denen eine Kontrolle des Blutdrucks erfolgte.
Diagnose	
„Bei Erstdiagnose einer CKD (GFR < 60 ml/min/1,73m2) soll der Blutdruck kontrolliert werden." (DEGAM-Empfehlung 6.6 [3])	
☐ Routinedaten<br><br>☒ Chart Review	
Chart Review	
Zähler:	Anzahl der Patienten ≥70 Jahre mit Erstdiagnose CKD (eGFR < 60 ml/min/1,73 m^2, Inzidenzindikator), bei denen eine Blutdruckmessung in der Praxis durchgeführt wurde.
Nenner:	Anzahl der Patienten ≥70 Jahre mit Erstdiagnose CKD (eGFR < 60 ml/min/1,73 m^2, Inzidenzindikator)
Hintergrund:<br><br>Da ein Bluthochdruck Ursache und auch Folge einer chronischen Nierenerkrankung sein kann, wird die Messung des Blutdrucks bei Erstdiagnose einer CKD empfohlen (DEGAM-Leitlinie [3], KDIGO [5]).	
Problem:<br><br>Da die Blutdruckmessung Teil der Ordinationspauschale ist und ihre Durchführung daher nicht aus Routinedaten erfasst werden kann, ist ein Chart Review zur Überprüfung des Qualitätsindikators notwendig. Voraussetzung dafür ist eine Dokumentation des eGFR-Werts und der erfolgten Blutdruckmessung in der Praxis.	

11	Anteil der Patienten ≥70 Jahre mit CKD (<60 ml/min/1,73 m^2) aber unter 80 Jahre, deren systolischer Blutdruck auf <140 mmHg eingestellt ist.

Medikamentöse Therapie

„Allen Patienten mit CKD (GFR < 60 ml/min/1,73m^2) und Blutdruck > 140/90 mmHg sollten Maßnahmen zur Blutdrucksenkung angeboten werden. Der Zielwert sollte individuell festgelegt werden.“ (DEGAM-Empfehlung 7.8 [3])

☐ Routinedaten

☒ Chart Review

Chart Review

- Daten über eGFR-Wert
- Daten über Blutdruck (Angaben über systolischen und diastolischen Blutdruck)

Zähler:	Anzahl der Patienten ≥70 Jahre und <80 Jahre mit CKD und mit einem systolischen Blutdruck unter 140 mmHg
Nenner:	Anzahl der Patienten ≥70 Jahre und <80 Jahre mit CKD

Hintergrund:

Hypertonie gilt sowohl als Ursache als auch als Folge einer Chronischen Nierenerkrankung, zudem ist es einer der häufigsten Gründe für eine Dialysepflicht im Verlauf der Erkrankung (DEGAM [3]). Als Bluthochdruck wird nach ESC/ESH [10]) das Vorliegen eines systolischen Werts ≥ 140 mmHg und/oder eines diastolischen Werts ≥90 mmHg definiert. Abhängig von weiteren Co-Erkrankungen und Risikofaktoren (Diabetes mellitus, Albuminurie) gibt es verschiedene Zielbereiche für die Patienten. Allgemein sollte ein Blutdruck unterhalb von 140/90 mmHg erreicht werden. [11] Es gibt verschiedene blutdrucksenkende Therapien (Lebensstilumstellung, medikamentöse Einstellung) (DEGAM-Leitlinie „Hausärztliche Risikoberatung zur kardiovaskulären Prävention“ [11]). Die Leitlinienempfehlung (s.o., [3]) wurde für den Qualitätsindikator umformuliert, da nicht jedem Patienten mit einem Blutdruck > 140/90 mmHg medikamentöse Maßnahmen empfohlen werden. Beispielsweise kann bei entsprechendem Alter und kardiovaskulärem Risiko eine Lebensstilumstellung versucht werden, welche als solche nicht als Leistung abgerechnet werden kann.

Sowohl der systolische als auch der diastolische Wert sind Prognosefaktoren für kardiovaskuläre Events. Der systolische Wert scheint im höheren Alter eine höhere Bedeutung als Risikofaktor zu haben (ESC/ESH 2018, [10]). Die Leitlinie „KDIGO Clinical practice guideline for the Management of Blood Pressure in Chronic Kidney Disease“ [12] legt in ihren Empfehlungen nur einen Zielbereich für den systolischen Wert (<120 mmHg) fest. Dies wird damit begründet, dass aus erniedrigten systolischen Werten häufig auch erniedrigte diastolische Werte resultieren und dass es wenige RCTs gibt, die diastolische Werte in Relation mit klinischen Outcomes gesetzt haben, besonders im Zusammenhang mit CKD. [12] Einige Leitlinien (NICE [4]) und Literaturquellen (siehe unten)

empfehlen ein Blutdruckziel von 130/80 mmHg (SPB von 120-129 mmHg) für Patienten mit CKD und Diabetes oder CKD und Proteinurie/Albuminurie (ACR > 300 mg/g). Bei älteren Patienten spielen Komorbiditäten und Arzneimittelinteraktionen mit Antihypertensiva eine wichtige Rolle. [10] Bei manchen Patienten liegt der kleinstmöglich erreichbare Blutdruckwert höher als eigentlich empfohlen. [10] Aber auch in dieser Patientengruppe werden möglichst niedrige Blutdruckzielwerte empfohlen, da das kardiovaskuläre Risiko gesenkt werden kann (ESC / ESH 2018, [10]). Die Studienlage zur Blutdrucktherapie älterer Patienten ist jedoch unzureichend, sodass über die Therapie individuell entschieden werden sollte (DEGAM-Leitlinie „Risikoberatung zur kardiovaskulären Prävention" [11]). Die amerikanischen Leitlinien (ACC/AHA [13]) empfehlen auch bei älteren Pat. <130 mmHg. Bei den Europäischen Leitlinien (ESH/ESC [10]) sind die Zielwerte bei älteren Pat. höher (<140/90 mmHg) angegeben. Obwohl viele andere Leitlinien sowohl für den systolischen als auch für den diastolischen Wert einen Zielbereich definieren, ist es in der Praxis praktikabler, sich nur am systolischen Wert zu orientieren. Die Begrenzung auf Patienten unter 80 Jahren erfolgt, weil bei Menschen mit begrenzter Lebenserwartung (ab 80 Jahren) eine individualisierte Therapieentscheidung getroffen werden sollte.

Problem:

Es gibt kein festgelegtes Befunddatenfeld für Blutdruck in den in Deutschland zugelassenen Praxisverwaltungssystemen. Deshalb ist ein automatisiertes Auslesen der Blutdruckwerte für eine Analyse der Praxisdaten nicht möglich.

Dieser QI wird auch vorgeschlagen von:

- NICE QOF: "The percentage of patients on the CKD register in whom the last blood pressure reading (measured in the preceding 12 months) is 140/90 mmHg or less" *(NICE id code NM117) [6]*
- ICES: "Percentage of patients with chronic kidney disease with a most recent blood pressure < 140/90 mm Hg, or with chronic kidney disease and diabetes with a most recent blood pressure < 130/80 mm Hg." [7]

Literaturbeispiele (Blutdruckziel 130/80 mmHg):

„Percentage of patients with CKD with

- proteinuria ≥1 g/day or

- diabetes mellitus or

- strongly increased albuminuria (ACR > 300 mg/g creatinine or >300 mg/24 hours) or

- ACR ≥700 mg/g

in whom the target BP of < 130/80 mmHg (SBP range 120 - 129 mmHg) is archieved by using the first choice antihypertensive drugs (ACE inhibitors or ARBs)" [14]

„Most recent blood pressure < 130/80 mmHg for patients with CKD *with* proteinuria

Proteinuria defined as albumin to creatinine ratio > 300 mg/g or >300 mg of albumin in the urine per 24 h or protein to creatinine ratio > 0.3 mg/g" [9]<br><br>"Patients with an eGFR <60 mL/min per 1.73 m^2 who also had evidence of albuminuria and/or diabetes and achieved a target BP of ≤130/80 mm Hg" [15]<br><br>NICE: "In people with CKD and diabetes, and also in people with an ACR of 70 mg/mmol or more, aim to keep the systolic blood pressure below 130 mmHg (target range 120–129 mmHg) and the diastolic blood pressure below 80 mmHg. [2008]" [4]

<table>
<tr><td>12</td><td>Anteil der Patienten ≥70 Jahre mit CKD und eGFR < 30 ml/min/1,73 m^2, bei denen NSAR verordnet wurden.</td></tr>
<tr><td colspan="2">Medikamentöse Therapie</td></tr>
<tr><td colspan="2">„Nicht-steroide Antirheumatika (NSAR) sollten bei Patienten mit einer CKD bis zu einer GFR ≥30 ml/min/1,73m^2 möglichst vermieden werden oder nur so kurz und niedrig dosiert wie möglich eingesetzt werden. Ab einer GFR < 30 sind NSAR kontraindiziert.“ (DEGAM-Empfehlung 7.17 [3])</td></tr>
<tr><td colspan="2">☒ Routinedaten

☒ Chart Review</td></tr>
<tr><td colspan="2">Routinedaten:
CKD-Stadium 4 (eGFR < 30ml/min/1,73 m^2): ICD-10 N18.4
N18.9 (nicht näher klassifiziert)
Keine der folgenden ATC-Codes NSAR: M01A (ASS als Thrombozytenhemmer → B01AC06, Analgetikum extra → N02BA01)
Chart Review:
Angabe des eGFR-Werts
Angabe von Verordnung von ASS, Diclofenac, Ibuprofen, Coxibe</td></tr>
<tr><td>Zähler:</td><td>Anzahl der Patienten ≥70 Jahre mit CKD und eGFR < 30 ml/min/1,73 m^2, die in den letzten 4 Quartalen NSAR verordnet bekommen haben.</td></tr>
<tr><td>Nenner:</td><td>Anzahl der Patienten ≥70 Jahre mit CKD und eGFR < 30 ml/min/1,73 m^2 in den letzten 4 Quartalen</td></tr>
<tr><td colspan="2">Hintergrund:

NSAR sollen bei Patienten mit Chronischer Nierenerkrankung vermieden werden. Sie können unter anderem eine akute Nierenschädigung auslösen. [3] Ab einer eGFR < 30 ml/min/1,73 m^2 sind sie laut Fachinformation kontraindiziert (DEGAM-Leitlinie [3]).

Eine Alternative des Qualitätsindikators könnte „Anteil der Patienten mit CKD, bei denen NSAR verordnet wurden“ sein, da auch in den früheren Stadien die NSAR-Verordnung vermieden werden soll.</td></tr>
<tr><td colspan="2">Problem:

Für die Anwendung des Qualitätsindikators mithilfe von Routinedaten ist die genaue Verschlüsselung des CKD-Stadiums (ICD N 18.4, eGFR < 30 ml/min/1,73 m^2) notwendig. Ein Teil der Patienten wird mit ICD N 18.9 verschlüsselt, was zu einer Überschätzung der Versorgungsqualität führen kann. Es handelt sich hier lediglich um die verordneten NSAR. Frei verkäufliche NSAR werden mittels Routinedaten nicht erfasst und können daher nicht berücksichtigt werden. N18.9</td></tr>
</table>

überschätzt möglicherweise Patienten mit Kontraindikation. Es lässt sich nicht ausschließen, ob es sich dabei um partizipative Entscheidung/Patientenwunsch nach Aufklärung handelt.<br><br>Der Indikator sollte optimalerweise bei 0% liegen.
Dieser QI wird auch empfohlen von:<br>● ICES: „Percentage of patients with stage 3–5 chronic kidney disease and a prescription for a nonsteroidal anti-inflammatory drug for more than 2 weeks." [7]

13	Anteil der Patienten ≥70 Jahre mit CKD, deren eGFR entsprechend der Monitoringintervalle (abhängig vom eGFR- und Albuminuriewert) kontrolliert wurde. (siehe Abbildung S3-Leitlinie CKD [3], S. 87, Abb. 2, adaptiert nach [4])

Monitoring

„Patienten mit CKD mit und ohne Diabetes sollten in regelmäßigen Abständen entsprechend CKD-Stadien (s. Abb. 2) eine Bestimmung des eGFR aus Serumkreatinin angeboten werden.“ (DEGAM-Empfehlung 8.2 [3])

☐ Routinedaten

☒ Chart Review

Chart Review

Zähler:	Anzahl der Patienten ≥70 Jahre mit CKD, deren eGFR-Wert entsprechend der Monitoringintervalle kontrolliert wurde
Nenner:	Anzahl der Patienten ≥70 Jahre mit CKD

Hintergrund:

Die eGFR bei Patienten mit CKD sollte regelmäßig kontrolliert werden. [3] In Abhängigkeit vom Stadium der CKD (abhängig von eGFR und Albuminurie) sowie Komorbiditäten, Allgemeinzustand und Wunsch des Patienten sollten die Intervalle mit dem Patienten gemeinsam abgestimmt werden. (DEGAM-Leitlinie [3], NICE [4])

Problem:

Der ACR-Wert ist in Routinedaten nicht verfügbar. Dies ist jedoch wichtig für die vorgeschlagene Einordnung in die Monitoringintervalle. Daher wird hier die Umsetzung mittels Chart Review festgelegt.

Dieser QI wird auch empfohlen von:

- ICES: „Percentage of patients with chronic kidney disease with an eGFR in the past 18 months." [7]

14	Anteil der Patienten ≥70 Jahre mit CKD, deren eGFR<br><br>bei CKD-Stadium 1-3 oder unspezifisch kodiert mind. 1x im Jahr und CKD-Stadium 4 mind. 2x im Jahr kontrolliert wurde.<br><br>(Unabhängig vom Albuminuriewert, der in Routinedaten nicht zur Verfügung steht.)
Monitoring	
„Patienten mit CKD mit und ohne Diabetes sollten in regelmäßigen Abständen entsprechend CKD-Stadien (s. Abb. 2) eine Bestimmung des eGFR aus Serumkreatinin angeboten werden.“ (DEGAM-Empfehlung 8.2 [3])	
☒ Routinedaten<br><br>☐ Chart Review	
Routinedaten<br><br>ICD-10-Code:<br><br>● CKD-Stadien: N18.1, N18.2, N18.3, N18.9 à eGFR-Kontrolle mind. 1x /Jahr<br>● CKD-Stadium: N18.4 à eGFR-Kontrolle mind. 2x/Jahr<br><br>Abrechnung eGFR und Serumkreatininbestimmung: GOP 32066, 32067	
Zähler:	Anzahl der Patienten ≥70 Jahre mit CKD, deren eGFR-Wert entsprechend ihres CKD-Stadiums kontrolliert wurde.
Nenner:	Anzahl der Patienten ≥70 Jahre mit CKD
Hintergrund:<br><br>Die eGFR bei Patienten mit CKD sollte regelmäßig kontrolliert werden [3]. Die Kontrollintervalle sollen abhängig vom CKD-Stadium, aber unabhängig vom Albuminuriewert erfolgen. Dies ist eine Vereinfachung des vorherigen Qualitätsindikators, da der Albuminuriewert in Routinedaten nicht verfügbar ist. So kann dieser Qualitätsindikator mittels Routinedaten umgesetzt werden.	
Problem:<br><br>Es handelt sich um eine pragmatische Vergröberung.	
Dieser QI wird auch empfohlen von:	

- ICES: „Percentage of patients with chronic kidney disease with an eGFR in the past 18 months." [7]

<table>
<tr><td>15</td><td colspan="2">Anteil der Patienten ≥70 Jahre mit CKD, bei denen mindestens einmal jährlich der Blutdruck dokumentiert wurde.</td></tr>
<tr><td colspan="3">Monitoring</td></tr>
<tr><td colspan="3">„Patienten mit CKD sollte in regelmäßigen Abständen mindestens entsprechend der individuell vereinbarten Monitoringintervalle (s. Abb. 2) eine Messung des Blutdrucks angeboten werden.“ (DEGAM-Empfehlung 8.3 [3])</td></tr>
<tr><td colspan="3">☐ Routinedaten

☒ Chart Review</td></tr>
<tr><td colspan="3">Chart Review:

Patienten mit CKD;

Blutdruckmessung in der Praxisverwaltungssoftware oder Papierakte</td></tr>
<tr><td colspan="2">Zähler:</td><td>Anzahl der Patienten ≥70 Jahre mit CKD, deren Blutdruck einmal jährlich gemessen wurde.</td></tr>
<tr><td colspan="2">Nenner:</td><td>Anzahl der Patienten ≥70 Jahre mit CKD</td></tr>
<tr><td colspan="3">Hintergrund:

Bluthochdruck kann Ursache und auch Folge der Chronischen Nierenerkrankung sein und die Progression der Erkrankung beeinflussen. (DEGAM-Leitlinie [3])</td></tr>
<tr><td colspan="3">Problem:

Da die Blutdruckmessung Teil der Ordinationspauschale ist, kann ihre Durchführung nicht aus Routinedaten erfasst werden. Für den Qualitätsindikator eignet sich das Chart Review. Voraussetzung dafür ist die Dokumentation der erfolgten Blutdruckmessung in der Praxis.

In dem Vorschlag wird nur die Messung, aber nicht die Höhe des Blutdrucks berücksichtigt.</td></tr>
<tr><td colspan="3">Dieser QI wird auch empfohlen von:

● ICES: „Percentage of patients with chronic kidney disease with a blood pressure recorded in the past 18 months." [7]
● ICES: „Percentage of patients with diabetes and albuminuria (moderately or severely increased ACR ≥ 3 mg/mmol) with a blood pressure recorded in the past 9 months." [7]</td></tr>
</table>

16	Anteil der Patienten ≥70 Jahre mit CKD und nachgewiesener Proteinurie, deren Albumin-Kreatinin-Ratio mindestens einmal jährlich gemessen wurde.

Monitoring

„Patienten mit CKD und einer nachgewiesenen Proteinurie sollte in regelmäßigen Abständen

mindestens entsprechend der individuell vereinbarten Monitoringintervalle (siehe Abbildung 2) eine Bestimmung der Albumin-Kreatinin-Ratio angeboten werden." (DEGAM-Empfehlung 8.5 [3])

☒ Routinedaten

☒ Chart Review

Routinedaten:

Chronische Nierenerkrankung

- ICD: N18.1, N18.2, N18.3, N18.4, N18.9 (nicht näher bezeichnet)

Proteinurie

- ICD: N06

Bestimmung der ACR mittels Spontanurinprobe (spot urine) oder 24-h-Sammelurin

- Kreatinin GOP 32066, 32067
- Albumin GOP 32435

  (beides muss zur gleichen Zeit abgerechnet worden sein, da es keine GOP für ACR gibt.)

Zähler:	Anzahl der Patienten ≥70 Jahre mit CKD und nachgewiesener Proteinurie, bei denen mindestens einmal jährlich die Kreatinin-/Albumin-Ratio gemessen wurde
Nenner:	Anzahl der Patienten ≥70 Jahre mit CKD und nachgewiesener Proteinurie

Hintergrund:

DEGAM: „Zur Evaluation der Nierenfunktion ist neben der eGFR die Proteinurie ein wichtiger prognostischer Marker." [3]. Die NICE-Leitlinie empfiehlt die Bestimmung der ACR zusammen mit der eGFR [4]. Die KDIGO empfiehlt die Messung mindestens einmal jährlich [5]. Der Nutzen einer regelmäßigen Kontrolle der Proteinurie bei initial ausgeschlossener Proteinurie ist nicht belegt. (DEGAM-Leitlinie [3])

Problem:

Es existiert keine eindeutige GOP, mit der die ACR abgerechnet werden kann. Das Labor ermittelt ACR nur, wenn Albumin und Kreatinin abgerechnet worden sind. Proteinurie wird sehr wahrscheinlich schlecht verschlüsselt.

Dieser QI wird auch vorgeschlagen von:<br><br>- NICE QOF: „The percentage of patients on the CKD register whose notes have a record of a urine albumin:creatinine ratio (or protein:creatinine ratio) test in the preceding 12 months" *(NICE ID code NM109) [6]*<br>- ICES: „Percentage of patients with risk factors for chronic kidney disease (diabetes, hypertension) with an ACR in the past 18 months." [7]<br>- ICES: „Percentage of patients with chronic kidney disease with an ACR in the past 18 months." [7]
Literatur:<br><br>„Percentage of patients with CKD, in whom the GFR, albuminuria and total protein is determined at least once a year“[14]<br><br>„Monitoring for proteinuria annually in patients with eGFR < 60 (preferred screening tests include<br><br>albumin-specific dipstick or spot albumin-to-creatinine ratio“ [9]<br><br>KDIGO: „Assess GFR and albuminuria at least annually in people with CKD. Assess GFR and albuminuria more often for individuals at higher risk of progression, and/or where measurement will impact therapeutic decisions (see figure below).“ [5]

<table>
<tr><td>17</td><td colspan="2">Anteil der Patienten ≥70 Jahre mit CKD und ohne bekannte Anämie, bei denen einmal jährlich (Stadium 3) oder halbjährlich (Stadium 4) der Hämoglobinwert bestimmt wurde.</td></tr>
<tr><td colspan="3">Monitoring</td></tr>
<tr><td colspan="3">„Der Serumhämoglobinwert (Hb) sollte bei Patienten ohne bekannte Anämie mit

● CKD-Stadium G3b einmal jährlich
● CKD-Stadium G4-5 halbjährlich

bestimmt werden, um eine behandlungsbedürftige Anämie auszuschließen.“ (DEGAM-Empfehlung 8.7 [3])</td></tr>
<tr><td colspan="3">☒ Routinedaten

☒ Chart Review</td></tr>
<tr><td colspan="3">Chart Review

Routinedaten:

● ICD N18.3 (eGFR < 60 ml/min/1,73 m^2), N18.4 (eGFR < 30 ml/min/1,73 m^2)
● GOP 32120 (mechanisiertes Blutbild), 32038 (Hämoglobin einzeln)</td></tr>
<tr><td colspan="2">Zähler:</td><td>Anzahl der Patienten ≥70 Jahre mit CKD-Stadium 3 oder 4, bei denen 1x jährlich (eGFR <60 ml/min/1,73 m^2) bzw. 2x jährlich (eGFR < 30 ml/min/1,73 m^2) der Hämoglobinwert im Blut bestimmt wurde.</td></tr>
<tr><td colspan="2">Nenner:</td><td>Anzahl der Patienten ≥70 Jahre mit CKD-Stadium 3 oder 4</td></tr>
<tr><td colspan="3">Hintergrund:

Die Anämie ist eine Komplikation der CKD, vor allem in den höheren Stadien. Wichtige Ursachen sind der Mangel an Erythropoetin sowie eine "Störung des Eisenhaushalts". (DEGAM [3])

Es wird eine jährliche Kontrolle des Hb empfohlen, aufgrund des hohen Anteils von Patienten ohne Anämie im Stadium 3a aber erst ab Stadium 3b (eGFR: 44 -30 ml/min/1,73 m^2), und halbjährlich bei einer eGFR < 30 ml/min/1,73 m^2. (DEGAM-Leitlinie [3]). Da sich das Stadium 3b nicht in den Routinedaten ableiten lässt, wurde der Indikator auf Stadium 3 vergröbert.</td></tr>
<tr><td colspan="3">Problem:

Da sich das Stadium 3b nicht von den ICD-Codes ableiten lässt (ICD N18.3: eGFR 59-30 ml/min/1,73 m^2), kann die Verwertung von Routinedaten ungenau sein. Eine genaue Überprüfung ab Stadium 3b lässt sich nur mittels Chart Review durchführen.</td></tr>
</table>

18	Anteil der Patienten ≥70 Jahre mit CKD und erstmals festgestellter eGFR <30 ml/min/1,73 m^2, die an einen Nephrologen überwiesen wurden.
Diagnose	
„Bei jeder erstmals festgestellten CKD mit eGFR < 30 ml/min sollte eine Überweisung in die Nephrologie zu einer Konsiliaruntersuchung angeboten werden.“ (DEGAM-Empfehlung 6.9 [3])	
☒ Routinedaten<br><br>☒ Chart Review	
Routinedaten:<br><br>● ICD-Code N18.4, bei denen N18.4 in den letzten 12 Monaten (4 Quartalen) nicht verschlüsselt wurde<br>● Überweisung an einen Nephrologen (→ GOP 13592 Grundpauschale ab dem 60. Lebensjahr)<br><br>Chartreview:<br><br>● Angabe eGFR-Wert<br>● Überweisung an einen Nephrologen	
Zähler:	Anzahl der Patienten ≥70 Jahre mit CKD und erstmals festgestellter eGFR < 30 ml/min/1,73 m^2 (keine eGFR <30 ml/min in den letzten 4 Quartalen), die an einen Nephrologen überwiesen wurden
Nenner:	Anzahl der Patienten ≥70 Jahre mit CKD und erstmals festgestellter eGFR < 30 ml/min/1,73 m^2
Hintergrund:<br><br>DEGAM: „Die Überweisung dient dem Erkennen einer potentiell behandelbaren und reversiblen Ursache der Nierenfunktionsstörung und der Abschätzung der Notwendigkeit einer Vorbereitung zur Dialyse oder Transplantation". [3] Bei der Indikationsstellung sollen "Komorbidität, [...] Lebenserwartung und individueller Gesundheitsziele" (DEGAM-Leitlinie [3]) der Patienten berücksichtigt werden.<br><br>Bei jüngeren Patienten mit CKD sollte die Überweisungsindikation an die Nephrologie großzügiger gestellt werden. [3] DEGAM: „Bei älteren Patienten (> 70 Jahre) sollte die Indikation zur Überweisung unter Berücksichtigung der Komorbidität, der Lebenserwartung und individueller Gesundheitsziele gestellt werden." [3] Somit wird für den Indikator nicht erwartet, dass jeder Patient mit einer eGFR unter 30 ml/min überwiesen wird, sondern berücksichtigt, dass es ältere multimorbide Patienten gibt, für die eine Überweisung keinen wesentlichen Vorteil bringt.<br><br>Andere Leitlinien (KDIGO [5], NICE [4]) und Literaturquellen [14] geben weitere Empfehlungen zur Überweisungsindikation, wie beispielsweise bei einer Albuminurie (ACR ≥ 300 mg/g).	

Problem:<br><br>In Routinedaten ist die Überweisung zum Nephrologen nur dann ersichtlich, wenn der Patient sich dort tatsächlich vorgestellt hat. Eine erfolgte Überweisung vom Hausarzt, ohne dass der Patient zum Nephrologen gegangen ist, ist nicht überprüfbar. Das unter- oder überschätzt die Leitlinienadhärenz.
Dieser QI wird auch empfohlen von:<br><br>● ICES: „Percentage of patients age < 80 years with a referral to a nephrologist for eGFR < 30 mL/min per 1.73 m^2." [7]
„Percentage of patients with a GFR <30 ml/min/1,73 m^2, who are referred to a specialist“ [14]<br><br>„Referral to a nephrologist for eGFR < 30“ [9]<br><br>„Percentage of patients with a confirmed significant albuminuria (ACR ≥300 mg/g or AER ≥ 300 mg/ 24 hours), who are referred to a specialist“ [14]<br><br>„Percentage of patients with hematuria in combination with proteinuria (urine protein ≥0,5 g/day or PCR ≥500mg/g or ACR ≥ 300 mg/g creatinin), who are referred to a specialist“ [14]<br><br>KDIGO: „We recommend referral to specialist kidney care services for people with CKD in the following circumstances (*1B*):<br><br>[...]<br><br>a consistent finding of significant albuminuria (ACR ≥300 mg/g [≥30 mg/mmol] or AER ≥300 mg/24 hours, approximately equivalent to PCR ≥500 mg/g [≥50 mg/mmol] or PER ≥500 mg/24 hours); […]“ [5]

19	Anteil der Patienten ≥70 Jahre mit CKD und eGFR < 30 ml/min/1,73 m^2 und Diabetes Typ 2, bei denen Metformin verordnet wurde.

Medikamentöse Therapie

„Metformin sollte bei CKD und Diabetes Typ 2 bis zu einer eGFR ≥30 ml/min /1,73m^2 bevorzugt angeboten werden, wenn der HbA1c nicht im individuellen Zielbereich liegt.“ (DEGAM-Empfehlung 7.9 [3])

☒ Routinedaten

☒ Chart Review

Routinedaten:

Diabetes ICD-Code:

- ICD: E11 (DM Typ 2)

CKD ICD-Code:

- ICD: N18.4, (alle mit eGFR ≤ 30 ml/min), N18.9

Metformin-ATC-Codes (keine der folgenden Codes):

- Monotherapie: A10BA02
- Kombinationstherapie: A10BD02, A10BD03, A10BD05, A10BD07, A10BD08, A10BD10, A10BD11, A10BD13, A10BD14, A10BD15, A10BD16, A10BD17, A10BD18, A10BD31

Chart Review:

- Angaben über den eGFR-Wert
- Angabe über Diabetes-Erkrankung
- Angabe blutzuckersenkende Medikamente (ja/nein) sowie Einnahme von Metformin

Zähler:	Anzahl der Patienten ≥70 Jahre mit CKD und eGFR < 30 ml/min/1,73 m^2 und Diabetes mellitus Typ 2, die Metformin verordnet bekommen haben
Nenner:	Anzahl der Patienten ≥70 Jahre mit CKD und eGFR < 30 ml/min/1,73 m^2 und Diabetes mellitus Typ 2

Hintergrund:

Metformin wird in der medikamentösen Therapie des Diabetes mellitus 2 eingesetzt. Die KDIGO empfiehlt Metformin bei Patienten mit CKD und einer eGFR < 30 ml/min/1,73 m^2 aufgrund der Arzneimittelsicherheit nicht einzusetzen [5]. Auch die DEGAM empfiehlt den Einsatz von Metformin nur bei einer eGFR > 30 ml/min/1,73 m^2. [3] Bei einer eingeschränkten Nierenfunktion und Diabetes ist unter Metformintherapie die Gefahr einer metabolischen Azidose (Lactatazidose) erhöht und deshalb Metformin kontraindiziert. (DEGAM-Leitlinie [3], [16])

Die Leitlinienempfehlung wurde umformuliert, da nicht alle Patienten mit Diabetes eine medikamentöse Therapie erhalten, sondern auch durch eine Lebensstilumstellung der HbA1C-Wert adäquat eingestellt werden kann. In diesem Fall würde Metformin nicht verordnet werden.<br><br>Der Indikator sollte optimalerweise bei 0% liegen.
Problem:<br><br>Für die Anwendung des Qualitätsindikators mithilfe von Routinedaten ist die genaue Verschlüsselung des CKD-Stadiums (ICD N18.4, eGFR < 30 ml/min/1,73 m^2 CKD 4) notwendig. Ein Teil der Patienten wird mit ICD N18.9 verschlüsselt, was zu einer Überschätzung der Versorgungsqualität führen kann.
Literatur:<br><br>„Patients without biguanide/ Diabetic CKD patients with eGFR <45 ml/min“ [17]<br><br>„Percentage of patients with CKD with a GFR < 30 ml/min./1.73m^2, in whom metformin is avoided/ is not prescribed“ [14]<br><br>KDIGO: "We recommend that metformin be continued in people with GFR ≥45 ml/min/1.73 m^2 (GFR categories G1-G3a); its use should be reviewed in those with GFR 30-44 ml/min/1,73 m^2 (GFR category G3b); and it should be discontinued in people with GFR <30 ml/min/1.73 m^2 (GFR categories G4-G5).“ [5]

20	Anteil der Patienten ≥70 Jahre mit CKD und Anämie (Hb < 6,8 mmol/l / <11 mg/dl), bei denen zur Überprüfung des Eisenstatus der Serumferritinwert mit Serumtransferrinsättigung bestimmt wurde.
Monitoring	
„Bei Patienten mit CKD und einem Hämoglobin < 11 mg/dl (< 6,8 mmol/l) sollten zur Überprüfung des Eisenstatus der Serumferritinwert in Kombination mit der Serumtransferrinsättigung (TSAT) erhoben werden.“ (DEGAM-Empfehlung 8.8 [3])	
☐ Routinedaten<br><br>☒ Chart Review	
Chart Review	
Zähler:	Anzahl der Patienten ≥70 Jahre mit CKD und Anämie (Hb < 6,8 mmol/l / < 11 mg/dl), bei denen der Serumferritinwert und die Serumtransferrinsättigung bestimmt wurde
Nenner:	Anzahl der Patienten ≥70 Jahre mit CKD und Anämie (Hb < 6,8 mmol/l / < 11 mg/dl)
Hintergrund:<br><br>Zur Überprüfung des Eisenhaushaltes ist es sinnvoll, bei erniedrigtem Hb den Serumferritinwert und die Serumtransferrinsättigung zu bestimmen. Bei einer Eisenverwertungsstörung ist der Ferritinwert normal bis erhöht und die Serumtransferrinsättigung erniedrigt.(DEGAM-Leitlinie [3])	
Problem:<br><br>Der Hämoglobinmesswert ist nicht in Routinedaten verfügbar, daher wird hier ausschließlich auf die Nutzung des Chart Reviews verwiesen.	

Bereits existierende QI (Literatur)

21	Anteil der Patienten ≥70 Jahre mit CKD und Hypertonie, die im Rahmen der Therapie ACE-Hemmer oder ARB erhalten.

Medikamentöse Therapie

„Patients on RAS inhibitors / CKD patients with hypertension who satisfied criteria of A and (B or C)

A: patients labeled with hypertension (ICD10)

B: CKD patients labeled with diabetes (ICD10)

C: non-DM CKD patients with proteinuria

Patients labeled with hyperkalemia are excluded " [17]

„Prescription of ACE inhibitor or angiotensin receptor blocker recorded in past year for patients

with CKD and hypertension with proteinuria" [9]

KDIGO: „We recommend that an ARB or ACE-I be used in both diabetic and non-diabetic adults with CKD and urine albumin excretion > 300 mg/24 hours (or equivalent*)." [5]

NICE: „Offer a low-cost renin–angiotensin system antagonist to people with CKD and:

- diabetes and an ACR of 3 mg/mmol or more (ACR category A2 or A3)
- hypertension and an ACR of 30 mg/mmol or more (ACR category A3)
- an ACR of 70 mg/mmol or more (irrespective of hypertension or cardiovascular disease)" [4]

☒ Routinedaten

☒ Chart Review

Routinedaten:

- ICD Hypertonie: I10-I15 Und
- ICD CKD: N18.1, N.18.2, N18.3, N18.4, N18.9 (nicht näher bezeichnet)
- ATC-Codes ACE: C09A, C09 B (und in Kombination)
- ATC Angiotensin-II-Rezeptor-Blocker (ARB): C09C, C09 D (in Kombination)

Zähler:	Anzahl der Patienten ≥70 Jahre mit CKD und Hypertonie (ICD I10-I15), die ACE-Hemmer oder ARB verordnet bekommen haben.
Nenner:	Anzahl der Patienten ≥70 Jahre mit CKD und Hypertonie (ICD I10-I15)

Hintergrund:

Erwartete Therapievorteile von ACE-Hemmern und ARB sind die Reduktion der Mortalität sowie die Reduktion der Proteinurie und Progression von CKD. [4, 12]<br><br>Es gibt Leitlinienempfehlungen (KDIGO [5], NICE [4]), die die Anwendung von ACE oder ARB auch bei Patienten mit CKD und Diabetes und Albuminurie (30-300 mg/24h) oder CKD und Albuminurie über 300 mg/24 h vorschlagen.
Problem<br><br>Für die Anwendung des Qualitätsindikators mithilfe von Routinedaten ist die genaue Verschlüsselung der Proteinurie notwendig, die oft nicht erfolgt. Daher kann dieser Aspekt nicht berücksichtigt werden.
Dieser QI wird auch vorgeschlagen von:<br><br>● NICE QOF: „The percentage of patients on the CKD register who have hypertension and proteinuria and who are currently being treated with renin-angiotensin system antagonists" *(NICE id code: NM84) [6]*<br>● ICES: „Percentage of patients with diabetes and albuminuria (moderately or severely increased ACR ≥ 3 mg/mmol) who were prescribed an angiotensin-converting enzyme (ACE) inhibitor or angiotensin II receptor blocker (ARB) unless a contraindication or adverse effects are recorded." [7]

<table>
<tr><td>22</td><td colspan="2">Anteil der Patienten ≥70 Jahre mit CKD, die in den letzten 12 Monaten eine Grippeschutzimpfung erhalten haben, falls nicht kontraindiziert.</td></tr>
<tr><td colspan="3">Monitoring</td></tr>
<tr><td colspan="3">“Percentage of patients with chronic kidney disease who received an influenza vaccine in the past year unless contraindicated.” (ICES [7])</td></tr>
<tr><td colspan="3">☒ Routinedaten

☒ Chart Review</td></tr>
<tr><td colspan="3">Routinedaten:

● Grippeschutzimpfung/Influenza Impfung: GOP 89111 (Standardimpfung, Personen ab 60 Jahre), GOP 89112 (Influenza Indikationsimpfung)
● Prävalenzindikator CKD</td></tr>
<tr><td colspan="2">Zähler:</td><td>Anzahl der Patienten ≥70 Jahre mit CKD und Influenza-Impfung in den letzten 12 Monaten</td></tr>
<tr><td colspan="2">Nenner:</td><td>Anzahl der Patienten ≥70 Jahre mit CKD</td></tr>
<tr><td colspan="3">Hintergrund: Die STIKO empfiehlt die Influenzaimpfung für Personen mit „erhöhter gesundheitlicher Gefährdung infolge eines Grundleidens"[18], dazu zählen auch Nierenkrankheiten. Diese Personen haben ein erhöhtes Risiko, schwere Krankheitsverläufe bei einer Infektion mit Influenza zu entwickeln. [18] Auch die KDIGO empfiehlt eine Impfung (KDIGO-Leitlinie [5]). Bei Patienten mit terminaler Niereninsuffizienz (ESRD) konnte ein positiver Effekt hinsichtlich Hospitalisation und Mortalität gezeigt werden. [19]</td></tr>
<tr><td colspan="3">Problem: Die Entscheidung zur Grippeschutzimpfung liegt beim Patienten. Der Arzt/die Ärztin kann lediglich aufklären. Kontraindikationen sind schwer operationalisierbar.</td></tr>
</table>

23	Anteil der Patienten ≥70 Jahre mit CKD, die über mindestens 2 Quartale mehrfach (≥2) gleichzeitig ACE Hemmer und ARB (Angiotensin-II-Rezeptorblocker) innerhalb von 3 Quartalen nach dem Quartal mit der ersten Doppelverordnung verordnet bekommen.

Medikamentöse Therapie

„Percentage of patients with chronic kidney disease simultaneously receiving both an ACE inhibitor and an ARB.“ (ICES [7])

☒ Routinedaten

☒ Chart Review

Routinedaten:

- Prävalenzindikator CKD
- ACE Hemmer ATC Code: C09A, C09B, C09BB, C09BX
- ARB ATC Code: C09C, C09D

Zähler:	Anzahl der Patienten ≥70 Jahre mit CKD und gleichzeitiger Verschreibung von ACE Hemmern und ARB in mind. 2 Quartalen innerhalb von 3 Quartalen nach der ersten Doppelverordnung
Nenner:	Anzahl der Patienten ≥70 mit CKD im gleichen Beobachtungszeitraum wie Zähler

Hintergrund:

Die Kombination beider Medikamente (ACE-Hemmer und ARB) führt zu vermehrten Nebenwirkungen und zeigte keinen positiven Effekt auf die Mortalität. [20, 21]

Problem: Bei Unverträglichkeit eines der Medikamente (ACE-Hemmer oder ARB) kann der Arzt/die Ärztin auf die jeweilige andere Medikamentengruppe umstellen. Das würde bedeuten, dass die Verschreibung von beiden Medikamenten in einem Quartal erfolgen kann. Um sicher zu gehen, dass es sich nicht um eine Medikamentenumstellung handelt, werden hier mind. 2 Quartale berücksichtigt, in der Annahme, dass erst eine weitere zeitlich überlappende Verordnung eine gleichzeitige Einnahme von ACE-Hemmern und ARB ausreichend sicher belegt.

1 Literatur

1. SoSci Survey GmbH SoSci Survey - die Lösung für eine professionelle Onlinebefragung. https://www.soscisurvey.de/. Zugegriffen: 27 Aug 2022
2. Reiter A, Fischer B, Kötting J, Geraedts M, Jäckel WH, Döbler K (2007) QUALIFY: Ein Instrument zur Bewertung von Qualitätsindikatoren (QUALIFY--a tool for assessing quality indicators). Z Arztl Fortbild Qualitatssich 101(10):683–688. https://doi.org/10.1016/j.zgesun.2007.11.003
3. Weckmann G, Chenot J-F, Stracke S (2019) Versorgung von Patienten mit chronischer nicht-dialysepflichtiger Nierenerkrankung in der Hausarztpraxis: S3 Leitlinie. https://www.awmf.org/leitlinien/detail/ll/053-048.html. Zugegriffen: 18 Mar 2022
4. NICE National Institute for Health and Care Excellence (2014) Chronic kidney disease in adults: assessment and management: Clinical guideline. https://www.nice.org.uk/guidance/cg182. Zugegriffen: 20 Jun 2021
5. Kidney Disease Improving Global Outcomes (2013) KDIGO 2012 Clinical Practice Guideline for the Evaluation and Management of Chronic Kidney Disease. https://kdigo.org/guidelines/. Zugegriffen: 14 Oct 2022
6. NICE National Institute for Health and Care Excellence NICE Quality and Outcomes Framework indicator. https://www.nice.org.uk/standards-and-indicators/qofindicators. Zugegriffen: 06.2021
7. Tu K, Bevan L, Hunter K, Rogers J, Young J, Nesrallah G (2017) Quality indicators for the detection and management of chronic kidney disease in primary care in Canada derived from a modified Delphi panel approach. CMAJ Open 5(1):E74-E81. https://doi.org/10.9778/cmajo.20160113
8. Bundesärztekammer (BÄK), Kassenärztliche Bundesvereinigung (KBV), Arbeitsgemeinschaft der Wissenschaftlichen Medizinischen Fachgesellschaften AWMF (2010, zuletzt verändert: 2015) Nationale VersorgungsLeitlinie: Nierenerkrankungen bei Diabetes im Erwachsenenalter - Langfassung, 1. Auflage. Version 6. https://www.leitlinien.de/. Zugegriffen: 13 Oct 2022
9. Litvin CB, Ornstein SM (2014) Quality Indicators for Primary Care: An Example for Chronic Kidney Disease. J Ambul Care Manage 37(2):171–178. https://doi.org/10.1097/JAC.0000000000000015
10. Williams B, Mancia G, Spiering W et al. (2018) 2018 ESC/ESH Guidelines for the management of arterial hypertension. Eur Heart J 39(33):3021–3104. https://doi.org/10.1093/eurheartj/ehy339
11. Ludt S, Angelow A, Baum E et al. (2017) Hausärztliche Risikoberatung zur kardiovaskulären Prävention: S3-Leitlinie. https://www.awmf.org/leitlinien/detail/ll/053-024.html. Zugegriffen: 18 Mar 2022
12. Kidney Disease Improving Global Outcomes (KDIGO) Blood Pressure Work Group (2021) KDIGO 2021 Clinical Practice Guideline for the Management of Blood Pressure in Chronic Kidney Disease. Kidney Int 99:S1-S87. https://doi.org/10.1016/j.kint.2020.11.003
13. Whelton PK, Carey RM, Aronow WS et al. (2018) 2017 ACC/AHA/AAPA/ABC/ACPM/AGS/APhA/ASH/ASPC/NMA/PCNA Guideline for the Prevention, Detection, Evaluation, and Management of High Blood Pressure in Adults: A Report of the American College of Cardiology/American Heart Association Task Force on Clinical Practice Guidelines. Hypertension 71(6):e13-e115. https://doi.org/10.1161/HYP.0000000000000065

14. van den Bulck SA, Vankrunkelsven P, Goderis G et al. (2020) Developing quality indicators for Chronic Kidney Disease in primary care, extractable from the Electronic Medical Record. A Rand-modified Delphi method. BMC Nephrol 21:161. https://doi.org/10.1186/s12882-020-01788-8
15. Bello AK, Ronksley PE, Tangri N et al. (2019) Quality of Chronic Kidney Disease Management in Canadian Primary Care. JAMA Netw Open 2(9):e1910704. https://doi.org/10.1001/jamanetworkopen.2019.10704
16. Lazarus B, Wu A, Shin J-I et al. (2018) Association of Metformin Use With Risk of Lactic Acidosis Across the Range of Kidney Function: A Community-Based Cohort Study. JAMA Intern Med 178(7):903–910. https://doi.org/10.1001/jamainternmed.2018.0292
17. Fukuma S, Shimizu S, Niihata K et al. (2016) Development of quality indicators for care of chronic kidney disease in the primary care setting using electronic health data: a RAND-modified Delphi method. Clin Exp Nephrol 21:247–256. https://doi.org/10.1007/s10157-016-1274-8
18. Remschmidt C, Harder T, Wichmann O et al. (2016) Hintergrundpapier der STIKO: Evaluation der bestehenden Influenzaimpfempfehlung für Indikationsgruppen und für Senioren (Standardimpfung ab 60 Jahren). Bundesgesundheitsblatt Gesundheitsforschung Gesundheitsschutz 59:1606–1622. https://doi.org/10.1007/s00103-016-2467-8
19. Gilbertson DT, Unruh M, McBean AM, Kausz AT, Snyder JJ, Collins AJ (2003) Influenza vaccine delivery and effectiveness in end-stage renal disease. Kidney Int 63(2):738–743. https://doi.org/10.1046/j.1523-1755.2003.00787.x
20. Makani H, Bangalore S, Desouza KA, Shah A, Messerli FH (2013) Efficacy and safety of dual blockade of the renin-angiotensin system: meta-analysis of randomised trials. BMJ 346:f360. https://doi.org/10.1136/bmj.f360
21. The ONTARGET Investigators, Yusuf, S, Teo KK, Pogue J et al. (2008) Telmisartan, Ramipril, or Both in Patients at High Risk for Vascular Events. N Engl J Med 358(15):1547–1559. https://doi.org/10.1056/NEJMoa0801317
